# Classification of Plant Associated Bacteria Using RIF, a Computationally Derived DNA Marker

**DOI:** 10.1371/journal.pone.0018496

**Published:** 2011-04-21

**Authors:** Kevin L. Schneider, Glorimar Marrero, Anne M. Alvarez, Gernot G. Presting

**Affiliations:** 1 Molecular Biosciences and Bioengineering, University of Hawaii at Manoa, Honolulu, Hawaii, United States of America; 2 Plant and Environmental Protection Sciences, University of Hawaii at Manoa, Honolulu, Hawaii, United States of America; Charité-University Medicine Berlin, Germany

## Abstract

A DNA marker that distinguishes plant associated bacteria at the species level and below was derived by comparing six sequenced genomes of *Xanthomonas*, a genus that contains many important phytopathogens. This DNA marker comprises a portion of the *dnaA* replication initiation factor (RIF). Unlike the rRNA genes, *dnaA* is a single copy gene in the vast majority of sequenced bacterial genomes, and amplification of RIF requires genus-specific primers. *In silico* analysis revealed that RIF has equal or greater ability to differentiate closely related species of *Xanthomonas* than the widely used ribosomal intergenic spacer region (ITS). Furthermore, in a set of 263 *Xanthomonas*, *Ralstonia* and *Clavibacter* strains, the RIF marker was directly sequenced in both directions with a success rate approximately 16% higher than that for ITS. RIF frameworks for *Xanthomonas*, *Ralstonia* and *Clavibacter* were constructed using 682 reference strains representing different species, subspecies, pathovars, races, hosts and geographic regions, and contain a total of 109 different RIF sequences. RIF sequences showed subspecific groupings but did not place strains of *X. campestris* or *X. axonopodis* into currently named pathovars nor *R. solanacearum* strains into their respective races, confirming previous conclusions that pathovar and race designations do not necessarily reflect genetic relationships. The RIF marker also was sequenced for 24 reference strains from three genera in the Enterobacteriaceae: *Pectobacterium*, *Pantoea* and *Dickeya*. RIF sequences of 70 previously uncharacterized strains of *Ralstonia*, *Clavibacter*, *Pectobacterium* and *Dickeya* matched, or were similar to, those of known reference strains, illustrating the utility of the frameworks to classify bacteria below the species level and rapidly match unknown isolates to reference strains. The RIF sequence frameworks are available at the online RIF database, RIFdb, and can be queried for diagnostic purposes with RIF sequences obtained from unknown strains in both chromatogram and FASTA format.

## Introduction

Bacterial phytopathogens cause billions of dollars in crop losses annually (as summarized by Baker et al. [1]). Rapid and accurate identification of bacterial phytopathogens is essential for modern agriculture, as it permits informed decision making with respect to potentially costly but necessary control methods that include quarantine and the destruction of infected plant material in the field [2], [3]. The genus *Xanthomonas* contains some of the most important plant pathogenic bacteria that together infect over 392 hosts [4]. The bacterial genera *Clavibacter*, *Ralstonia*, *Pseudomonas*, *Dickeya*, *Erwinia*, *Pantoea*, *Pectobacterium*, and *Xylella* also contain species of important plant pathogens [5]–[12]. The phytopathogens *X. oryzae* pv. *oryzae* and *R. solanacearum* (Rs) race 3 biovar 2 are considered severe threats to US agriculture [3].

Bacterial taxonomic designations are derived from a diverse set of classification methods. Bacterial strains have been reclassified to different subspecies, species and even genera as diagnostic methods have evolved [6], [10]–[27]. For example, the historical designations of *Xanthomonas* pathovars and *Ralstonia solanacearum* races, based on the plant host and symptoms produced, are being replaced by modern DNA based methods [17] that have been used to reclassify some pathovars of *X. axonopodis* into different species and subspecies [15], [16] (e.g. *X. axonopodis* pv. *vesicatoria* type C was reclassified as *X. perforan*s based on DNA-DNA hybridization). Likewise, 16S rDNA sequencing, DNA-DNA hybridization and other genetic and phenotypic traits have been used to reclassify some species of *Pseudomonas*, *X. maltophilia*, *Erwinia chrysanthemi*, *E. carotovora* and *E. herbicola* into species of *Ralstonia*, *Stenotrophomonas maltophilia*, *Dickeya*, *Pectobacterium* and *Pantoea agglomerans*, respectively [9]–[12], [28].

DNA-based species identification methods are robust and efficient, as evidenced by the large number of ongoing DNA barcoding efforts [29]. In DNA barcoding, sequences of a single DNA marker region are associated with reference strains that have been identified by taxonomists. The specific marker used is tailored to the range of target organisms. For animals, a region of the rapidly evolving mitochondrial cytochrome *c* oxidase (*CO1*) gene has been designated the DNA barcode region [30]. For the identification of algae, the universal plastid amplicon, a computationally derived region of the chloroplast genome [31] that can be amplified in photosynthetic organisms ranging from cyanobacteria to red, brown, golden and green algae as well as higher plants [32], has been proposed as a DNA barcode and proven to be practical in both biodiversity surveys and environmental sampling [33], [34].

The speed of PCR and the low cost of sequencing of the resulting amplicons have made bacterial identification based on DNA markers a viable method that is replacing earlier classification methods based on time-consuming biochemical or biological assays. The most commonly used markers are derived from ribosomal genes, in part because these can be amplified in the majority of species using universal primers [20]. 16S rDNA sequences have been determined for more than one million individual strains and environmental samples at http://rdp.cme.msu.edu [35]. Similarly, over 18,000 sequences of the internal transcribed spacer (ITS), which lies between the 16S and 23S rRNA genes, are available in GenBank [36]. Sequences from these two markers have been used to identify phytopathogens and study population diversity [37], [38], [39]. However, 16S rDNA sequences do not always resolve species within a genus, and even the more variable ITS sequences fail to resolve many genera below the species level [37]. Kang et al. [39] have shown that multiple copies of the 16S and 23S rRNA genes exist in over 80% (639 of 782 strains) of Gram^+^ and Gram^−^ bacteria examined. Direct sequencing of rDNA amplicons from a genome containing different alleles (as observed for 415 of 782 strains by Kang et al. [39]) results in poor sequence quality. This is currently overcome by one of two time-consuming procedures, excising individual amplicons from agarose gels [40] if the alleles differ in length, or by cloning the amplicons.

DNA markers designed from housekeeping genes have been used singly or in combination to determine phylogenetic relationships of bacteria [21]–[27]. They generally require the use of genus-specific primers for amplification. Housekeeping genes are under stabilizing selection and are therefore expected to more accurately portray the genetic relationships among strains than genes that are under positive selection [41]. Genes under stabilizing selection may also be less prone to lateral transfer than other genes, such as those involved in pathogenicity. Nonetheless, even housekeeping genes may be transferred laterally as evidenced by recombination events within the *gyrB* gene of *Vibrio* species [42].

An ideal DNA marker for bacterial classification and identification is more variable than the rDNA regions, present in all target organisms as a single copy per genome, unlikely to undergo horizontal gene transfer, and amplifiable with universal primers. Attempts to design universal primers from sequences other than the rDNA regions have been unsuccessful ([43] and Supplemental Text S1), likely due to the great diversity and rapidly evolving nature of bacterial genomes.

We used six completely sequenced *Xanthomonas* genomes [44]–[47] representing four species to computationally identify a marker to classify closely related strains of bacteria. The replication initiation factor (RIF) marker, a region of the single-copy *dnaA* gene, was the best marker to distinguish closely related *Xanthomonas* strains. We sequenced RIF for a subset of 706 strains of six plant pathogenic genera from the Pacific Bacterial Collection (PBC) and the International Collection of Microorganisms from Plants (ICMP) at Landcare Research of New Zealand, representing a diverse range of hosts, geographic origins, subspecies, pathovars and races. These RIF frameworks can be queried online at http://genomics.hawaii.edu/RIFdb. Our results indicate that RIF is a suitable marker for the classification of strains from the six genera used in this study, complements other DNA markers in *Xanthomonas* MLSA studies, and may be expanded to other bacterial genera, the majority of which contain a single copy of the *dnaA* gene.

## Results

### Identification of RIF, a marker with subspecific resolution

**Figure 1:**
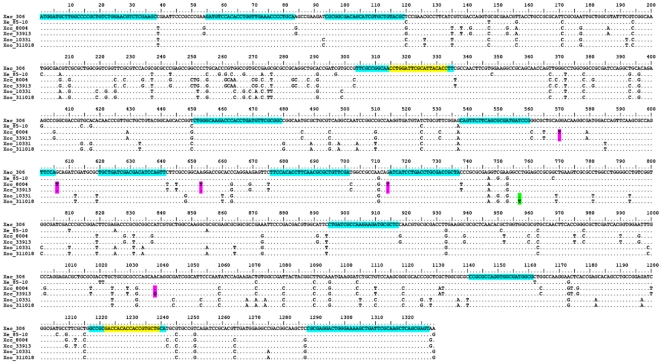
Alignment of the *dnaA* gene from six *Xanthomonas* strains. The *dnaA* genes of *X. campestris* pv. *campestris* strains differ by 5 SNPs (pink highlight) and those of two *X. oryzae* pv. *oryzae* strains (green highlight) differ by 1 SNP. Conserved 20^+^-mers in all six *Xanthomonas* genomes are highlighted in blue. The only set of primers that flank all SNPs, have a melting temperature of 60±2°C and a G+C content of 50–60%, end in a G or C and have no complementary bases at the ends, are highlighted in yellow.

**Figure 2:**
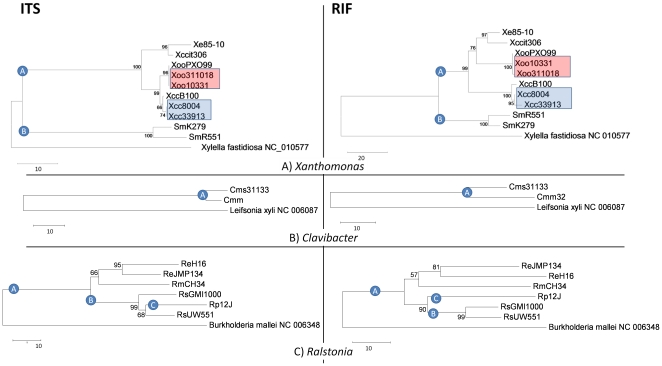
Neighbor-joining cladograms for *Xanthomonas*, *Clavibacter* and *Ralstonia* using ITS and RIF sequences. RIF resolves closely related strains of *Xanthomonas* that remain unresolved with ITS. The neighbor-joining cladograms for A) *Xanthomonas*, B) *Clavibacter* and C) *Ralstonia* using the ITS and RIF markers from fully sequenced strains in GenBank (Table 1). Bootstrap values >50% (shown at the node) are expressed as a percentage of 5,000 replicates. Red boxes contain two *X. oryzae* pv. *oryzae* strains resolved by one SNP between RIF sequences and zero SNPs between ITS sequences. Likewise, blue boxes contain two *X. campestris* pv. *campestris* strains resolved by five SNPs between RIF sequences and zero SNPs between ITS sequences. The RIF and ITS sequences of Xoo strains 10331 and PXO99 are identical. *Xylella fastidiosa*, *Burkholderia mallei* and *Leifsonia xyli* are included as outgroups.

A marker for the classification of bacteria was computationally identified using fifteen completely sequenced genomes representing six genera of plant pathogenic bacteria (asterisk in Table 1), including two *Xanthomonas oryzae* pv. *oryzae* strains (Xoo), two *X. campestris* pv. *campestris* strains (Xcc), one *X. citri* strain and one *X. euvesicatoria* strain (see Methods for details). In brief, 16,362 20^+^-mers (i.e. potential priming sites) were conserved between all six *Xanthomonas* genomes. Analysis of the Xcc strain 8004 and 33913 genomes revealed *dnaA* (Figure 1) as the sole gene that a) contains potential amplicons with more than one SNP (a total of five), b) has greater resolution than the ITS for the six *Xanthomonas* strains (Table 2) and c) is present as a single copy gene in all fifteen genomes. Two conserved 20^+^-mers that flank all SNPs were used to design primers and define the RIF marker. One SNP in the RIF marker also resolved the two Xoo strains with identical ITS sequences (Figure 2A). Primer pairs to amplify and sequence the RIF marker (Table 3) for *Ralstonia* and *Clavibacter* were designed within 60 nt of the *Xanthomonas* primers (Supplemental Figure S1).

**Table 1:** Completely sequenced genomes discussed in this study.

Strain Name	Accession	Abbreviations
*Clavibacter michiganensis* subsp. *michiganensis* NCPPB 32*	NC_009480.1	Cmm_32
*Clavibacter michiganensis* subsp. *sepedonicus* ATCC 33113*	NC_010407.1	Cms_33113
*Erwinia amylovora* Ea273*	NC_013971.1	Ea_273
*Erwinia chrysanthemi* (*Dickeya dadantii*) 3937*	CP002038	Dd_3937
*Erwinia carotovora* subsp. *atroseptica* (*Pectobacterium atrosepticum*) SCRI1043*	NC_004547.2	Pa_1043
*Erwinia pyrifoliae* Ep1/96	NC_012214.1	Ep_1/96
*Erwinia tasmaniensis* Et1/99	NC_010694.1	Et_1/99
*Dickeya zeae* Ech1591	NC_012912.1	Dz_1591
*Dickeya dadantii* Ech703	NC_012880.1	Dd_703
*Dickeya dadantii* Ech586	NC_013592.1	Dd_586
*Pectobacterium wasabiae* Wpp163	NC_013421.1	Pw_Wpp163
*Pectobacterium carotovorum* subsp. *carotovorum* PccPC1	NC_012917.1	Pcc_PC1
*Ralstonia eutropha* H16*	NC_008313.1	Re_H16
*Ralstonia eutropha* JMP134*	NC_007347.1	Re_JMP134
*Ralstonia metallidurans* CH34*	NC_007973.1	Rm_CH34
*Ralstonia solanacearum* GMI1000*	NC_003295.1	Rs_GMI1000
*Ralstonia pickettii* 12J	NC_010682.1	Rp_12J
*Xanthomonas campestris* pv. *campestris* B100	NC_010688.1	Xcc_B100
*Xanthomonas campestris* pv. *campestris* ATCC 33913*	NC_003902.1	Xcc_33913
*Xanthomonas campestris* pv. *campestris* 8004*	NC_007086.1	Xcc_8004
*Xanthomonas campestris* pv. *vesicatoria* 85–10 (*Xanthomonas euvesicatoria*)*	NC_007508.1	Xe_85-10
*Xanthomonas oryzae* pv. *oryzae* PXO99	NC_010717.1	Xoo_PXO99
*Xanthomonas oryzae* pv. *oryzae* KACC10331*	NC_006834.1	Xoo_10331
*Xanthomonas oryzae* pv. *oryzae* MAFF 311018*	NC_007705.1	Xoo_311018
*Xanthomonas axonopodis* pv. *citri* 306 (*Xanthomonas citri* subsp. *citri*)*	NC_003919.1	Xccit_306
*Stenotrophomonas maltophilia* K279a	NC_019043.1	Sm_279
*Stenotrophomonas maltophilia* R551-3	NC_011071.1	Sm_R551

*Set of 15 genomes used for the identification of RIF.

**Table 2:** Genes in Xoo_311018 and Xcc_33913 containing regions that match criteria for potential markers.

Strain^a^	Location	Gene Name^b^	Number of regions meeting marker criteria^c^	Greater resolution than the ITS	Complete gene is present in all 15 strains^d^
Xoo_311018	2251324–2254065	XOO2036	1	no	no
Xoo_311018	4934856–4938381	XOO4369	4	no	no
Xoo_311018	552362–553945	XOO0506	13	no	no
Xoo_311018	4512534–4515648	XOO4004	13	no	no
Xoo_311018	4897866–4899412	XOO4332	1	no	no
Xoo_311018	8754–9959	XOO0006	3	no	no
Xoo_311018	4906929–4908174	XOO4342	5	no	no
Xoo_311018	4937637–4939494	XOO4370	32	yes^f^	no
Xcc_33913	2175534–2176610	XCC1866	1	no	no
Xcc_33913	1–1370	XCC0001^e^	27	yes^f^	yes
Xcc_33913	4748693–4749484	XCC4004	4	no	no
Xcc_33913	4506913–4510053	XCC3789	16	yes	no
Xcc_33913	4516069–4517528	XCC3796	1	no	no
Xcc_33913	4513098–4514465	XCC3793	3	no	no
Xcc_33913	4749117–4751483	XCC4005	6	no	no

a - Xoo_10331 was compared to Xoo_311018; Xcc_8004 was compared to Xcc_33913.

b - Genes identified in Xoo_311018 do not overlap with those identified in Xcc_33913.

c - Marker criteria: conserved 20^+^-mers are separated by >550 nt and produce an amplicon <1000 nt, flank 2 or more SNPs between Xoo_10331 and Xoo_311018 or Xcc_8004 and Xcc_33913.

d - Strains are listed in Table 1 with an asterisk.

e - Gene XCC0001 met all criteria for use as a potential marker.

f - Orthologs differ by a single SNP.

**Table 3:** RIF primers developed in this study.

Genus^a^	Forward Primer	Reverse Primer
*Clavibacter*	5-TACGGCTTCGACACCTTCG-3	5-CGGTGATCTTCTTGTTGGCG-3
*Dickeya* b	5-CCTATCGYTCGAACGTGAA-3	5-CTGCTCGATTTTGCGGCAG-3
*Dickeya* c	5-CACACYTATCGYTCCAAYGT-3	5-TGTCGTGACTTTCYTCRCGC-3
*Pectobacterium* b	5-TACCGTTCCAATGTGAACCC-3	5-AAATCTTCTTTGATGTCGTGG-3
*Pectobacterium* c	5-ATGTGAACCCSAAACATACGT-3	5-TTCACGCAACTGCTCAATCTT-3
*Ralstonia*	5-TCRCGSCTGAACCSATCCT-3	5-TTGAGCTGSGCGTCCTTGC-3
*Xanthomonas*	5-CAGCACGGTGGTGTGGTC-3	5-CCTGGATTCGCATTACACC-3
*Pseudomonas* d	5-CTBAAGCACACCAGYTAYC-3	5-TCGCATTAGTTTATCCCAGTC-3
*Xylella* e	5-GAGGGACGAAGCAATCAACT-3	5-CAGCAGGTTCTTGTAGTCCT-3

a No genome sequence was available for *Pantoea agglomerans* when these primers were developed.

b Primer pairs for three genera of Enterobacteriaceae were initially developed by comparison of the *dnaA* gene of *Dickeya*, *Pectobacterium* and *Erwinia* available in 2007 (Table 1, Supplemental Figure S1). Primers were developed using the criteria applied for selection of the *Clavibacter* and *Ralstonia* primers and alignment of the respective *dnaA* gene (see methods). The initial *Pectobacterium* and *Dickeya* primers failed to produce amplicons for two strains of *Dickeya* (one *D. chrysanthemi* and one *D. paradisiaca*), three strains of *Pantoea* (one that also failed to amplify with the ITS marker) and nine strains of *Pectobacterium* (eight of which failed to amplify with the ITS marker). *Pectobacterium* strains K0509, K0522 and K0574 and *Pantoea* strains required an annealing temperature of 51°C.

c Primer sets were derived using newly sequenced strains of *Dickeya* (Dz_1591, Dd_703) and *Pectobacterium* (Pc_PCC1 and Pw_Wpp163). The *Dickeya* primers amplified all nine reference *Dickeya* strains. The new *Pectobacterium* primers no longer amplified any *Pantoea* strains at 51°C and they amplified strain K0522, K0574 and one additional *Pectobacterium* strain, K0510, but still did not amplify strain K0509 at 61°C.

d Primers developed from *Pseudomonas* accessions NZ_CH482384, NZ_CM001020, NC_011770, NC_009656, NC_002516, NC_008463, NC_008027, NC_004129, NC_012660, NZ_CM001025, NC_009439, NC_009512, NC_010322, NC_002947, NC_010501, NZ_GG774590, NZ_DS997060, NC_005773, NC_007005, NZ_GG700508, NC_004578, NZ_GG699506 and NC_009434.

e Primers developed from *Xylella fastidiosa* accessions CP002165, CP001011, AE009442, CP000941 and AE003849.

### The RIF marker is present as a single copy in the majority of bacterial genomes

The copy number of the *dnaA* gene was determined for 1,067 completed bacterial genomes. The *dnaA* gene is present once in 1,016 (95.2%) of 1,067 bacterial genomes, twice in 40 (3.7%) bacterial genomes and absent in eleven (1.0%) genomes (Supplemental Table S1). A single copy of the *dnaA* gene is present in all sequenced genomes of 366 genera, including all sequenced plant associated bacteria. Strains lacking the *dnaA* gene include seven genera of insect endosymbionts (*Blattabacterium*, *Blockmannia*, *Carsonella*, *Hodgkinia*, *Riesia*, *Sulcia*, and *Wigglesworthia*). All sequenced genomes of twelve genera contain two copies of the *dnaA* gene, including obligate intracellular animal pathogens (13 *Chlamydia*, 8 *Chlamydophila*, 1 *Lawsonia*, and 1 *Parachlamydia*), a rumen-associated bacterium (1 *Fibrobacter*), sulfate reducing bacteria (1 *Desulfohalobium*, 1 *Desulfomicrobium* and 7 *Desulfovibrio*), a metal reducing bacterium (1 *Thermincola*), a soil-associated heparin reducing bacterium (1 *Pedobacter*), a saltern crystallizer pond-associated bacterium (1 *Salinibacter*) and water-associated bacteria (2 *Pirellula*). Two genera, *Mycobacterium* and *Mycoplasma*, contain only one genome with two copies of *dnaA*, but nineteen and twenty strains, respectively, with a single copy of *dnaA*. Therefore, RIF may still be a useful marker for these two genera.

### The RIF marker is unlikely to undergo horizontal gene transfer

Analysis of all available complete bacterial genomes revealed little evidence for horizontal transfer of *dnaA* between genera of bacteria or species of *Xanthomonas*. First, similar phylogenetic trees (data not shown) were constructed from the DnaA proteins and 16S rDNA regions of 1,016 genomes with a single copy of *dnaA*. Strains were placed in similar clades on both trees, consistent with a gene that has not undergone horizontal transfer between genera. Second, the phylogenetic tree constructed from the RIF marker of six *Xanthomonas* strains (Figure 2A) was similar to the dominant phylogenetic tree constructed from 229 *Xanthomonas* proteins present in all six sequenced strains [48]. These cladograms were representative of genetic regions that are unlikely to have been horizontally transferred between species of *Xanthomonas*. However, a subsequent analysis of 210 Rs strains for which we sequenced the *dnaA*, ITS and *egl* markers indicates that at least one of these may have undergone horizontal gene transfer (HGT) (see Rs results sections below).

### Comparison of the RIF and ITS markers in sequenced reference genomes

RIF sequences of *Xanthomonas* and *C. michiganensis* reference strains contained more nucleotide polymorphisms than their corresponding ITS sequences. The average distance between eight strains of *Xanthomonas* representing four species (clade A in Figure 2A) was three times greater with RIF than with ITS (33.6 nt differences between seven unique RIF sequences vs. 11.3 nt nucleotide differences between five unique ITS sequences). A comparison of two sequenced subspecies of *C. michiganensis* (Figure 2B) revealed twice as many polymorphic nucleotides in the RIF marker as in the ITS marker (31 vs. 14 nucleotide differences).

The average distance between the six *Ralstonia* strains representing four species (clade A in Figure 2C) was similar with both RIF and ITS markers (50.5 and 54.8 nucleotide differences, respectively). Nevertheless, RIF separated *R. solanacearum* strains GMI1000 and UW551 from *R. pickettii* strain 12J (clades B and C in Figure 2C) while the ITS did not (clade B). ITS resulted in interspecies (Rs-Rp) distances (35 nt) similar to intraspecies (Rs-Rs) distances (26 nt) while RIF showed a clear separation between interspecies distances (42 nt) and intraspecies distances (18 nt).

For all three genera RIF yielded more consistent results with the same or greater number of different sequences than ITS.

### Practicability of ITS and RIF markers as assessed by de novo sequencing of 263 PBC accessions

The RIF markers was amplified and directly sequenced in both directions using genus-specific primers (Table 3) and automatically base-called [49] and assembled [50] for 263 of the 840 *Xanthomonas*, *Ralstonia* and *Clavibacter* accessions of the PBC (Supplemental Table S2). Likewise, the ITS marker was amplified and directly sequenced in both directions using universal primers for the same set of strains. The length of the ITS sequences for the three genera ranged from 504–590 bp and that of the RIF sequences ranged from 654–700 bp. The majority of polymorphisms between RIF markers occurred in the wobble positions (data not shown). The success rate of high-quality direct sequence generation (i.e. overlapping sequence from both ends) for the 263 accessions was 1.22 times greater (Table 4) for RIF (248 sequences = 94%) than for ITS (205 sequences = 78%) and 16% of the accessions that yielded no ITS sequence produced RIF sequence (Supplemental Table S2). In addition, seven *Xanthomonas* and three *Ralstonia* accessions yielded a single RIF read in either the forward or reverse direction (Table 4).

**Table 4:** RIF sequencing success rate for *Xanthomonas*, *Ralstonia* and *Clavibacter* is higher than ITS.

	*Xanthomonas*	*Ralstonia*	*Clavibacter*
Total strains	120	120	23
ITS amplicon	120	119	23
ITS sequence	**87**	**99**	**19**
RIF amplicon	119	117	23
RIF sequence	**110**	**115**	**23**
RIF amplicon, no ITS amplicon	0	2	0
RIF contig, no ITS contig	15	17	4
ITS amplicon, no RIF amplicon	1	6	0
ITS contig, no RIF contig	2	2	0
ITS amplicon, no ITS contig	33	20	4
No ITS contig but ITS read	18	15	1
RIF amplicon, no RIF contig	9	4	0
No RIF contig, but RIF read	7	3	0
Size of ITS sequence (nt)	557–590	574–590	504–505
Size of RIF sequence (nt)	700	654	680
Number of strains with both the ITS and RIF sequenced	84	97	19
ITS average nucleotide differences*	29.3	16.1	10.5
RIF average nucleotide differences*	44.8	11.1	23.8
Number of different ITS sequences*	18	10	11
Number of different RIF sequences*	26	13	8

RIF and ITS marker characteristics for a set of 263 strains from the PBC. Sequence contigs are assembled from read pairs. The total number of RIF and ITS sequences are in bold.

*- Computed using a single representative for each unique sequence.

The 200 strains for which both ITS and RIF sequences could be obtained were compared using unrooted trees (Supplemental Figures S2, S3, S4). RIF differentiated a greater number of *Xanthomonas* and *R. solanacearum* accessions than ITS (Table 4). However, the opposite was true for *C. michiganensis* subsp. *michiganensis*. RIF and ITS markers formed similar groupings for *C. michiganensis* (Supplemental Figure S4), but the RIF and ITS groupings were different for *Xanthomonas* and *Ralstonia*. Two *X. campestri*s strains were placed outside of the *X. campestris* clade and one *X. axonopodis* strain was placed outside of the main *X. axonopodis* clade in the ITS tree, but not the RIF tree (asterisk in Supplemental Figure S2). For *Ralstonia*, strains grouped in one clade with the ITS marker were not grouped in the same clade with RIF (e.g., K0018/K0190 and K0024/K0157 in Supplemental Figure S3). In addition, although the Rs ITS clades were separated by a greater distance than the RIF clades, the sequence diversity within an ITS clade was less than that within a RIF clade (Supplemental Figure S3). *R. pickettii* was placed outside the Rs clade in the RIF but not the ITS tree. The different groupings observed for the RIF and ITS markers of some Rs strains indicate that one of the markers, most likely ITS, has undergone HGT. The higher sequencing success rate and more reliable groupings obtained with RIF encouraged us to exhaustively sample the sequence diversity present in the PBC and ICMP to build more complete RIF reference frameworks.

### Building RIF frameworks from sequences of 682 characterized accessions of *Xanthomonas*, *Ralstonia solanacearum* and *Clavibacter michiganensis*

Only characterized strains in the PBC and ICMP were used as reference strains to construct RIF frameworks for the purpose of classifying unknowns. In addition to the 263 strains above we attempted to sequence RIF from an additional 452 characterized PBC strains (715 strains total) that had been collected over a span of 37 years, and 84 *Xanthomonas* strains from the ICMP (Table 5 and Supplemental Table S2). In total, the RIF marker was successfully sequenced for 682 (85.3%) of 799 characterized strains and yielded 109 different DNA barcodes for classifying isolates of *Xanthomonas*, *R. solanacearum*, and *C. michiganensis* with respect to their RIF genotype (Table 5).

**Table 5:** RIF sequences for *Xanthomonas*, *Ralstonia* and *Clavibacter*.

	*Xanthomonas*	*Ralstonia*	*Clavibacter*
Total strains^a^ (characterized PBC/ICMP reference strains)	444 (361 PBC/84 ICMP)	248 (239)	126 (115)
RIF amplicon (characterized PBC/ICMP reference strains)	383 (340 PBC/43 ICMP)	228 (217)	125 (114)
*egl* amplicon	N/A	198	N/A
*egl* no amplicon	N/A	34	N/A
RIF sequence (characterized PBC/ICMP reference strains)	358 (**315** PBC/**43** ICMP^a^)	219(**210** b)	125 (**114**)
*egl* sequence	N/A	159/191^c^	N/A
RIF amplicon, no RIF sequence	29 (29 PBC/0 ICMP)	10	1
No RIF contig, but RIF read	31 (31 PBC/0 ICMP)	6	1
*egl* amplicon, no *egl* sequence	N/A	32	N/A
No *egl* contig, but *egl* read	N/A	19^d^	N/A
Size of RIF sequence (nt)	558	617	660
Size of *egl* sequence (nt)	N/A	495	N/A
RIF average nucleotide differences*	44	12.9	18.6
*egl* average nucleotide differences*	N/A	25.3	N/A
Number of different RIF sequences			
(characterized PBC/ICMP reference strains)*	82 (82)	17 (17)	11 (10)
Number of different *egl* *	N/A	22	N/A

RIF and ITS marker characteristics for a set of 818 strains from the PBC and ICMP collections. Sequence contigs are assembled from read pairs.

a -Total strains include characterized strains (separated from the total in parentheses) and unknown accessions.

b - One strain did not produce a RIF amplicon that was visible on an agarose gel but did produce sequence data.

c - the 191 strains only include those that produced an *egl* amplicon (Supplemental Table S2). RIF was sequenced in both directions more often (183/191 = 95.8%) than the *egl* gene (159/191 = 83.2%) for the same strains (Supplemental Table S2).

d - Of the 32 failed sequencing reactions with *egl* 19 had a single direction read.

*- Computed using a single representative for each unique sequence.

#### *Xanthomonas* species

Over 87.3% of the 361 *Xanthomonas* strains from the PBC yielded usable RIF sequence. The success rate for the ICMP strains was lower due to problems associated with shipping the DNA. In total, RIF sequences were obtained for 315 PBC and 43 ICMP strains (Table 5) representing 20 species and 29 pathovars (RIF sequence in Supplemental Table S2).

**Figure 3:**
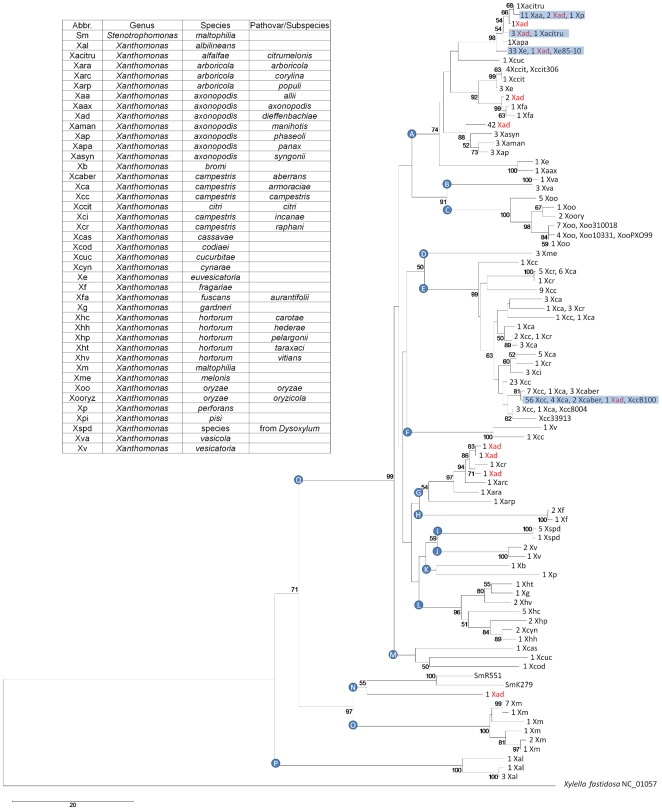
Classifying *Xanthomonas* based on RIF genotypes. The rooted neighbor-joining cladogram was constructed from 358 characterized *Xanthomonas* strains from the PBC (Table 2), ten reference strains from GenBank (see Table 1 for strain names) and the outgroup *Xylella fastidiosa*. *Xanthomonas* abbreviations are given in the text box. Identical sequences are represented only once and the number of sequenced strains is indicated on each leaf. Bootstrap values of >50% (shown at the node) are expressed as a percentage of 5,000 replicates. Blue boxes contain strains of different species with identical RIF sequences. *X. axonopodis* pv. *dieffenbachiae* strains are found throughout the tree (red text).

After automated alignment and trimming to 558 nt (see Methods), 82 different RIF sequences were obtained for *Xanthomonas* (Figure 3). These sequences formed 10 major clades in a neighbor-joining cladogram, each of which contained a dominant species: *X. vasicola* (B); *X. oryzae* (C); *X. melonis* (D); *X. campestris* (E); *X. arboricola* (G); *X. fragariae* (H); *X.* sp. from *Dysoxylum* (I); *X. vesicatoria* (J); *X.* (*Stenotrophomonas*) *maltophilia* (N and O) and *X. albilineans* (P). The species *X. alfalfae*, *X. euvesicatoria*, *X. perforans*, *X. citri* and *X. fuscans*, previously all classified as pathovars of *X. axonopodis* [13], grouped with the majority of *X. axonopodis* strains in the polyphyletic clade A. RIF sequences of *X. axonopodis* pv. *dieffenbachiae* were scattered throughout the RIF tree in clades A, E, G and N (red text in Figure 3). Clades F, G, K, M and N contained more than one species, but no two species shared the same RIF sequence. However, four RIF sequences in clades A and E (blue boxes in Figure 3) were shared by two or more species. Two *X. campestris* strains (clades F and G in Figure 3) were placed outside the main *X. campestris* clade E. Strains of the rice pathogen Xoo [3] yielded 5 different RIF sequences, all of which differed from the closely related *X. oryzae* pv. *oryzicola* by at least four nucleotides (Figure 3).

#### *Ralstonia solanacearum*

**Figure 4:**
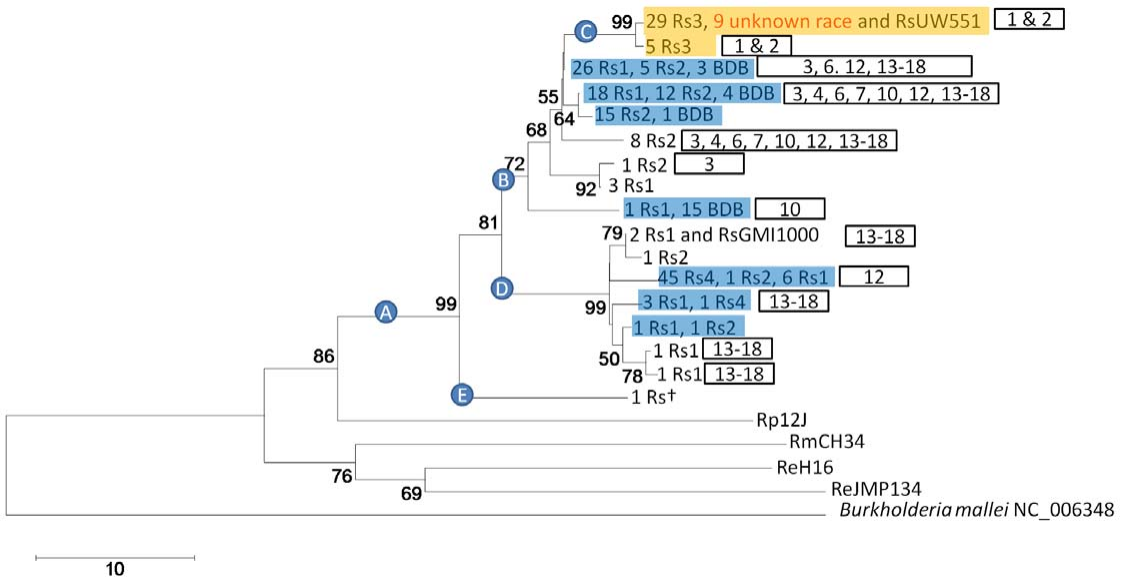
*Ralstonia solanacearum* strains classified based on RIF genotypes. The rooted neighbor-joining cladogram was constructed from 210 characterized *Ralstonia* strains from the PBC (Table 2), six reference strains from GenBank (see Table 1 for strain names)and the outgroup *Burkholderia mallei*. Identical sequences are represented only once and the number of sequenced strains is indicated on each leaf. Bootstrap values >50% (shown at the node) are expressed as a percentage of 5,000 replicates. All Rs strains (clade A) are clearly separated from other *Ralstonia* species. Rs1, Rs2, Rs3 and Rs4 are the four races of *R. solanacearum*, BDB is the blood disease bacterium and Rs† is *R. solanacearum* UW433 (ACH0732) which is an atypical race 1 biovar 2 strain [19]. Blue boxes contain strains of Rs races 1, 2, 4 and BDB that share an identical RIF sequence. In contrast, orange boxes contain only Rs3 strains showing that these RIF sequences are specific to race 3. Numbers in boxes next to tree leaves are *egl* sequevars obtained for those strains. Red text indicates Rs strains with no previous race designation.

The RIF marker was successfully sequenced for 210 (87.8%) of 239 characterized strains of *R. solanacearum* representing four races, blood disease bacterium (BDB), and the atypical strain *R. solanacearum* UW433 (ACH0732). Seventeen different sequences were obtained after the alignment was trimmed to 617 nucleotides (Figure 4). In order to compare the sequencing efficiency and resolution of RIF to that of the *egl* marker, which has been used to classify Rs into sequevars [10], we attempted to generate *egl* sequence from 191 characterized Rs strains (Table 5). RIF was sequenced with a 12.6% higher success rate than *egl*. Strains for which both RIF sequence and an *egl* sequevar had been obtained were compared in a neighbor-joining tree (Figure 4). The *egl* sequevars did not group into a single clade in the RIF tree, e.g. *egl* sequevars 13–18 are present in both clades B and D. The different groupings of strains with the RIF and *egl* markers are in agreement with Fegan's observations for Rs strain ACH0732 grouping differently with the ITS, *egl* and polygalacturonase markers [18], and may again be indicative of HGT. Thus, although greater resolution was achieved with the *egl* marker (22 different sequences) than with the RIF marker (17 different sequences), the RIF marker, which is under stabilizing selection [41], should more accurately portray genetic relationships than the positively selected pathogenicity related *egl* gene.

#### *Clavibacter michiganensis* subspecies

**Figure 5:**
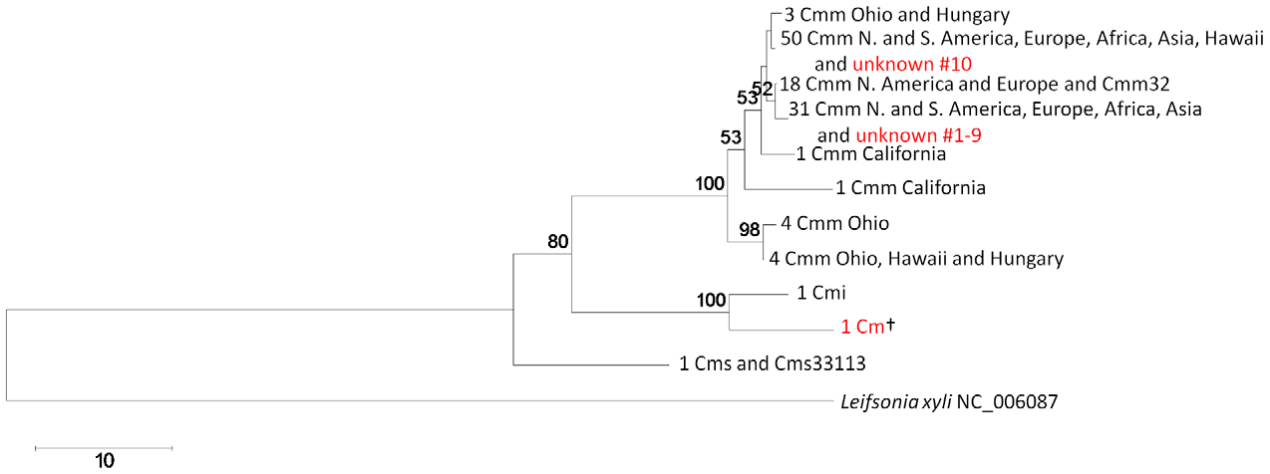
The RIF marker separates *Clavibacter michiganensis* strains into the three subspecies. The rooted neighbor-joining cladogram was constructed from 114 characterized *Clavibacter* strains from the PBC (Table 2), two reference strains from GenBank (see Table 1 for strain names) and the outgroup *Leifsonia xyli*. Identical sequences are represented only once and the number of sequenced strains is indicated on each leaf. Bootstrap values >50% (shown at the node) are expressed as a percentage of 5,000 replicates. Red text indicates *C. michiganensis* strains with no previous subspecies designation. Cm† is a non-pathogenic strain isolated from tomato that is most similar to Cmi. Unknowns #1-10 were isolated from a recent bacterial canker outbreak of tomato (see text) and perfectly match two of the Cmm RIF reference sequences. Cmm: *C. michiganensis* subsp. *michiganensis*. Cmi: *C. michiganensis* subsp. *insidiosus*. Cms: *C. michiganensis* subsp. *sepedonicus*.

RIF sequences were generated for 114 of 115 *Clavibacter michiganensis* strains (99% success rate) including 111 strains belonging to *C. michiganensis* subsp. *michiganensis* that had been collected from every continent except Australia and Antarctica (Figure 5 and Table 5). Ten different sequences remained after the alignment was trimmed to 660 nt. The resulting neighbor joining tree contains three clades representing the three subspecies. Notably, there was no correlation between the RIF sequence of Cmm strains and their geographic origin, i.e. Cmm strains from distant geographic locations shared the same RIF sequence. Worldwide distribution of this seed-borne pathogen on infected tomato seed may account for this lack of correlation.

### Expansion of RIF to three genera of the Enterobacteriaceae

To further evaluate the utility of RIF, we analyzed the plant pathogenic bacteria *Dickeya*, *Pectobacterium* and *Pantoea*. Initial Enterobacteriaceae RIF primer sets developed from sequenced strains of *Dickeya* (Dd_3937), *Pectobacterium* (Pa_1043) and *Erwinia* (NC_013971.1) were used for PCR amplification and sequencing (Table 3). New primers designed from the more recently sequenced *Dickeya* (Dd_703 and Dd_586) and *Pectobacterium* (Pcc_PC1 and Pw_Wpp163) genomes have improved amplification rates and genus specificity (data not shown).

**Figure 6:**
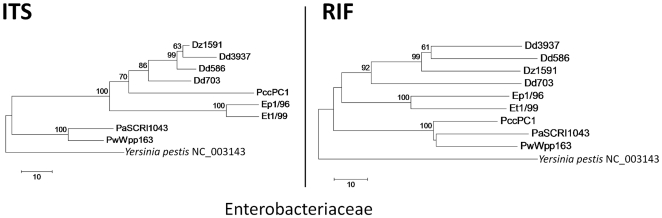
Neighbor-joining cladograms for the family Enterobacteriaceae using ITS and RIF sequences. Sequences for *Dickeya*, *Pectobacterium*, *Pantoea* and *Erwinia* were extracted from fully sequenced strains in GenBank (Table 1). Bootstrap values >50% (shown at the node) are expressed as a percentage of 5,000 replicates. Note the longer branch lengths for the *Erwinia* and *Dickeya* species in the RIF tree.

*In silico* analysis of the RIF and ITS markers of the nine completely sequenced *Dickeya*, *Pectobacterium* and *Erwinia* genomes revealed 2–3 times more polymorphic nucleotides in RIF than ITS with *Dickeya* (80 vs. 40 nt) and *Erwinia* (58 vs. 21 nt) (Figure 6). These distances are similar to those observed for different species of *Xanthomonas* and illustrate the higher resolution of the RIF marker. In contrast, *Pectobacterium* strain PccPC1 was separated from other *Pectobacterium* accessions by longer branches in the ITS than the RIF tree (Figure 6). This is due to the numerous nucleotides affected by several indels in the ITS sequence of PccPC1 (27 nucleotides from multiple insertions and six nucleotides from a deletion relative to the two other *Pectobacterium* strains).

The efficacy of the RIF and ITS markers was directly compared for 96 Enterobacteriaceae accessions (Table 6). RIF was easier to amplify and sequence in this family than ITS as illustrated by the higher success rate for the direct sequencing of RIF (53) as compared to ITS (35) from 64 strains of *Dickeya* (all verified to be *Dickeya* with the ADE primers [51]). A greater number of different sequences was obtained with RIF (12) than with ITS (11) for the same set of *Dickeya* strains (Supplemental Figure S5). We were able to obtain RIF but no ITS sequence for fourteen *Pectobacterium* strains. The difficulty in sequencing ITS was likely due to the presence of multiple rDNA copies that differ in sequence and length in these Enterobacteriaceae genomes. All *Pantoea* strains and the *Pectobacterium* strains K0509, K0574 and K0522 required a lower stringency annealing temperature (51°C vs 61°C) for RIF amplification (Table 6 and Supplemental Table S2).

**Table 6:** Comparison of ITS and RIF markers for three genera of Enterobacteriaceae.

	*Dickeya*	*Pectobacterium* a	*Pantoea* a
Total Strains (characterized PBC reference strains)	64 (9)	23 (19)	9 (9)
ITS amplicon	59	10	8
ITS sequence	35	0	N/A^b^
RIF amplicon (characterized PBC reference strains)	60 (7)	18 (8)^c^	6(2)^d^
RIF sequence (characterized PBC reference strains)	53 (7)	15 (11)	6 (6)
RIF amplicon, no ITS amplicon	1	5	0
RIF sequence, no ITS sequence	25	N/A	N/A
ITS amplicon, no RIF amplicon	1	0	4^e^
ITS sequence, no RIF sequence	6	N/A	N/A
ITS amplicon, no ITS sequence	24	N/A	N/A
No ITS contig but ITS read	23	N/A	N/A
RIF amplicon, no RIF sequence	6	1	0
No RIF contig, but RIF read	6	0	0
Size of ITS sequence (nt)	416–425	N/A	N/A
Size of RIF sequence (nt)	722	722	722
Number of strains with both the ITS and RIF sequenced	29	N/A	N/A
ITS average nucleotide differences*	10.5	N/A	N/A
RIF average nucleotide differences*	40.3	55.8	44
Number of different ITS sequences*	11	N/A	N/A
Number of different RIF sequences			
(characterized PBC reference strains)*	17 (6)	10 (8)	2 (2)

RIF and ITS marker characteristics for a set of 96 strains from the PBC collection. Sequence contigs are assembled from read pairs.

*- Computed using a single representative for each unique sequence.

a - Eight *Pectobacterium* strains and one *Pantoea* strain failed to yield either ITS or RIF amplicons after multiple attempts to produce an amplicon from reisolated DNA (Supplemental Table S2).

b - *Pantoea* strains were not sequenced with ITS.

c - 15 strains were amplified at 61°C and 3 strains at 51°C.

d - 2 strains were amplified at 61°C and 4 strains at 51°C.

e - 2 strains were amplified at 61°C and 2 strains at 51°C.

**Figure 7:**
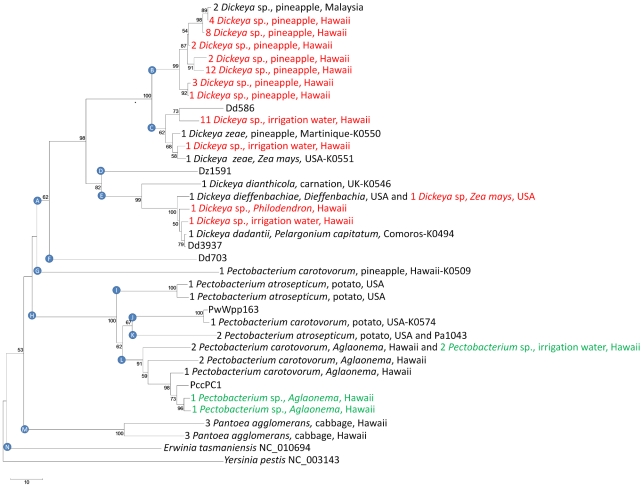
RIF tree of plant pathogenic Enterobacteriaceae. The rooted neighbor-joining cladogram was constructed from 74 Enterobacteriaceae strains, including 24 characterized strains of *Dickeya*, *Pectobacterium* and *Pantoea* from the PBC (Supplemental Table S2), eight reference strains from GenBank (see Table 1 for strain names) and the outgroup *Yersinia pestis*. Identical sequences are represented only once and the number of sequenced strains is indicated on each leaf. Bootstrap values >50% (shown at the node) are expressed as a percentage of 5,000 replicates. The true *Erwinia* species *E. tasmaniensis* separates from the renamed *Erwinia* species that branch into three different genera: *Dickeya* (clade A), *Pectobacterium* (clades G and H) and *Pantoea* (clade M). Red text indicates uncharacterized strains of *Dickeya* and green text indicates uncharacterized strains of *Pectobacterium*. K numbers are shown for the accessions discussed in the text (Supplemental Table S2).

Twenty-nine different RIF sequences were obtained for Enterobacteriaceae strains after the alignment was trimmed to 722 nt (Table 6 and Figure 7). *Dickeya*, *Pectobacterium*, *Pantoea*, *Erwinia* and *Yersinia* formed distinct clades in the neighbor joining tree (Figure 7). Notably, four *D. dadantii* strains were located in three separate sub-clades within the *Dickeya* clade (Figure 7). The sequenced strain *P. wasabiae* Wpp163 (formerly named *P. carotovorum*) and the potato strain *P. carotovorum* K0574 (classified based on bacteriological tests) differed from each other by only three nt. Similarly, the distance between several different *P. atrosepticum* strains in clades I and K (Figure 7) was greater (54.5 nt) than that between the species *P. atrosepticum* and *P. carotovorum* (50.8 nt and 48.6 nt, respectively, in clades J and K).

### Classifying uncharacterized strains based on RIF genotypes

Sequence frameworks were used to rapidly classify 70 uncharacterized cultures (11 Cm, 4 *Pectobacterium*, 46 *Dickeya* and 9 Rs). Ten putative Cmm strains from multiple outbreaks of tomato canker were classified within 48 hours of their arrival and matched two different cosmopolitan Cmm reference genotypes (Figure 5). A single Cm strain non-pathogenic on tomato (Cm† in Figure 5) from the PBC had a RIF genotype very similar to that of the *C. michiganensis* subsp. *insidiosus* reference strain.

Fifty strains of *Pectobacterium* and *Dickeya* from the PBC that could not be identified to species based on biochemical tests were placed in the RIF framework. Two strains of *Pectobacterium* sp. isolated from *Aglaonema* in Hawaii and two strains isolated from water grouped with the *P. carotovorum* clade near RIF genotypes from known reference strains (green text in Figure 7). The other forty-six strains grouped with distinct *Dickeya* reference strains (red text in Figure 7).

Nine Rs strains isolated from Guatemalan geraniums, and verified to be Rs by bacteriological and immunodiagnostic tests, grouped with Rs race 3 biovar 2 strains in clade C of the RIF tree (red text in Figure 4), which contains the select agent Rs_UW551. Thus, the RIF frameworks, even in their current form, have proven useful for classifying RIF sequences from uncharacterized isolates of four genera.

### *In silico* classification of *Xylella* and *Pseudomonas* with RIF

The potential of RIF to classify bacteria from additional plant associated genera was further examined with an *in silico* analysis of *Pseudomonas* and *Xylella* strains available in GenBank. All but one of the completely sequenced strains contained a single copy of the *dnaA* gene and more than one copy of the ITS region (*P. aeruginosa* strain 39016 contains a single copy of both ITS and RIF). RIF primers for *X. fastidiosa* were developed from five strains (Table 3), primers for *Pseudomonas* were developed from twenty-three strains representing eight species. The five *Xylella* strains were classified into three RIF genotypes, whereas every *Pseudomonas* strain had a different RIF sequence.

### Unexpected nucleotide distances between genera, species and subspecies using RIF

The reference frameworks constructed from 125 different RIF sequences (82 *Xanthomonas*, 17 Rs, 10 Cm, 8 *Pectobacterium*, 6 *Dickeya* and 2 *Pantoea*) allowed comparisons of the distances between genera, species and subspecies. In general, the distances (Supplemental Tables S3, S4, S5, S6, S7) as measured in nucleotide substitutions were greatest between genera and smallest between subspecies as would be expected. Exceptions included: (i) the genera *Stenotrophomonas* and *Xanthomonas* were separated by fewer nucleotide differences (70.5 nt) than species of *Dickeya* (90 nt) (Supplemental Tables S3 and S6); (ii) two species, *X. pisi* and *X. bromi* (asterisk in Supplemental Table S3), were separated by only 28 nucleotide differences (clade K in Figure 3) whereas subspecies of *Clavibacter michiganensis* were separated by 31 nucleotide differences; (iii) the strains within subclades of *Stenotrophomonas maltophilia* (clades N and O in Figure 3) differed by 50.1 nucleotides, whereas species of *Ralstonia* and species of *Xanthomonas* were separated by only 56 and 49.5 nucleotides, respectively. The inconsistent distances at different taxonomic levels may be due to the incorrect grouping of diverse strains to one taxon or the misclassification of some strains in the collection based on initial genetic and phenotypic analysis. Some of these inconsistencies may identify potential problems with the classification, identification or naming of these organisms.

## Discussion

### DnaA structure in relation to the RIF marker region

The DnaA protein is an essential core metabolic protein in bacteria known to regulate the initiation of chromosomal replication by binding DnaA boxes on the replicating chromosome [52]. The absence of the *dnaA* gene from eleven sequenced genomes of insect endosymbionts has been suggested to reflect the extreme dependency that these symbionts have with their host [53]. The DnaA protein contains several conserved domains, including a AAA+ superfamily domain near the N-terminal and a helix-turn-helix DNA binding domain at the C-terminal of the protein [52]. The primers were designed near, but not in, the AAA+ domain and on the edge of the helix-turn-helix DNA binding domain (Supplemental Figure S1). Thus, these conserved regions may function at the nucleotide rather than protein level, possibly in gene transcription or mRNA stability.

### RIF can be used as an identification marker for the majority of bacteria

The fact that *dnaA* is a single copy gene in the vast majority of complete bacterial genomes examined and does not appear to frequently undergo horizontal gene transfer make it a valuable marker for bacterial strain classification. The ease with which we were able to produce sequence frameworks for six genera illustrates the practicability of this marker, even though RIF amplification required different primers for each genus. Since previous efforts to identify universal primers that amplify protein-coding regions were unsuccessful ([43] and Supplemental Text S1), the need for genus-specific primers was expected.

### RIF can add resolution to multi-locus sequence analysis

The RIF framework for *Xanthomonas* was similar in structure to the tree produced for a *Xanthomonas* MLSA by Young et al. [25]. Although the larger amount of sequence data used in multi-locus analyses is expected to provide more resolution than any single marker, in this particular example five SNPs distinguished the RIF markers of Xcc strains 8004 and 33913 (Figure 1) while the four markers used in the MLSA were identical in the two strains (Supplemental Table S8). This suggests that RIF not only performs well as a single marker, but also that it should be considered for inclusion in multi-locus studies, such as those incorporated into PAMdb [54], an online MLSA database for classification of *Xanthomonas*, *Pseudomonas*, *Ralstonia* and *Acidovorax*. Indeed, a partial sequence of the *dnaA* gene (covering 52.5% of RIF) was included as one of eight loci in a recent MLSA of *Rickettsia* [55].

### RIF provided greater resolution, higher sequencing success rate and more reliable strain groupings than ITS

Reference frameworks constructed with RIF can be used to classify strains of *Clavibacter*, *Dickeya*, *Pantoea*, *Pectobacterium*, *Ralstonia*, and *Xanthomonas*. Although RIF was originally derived by comparative genomics of *Xanthomonas*, it turned out to have better resolution than ITS for all genera except *Clavibacter*. The increased resolution may be attributed to the longer amplicon produced by RIF (654–700 nt) versus ITS (504–590 nt). In addition to providing higher resolution than ITS, RIF also had a higher sequencing success rate using the direct amplicon sequencing method. Also, RIF is part of a protein coding gene, which is more resistant to compensatory mutations, indels and inversions, thus yielding more consistent phylogenetic trees than those obtained with ribosomal DNA. For *Ralstonia*, RIF had a higher sequencing success rate than *egl*, but *egl* provided a greater number of unique sequences. However, *egl* is a pathogenicity related gene that may be prone to horizontal transfer.

### RIF classification is consistent with other methods of classification

The cladograms constructed using the RIF marker were consistent with existing trees based on single or multiple markers (where available). RIF sequences supported previous genetic studies of *Xanthomonas*, the close relationship between *Stenotrophomonas* and *Xanthomonas* and the reclassification of *Erwinia chrysanthemi*, *E. carotovora* and *E. herbicola* to *Dickeya*, *Pectobacterium* and *Pantoea*, respectively [9]–[11], [13], [22], [23], [25]–[27].

RIF genotypes did not correspond to the current nomenclature for pathovars of *X. campestris* and *X. axonopodis*, nor to the race designations of *R. solanacearum* as expected based on previous observations [4], [5], [9], [11].

The polyphyletic clade A of *Xanthomonas* (Figure 3) contained six species, five of which have recently been renamed from *X. axonopodis* to *X. alfalfae*, *X. euvesicatoria*, *X. perforans*, *X. citri* and *X. fuscans* [15], [16]. These six species fell into subclades that agree with the groups of *X. axonopodis* observed by Vauterin et al. [13].

Classification of unknown *Clavibacter* strains isolated from a recent outbreak of bacterial canker based on RIF corresponded to the groupings obtained with rep-PCR fingerprinting analysis (unpublished): nine strains were identical while the tenth fell into a separate group with both methods.

### The RIF database

In order to enable comparison of strains isolated worldwide with those characterized in this study, we have created an online database, RIFdb. For ease of use, the database can be queried with unprocessed single or paired chromatograms, or FASTA sequences, to search for the best reference strain match. Chromatograms are base-called, and paired chromatograms are assembled by RIFdb (see methods) prior to the search. Querying the database with a single read reduces the cost of identification if a user decides to sequence only one end. Queries are automatically aligned with existing sequences and visualized in a neighbor-joining tree using ArchaeopteryxE [56]. The RIF sequences in the database are automatically re-trimmed if a partial sequence is provided as a query, although excessive trimming will decrease the resolution of this marker.

Clearly the utility of the RIF database will increase as more sequences from as yet unrepresented accessions from around the world are added. Sequences of strains from diverse bacterial collections will increase global representation. Deposition of high quality chromatogram pairs along with key characteristics of strains will enable the expansion of RIFdb with strains from international collections and increase its utility to diagnosticians worldwide.

### Conclusion

The RIF marker is suitable for the classification and meaningful grouping of strains from the six genera examined in this study. The RIF marker provides a greater sequencing success rate than the ITS in all six genera and a greater number of sequence barcodes than the ITS in three of four genera examined. Further expansion of RIF sequence frameworks with strains from other collections via the web accessible database will facilitate the classification of unknowns in the future. Finally, we show that the RIF marker system should be expandable to most bacterial genera, including *Xylella* and *Pseudomonas*.

## Methods

### Computational identification of suitable marker regions

The computational identification of a DNA barcode region that distinguishes closely related *Xanthomonas* strains was performed with completely sequenced *Xanthomonas* genomes (Table 1) as follows. First, SNPs were identified between the two *Xanthomonas oryzae* pv. *oryzae* genomes Xoo_10331 and Xoo_311018 using MUMmer [57] to match all conserved regions of 20 nucleotides or greater. A similar analysis was performed for two strains of *X. campestris* pv. *campestris* (Xcc_33913 and Xcc_8004). Nucleotides of Xoo_311018 and Xcc_33913 that had no MUMmer matches in the other strain of the same pathovar were masked with a Perl script. Second, single copy oligos in Xoo_311018 that were perfectly conserved in the five other *Xanthomonas* genomes (Xoo_10331, Xcc_33913, Xcc_8004, Xccit_306 and Xe_85-10) were identified using MUMmer [57] (-mum -l 20 -b) in sequential comparisons, and will be referred to as conserved 20^+^-mers. In this analysis, the regions conserved between Xoo_311018 and genome two were used as a query with genome three and so on. Third, a Perl script was used to identify regions in all six *Xanthomonas* genomes that a) were flanked by conserved 20^+^-mers, b) separated by >550 nucleotides, c) produced an amplicon of <1,000 nucleotides, and d) contained at least 20 masked out nucleotides (i.e. containing 2 or more SNPs) in Xoo_311018. The same analysis was performed using the masked genome of Xcc_33913. Fourth, another Perl script was used to align the extracted regions from the six *Xanthomonas* genomes using ClustalW [58] and output a distance matrix of nucleotide differences to confirm that orthologous regions from any two *Xanthomonas* strains contained one or more SNPs. Finally, a Perl script was used to extract every gene from the identified regions in Xcc_33913 and Xoo_311018, and confirm the presence of the gene in nine completely sequenced genomes of *Ralstonia*, *Clavibacter*, *Pectobacterium*, *Erwinia* and *Dickeya* (asterisk in Table 1) with a default BLAST [59] comparison. (No sequenced strains of *Pantoea* were available at the time of this study). The BLAST results were checked by hand to confirm the presence of the complete gene in all genomes of interest (asterisk in Table 1); only one gene (*dnaA*) was present in full in all genomes.

### Copy number determination of bacterial *dnaA* genes

A set of 3,020 bacterial DnaA protein sequences was downloaded by querying the NCBI protein database (limited to bacterial organisms) with “dnaA” and “350∶1000” limited to gene and SLEN, respectively. Of these, only 1,188 protein sequences whose headers contained the terms “dnaA”, “chromosomal replication initiator” or “chromosomal replication initiation” were kept. Separately, all 1,159 completely sequenced bacterial and archael genomes were downloaded from NCBI (ftp://ftp.ncbi.nih.gov/genbank/genomes/bacteria) using a Perl script. The directory containing the genome “*Escherichia coli* strain RS218” was left out as it improperly contained Enterobacteria phage CUS-3. The subset of 1,067 bacterial genomes on the “List of prokaryotic names with standing in nomenclature” (http://www.bacterio.cict.fr) and the list of cyanobacterial genera (http://www.cyanodb.cz/valid_genera) were used in this analysis. Subsequently, a protein set was generated for each of the 1,067 bacterial genomes. Twenty-one proteins from these sequenced genomes that were annotated as “dnaA”, “chromosomal replication initiator” or “chromosomal replication initiation” but whose accession number was not in the DnaA protein set were added to the protein set for a total of 1,209 sequences. Another Perl script was used to identify copies of the DnaA protein in the genome of each strain using BLAST (-p tblastn -m8 –X 300 –e 1e-10) and the 1,209 DnaA proteins as a query. Any gene in the 1,067 bacterial genomes with a match to any protein query at >40% identity over >350 amino acids was counted as a DnaA protein. That query was then used to identify additional copies of DnaA in the genome. The *dnaA* gene of *Yersinia pestis* strain D106004 could not be identified in this way because of a frame-shift mutation (Supplemental Table S1), so bl2seq analysis using the *dnaA* gene from *Y. pestis* Angola was used. The *dnaA* gene of *Lysinibacillus sphaericus* strain C3 41a began at position 4,639,741 of 4,639,821 nt of the circular chromosome instead of position 1. Genomes containing more than one copy of the *dnaA* gene were compared with themselves using bl2seq to identify possible gene duplication or horizontal transfer events.

### RIF primer design on the *dnaA* gene

The *dnaA* gene from the six sequenced *Xanthomonas* strains were first used for primer development. All sequences from *Xanthomonas* were aligned with ClustalW [58] and 18–20 nt primers were designed manually on conserved regions to produce the largest amplicon covering the greatest number of SNPs, shorter than 1,000 nucleotides. The primers were required to have a melting temperature of 60±2°C, 50–60% G+C content, end in a G or C and have no complementary bases at the ends. Only one of many possible regions covering all SNPs between the two Xcc and two Xoo strains was chosen. The gene previously extracted from *Clavibacter*, *Dickeya*, *Pectobacterium*, and *Ralstonia* was used to develop four more *dnaA* primer sets (Table 3). Primers for each genus had to be located within 60 nucleotides of the *Xanthomonas* primers and have a melting temperature of 60±2°C and 50–60% G+C content (Supplemental Figure S1). Primers were designed individually for *Ralstonia*, *Clavibacter*, *Pectobacterium* and *Dickeya*. *Pectobacterium* primers were used for *Pantoea* as no sequenced strains existed at the time (see DNA marker amplification below). Recently sequenced genomes allowed the development of primers with increased specificity to *Pectobacterium* and *Dickeya* using the *dnaA* gene from three *Pectobacterium* (Pa_1043, Pw_Wpp163 and Pcc_PC1) and three *Dickeya* strains (Dd_3937, Dz_1591 and Dd_703) (Table 3).

### Bacterial culturing and DNA extraction

A subset of 840 bacterial clones from the PBC was chosen for DNA sequencing (Supplemental Table S2). Fourteen of the clones chosen were from duplicate characterized strains and were not counted in the total number of sequenced strains. The strains were plated on TZC medium to confirm that no contamination was present and transferred into sterile deionized water for genomic DNA isolation and to LB containing 15–20% glycerol for long-term storage at −80°C. DNA was isolated by adding 300 µl of water culture to 100 µl of a 40% mixture of deionized water-Chelex-100® resin (BioRad), and incubated at 60°C for 60 minutes [60]. DNA from ten suspected *Clavibacter* strains from a tomato canker was isolated using a mixture of water-Chelex-100® resin (BioRad). DNA from 84 *Xanthomonas* strains extracted with the REDExtract-N-Amp™ PCR ReadyMix™ (Sigma-Aldrich) were provided by John Young and Duck-Chul Park from the ICMP in New Zealand.

### DNA marker amplification and sequencing

All markers were PCR amplified using an Eppendorf Mastercycler® Ep Gradient machine. All PCR reactions contained: 25 µl of JumpStart™ REDTaq® ReadyMix™ (Sigma-Aldrich) for high throughput PCR with 5 µl of 10 mM primer, 5 µl of template and 15 µl of deionized water. The cycling conditions included an initial denaturation step at 94°C for ten minutes, followed by 35 cycles of 94°C for 30 seconds, 61°C for one minute and 72°C for 30 seconds, followed by a ten minute extension at 72°C and a hold at 4°C. *Pectobacterium* and *Pantoea* strains that did not amplify with these settings were amplified with a 51°C annealing temperature using the *Pectobacterium* primers (RIF *Pectobacterium* amplification at 51°C in Supplemental Table S2). ITS marker amplifications for *Xanthomonas*, *Clavibacter*, *Ralstonia*, *Dickeya*, *Pectobacterium* and *Pantoea* were performed as described by Normand [20]. The presence of multiple rDNA operons of different sizes in the genus *Dickeya* necessitated manual isolation of the smaller ITS amplicon from an agarose gel using the band-stab method of Wilton [40], followed by 25 cycles of PCR. ADE marker amplification for *Dickeya* and *egl* marker amplification for *R. solanacearum* strains were performed as described by Nassar [51] and Fegan [19], respectively. All PCR products were separated on a 1.5% agarose gel to confirm quantity and quality, and DNA was purified and sequenced from all PCR attempts, even if no PCR product was visible on the gel. PCR products were purified using a Qiaquick 96 PCR Purification Kit (Qiagen), and the product was sequenced at the University of Hawaii sequencing facilities using forward and reverse primers.

### DNA marker analysis

Chromatograms of sequenced amplicons were automatically converted to FASTA files using *phred* [49] with default settings and no trimming. Forward and reverse sequences were automatically assembled with *phrap* [50], set to a minimum match of 100 and a minimum quality score of 20. A Perl script was used to identify the longest stretch of high quality bases containing no more than five consecutive low quality bases from the ace file. Low quality bases were edited manually with BioEdit [61] and either retained, removed, or converted into degenerate bases. Assembled sequences spanning fewer than 550 high-quality bases and sequence reads that could not be assembled were removed. RIF sequences that did not match the expected strain annotation were re-isolated and re-sequenced. RIF sequences of each genus were aligned using MEGA [62] and trimmed to the same length. Distance matrices, and neighbor-joining (N-J) phylogenetic trees with bootstrapping scores, from 5000 replicates, were produced with MEGA using pairwise deletions and the number of nucleotide differences to indicate branch length. In and between group distances and average distances were computed with complete deletions using all RIF sequences from characterized strains in each respective clade of the genus-specific neighbor-joining tree. In and between group distances, average distances and N-J trees were produced using a single representative for each different sequence. Both the ITS and RIF markers were re-amplified and sequenced for strains that branched in unexpected positions to confirm their RIF sequence. N-J trees were rooted with the orthologous region obtained via BLAST [59] from a closely related sequenced strain in NCBI. Assembled high quality trimmed sequences for all accessions have been deposited in GenBank under accession numbers HM180945-HM181929 and HM469616-HM469894. The RIFdb database can be queried at http://genomics.hawaii.edu/RIFdb/.

## Supporting Information

Figure S1 **Multiple sequence alignment of nucleotides 311 to 1311 of the** ***dnaA*** **genes of six genera.** Primer regions are shown for *Clavibacter*, *Xanthomonas*, *Ralstonia*, *Erwinia*, *Dickeya* and *Pectobacterium*. Primer binding regions are shown in red with black background. The AAA+ domain (green) [52] and the C-terminal domain (pink) [52] are highlighted.
(TIF)

Figure S2 **RIF distinguishes more** ***Xanthomonas*** **strains than ITS.** Unrooted neighbor-joining trees for the RIF and ITS markers were constructed from eighty-four *Xanthomonas* strains from the PBC (Supplemental Table S2) and eight reference strains from GenBank (see Table 1 for strain names) . Identical sequences are represented only once and the number of sequenced strains is indicated on each leaf. Bootstrap values >50% (shown at the node) are expressed as a percentage of 5,000 replicates. Two *X. campestris* strains and one *X. axonopodis* strain localize to the appropriate clade with RIF but not ITS (red asterisk). Xc - *X. campestris*, Xa - *X. axonopodis*, Xe – *X. euvesicatoria*, Xcit – *X. citri*, Xo *- X. oryzae*, Xm – *X.* (*Stenotrophomonas*) *maltophilia*.
(TIF)

Figure S3 **RIF sequences distinguish more** ***Ralstonia*** **strains than ITS.** Unrooted neighbor-joining trees for the RIF and ITS markers were constructed from ninety-seven *Ralstonia* strains from the PBC (Supplemental Table S2) and three reference strains from GenBank (see Table 1 for strain names). Identical sequences are represented only once and the number of sequenced strains is indicated on each leaf. Bootstrap values >50% (shown on the node) are expressed as a percentage of 5,000 replicates. Rs strains grouped differently with the two markers, as illustrated by strains K0157, K0024, K0190 and K0018, which were re-sequenced and are marked with an asterisk. Although the average nucleotide difference between groups of Rs with the ITS marker is high, there is little sequence variation within each individual group (clades A, B and C), and fewer strains are resolved than with the RIF marker. Also, ITS sequence from *Ralstonia pickettii* strain 12J is placed within Rs clade B on the ITS tree, while RIF sequence from the same strain is placed outside Rs clade B on the RIF tree.
(TIF)

Figure S4 **RIF sequences distinguish fewer strains of** ***Clavibacter*** **but produce a more robust tree.** Unrooted neighbor-joining trees for the RIF and ITS markers were constructed from nineteen *Clavibacter* strains from the PBC (Supplemental Table S2) and two reference strains from GenBank (see Table 1 for strain names). Identical sequences are represented only once and the number of sequenced strains is indicated. Bootstrap values >50% (shown at the node) are expressed as a percentage of 5,000 replicates.
(TIF)

Figure S5 **RIF sequences distinguish more** ***Dickeya*** **strains than ITS.** Unrooted neighbor-joining trees for the RIF and ITS markers were constructed from twenty-nine *Dickeya* strains from the PBC (Supplemental Table S2) and three reference strains (with strain names) from GenBank (Supplemental Table S1). Identical sequences are represented only once and the number of sequenced strains is indicated. Bootstrap values >50% (shown at the node) are expressed as a percentage of 5,000 replicates.
(TIF)

Table S1 **Copy number and location of the** ***dnaA*** **genes in 1,067 sequenced NCBI strains.**
(PDF)

Table S2 **Strains used in this study.**
(PDF)

Table S3 ***X. albilineans*** **is more distantly related to other** ***Xanthomonas*** **species than is** ***Stenotrophomonas maltophilia*** **.**
(PDF)

Table S4 **Average between species distances of the RIF marker from twenty-three different** ***Ralstonia*** **RIF sequences.**
(PDF)

Table S5 **Average between group distances of the RIF marker from ten different** ***Pectobacterium*** **RIF sequences.**
(PDF)

Table S6 **Average between group distances of the RIF marker from ten different** ***Dickeya*** **RIF sequences.**
(PDF)

Table S7 **Average between subspecies distances of the RIF sequence from eleven different** ***Clavibacter michiganensis*** **RIF sequences.**
(PDF)

Table S8 ***In silico*** **comparison of** ***Xanthomonas*** **with the RIF marker resolves one pair of closely related strains that is unresolved with four other housekeeping genes and the ITS.**
(PDF)

Text S1 **Computational derivation of a universal DNA marker from fifteen completely sequenced genomes was unsuccessful.**
(DOC)
