# Supplementary figures and images for: Classification of Plant Associated Bacteria Using RIF, a Computationally Derived DNA Marker
Source: PLoS One. 2011 Apr 21;6(4):e18496. doi: 10.1371/journal.pone.0018496 (PMC3080875; doi:10.1371/journal.pone.0018496)

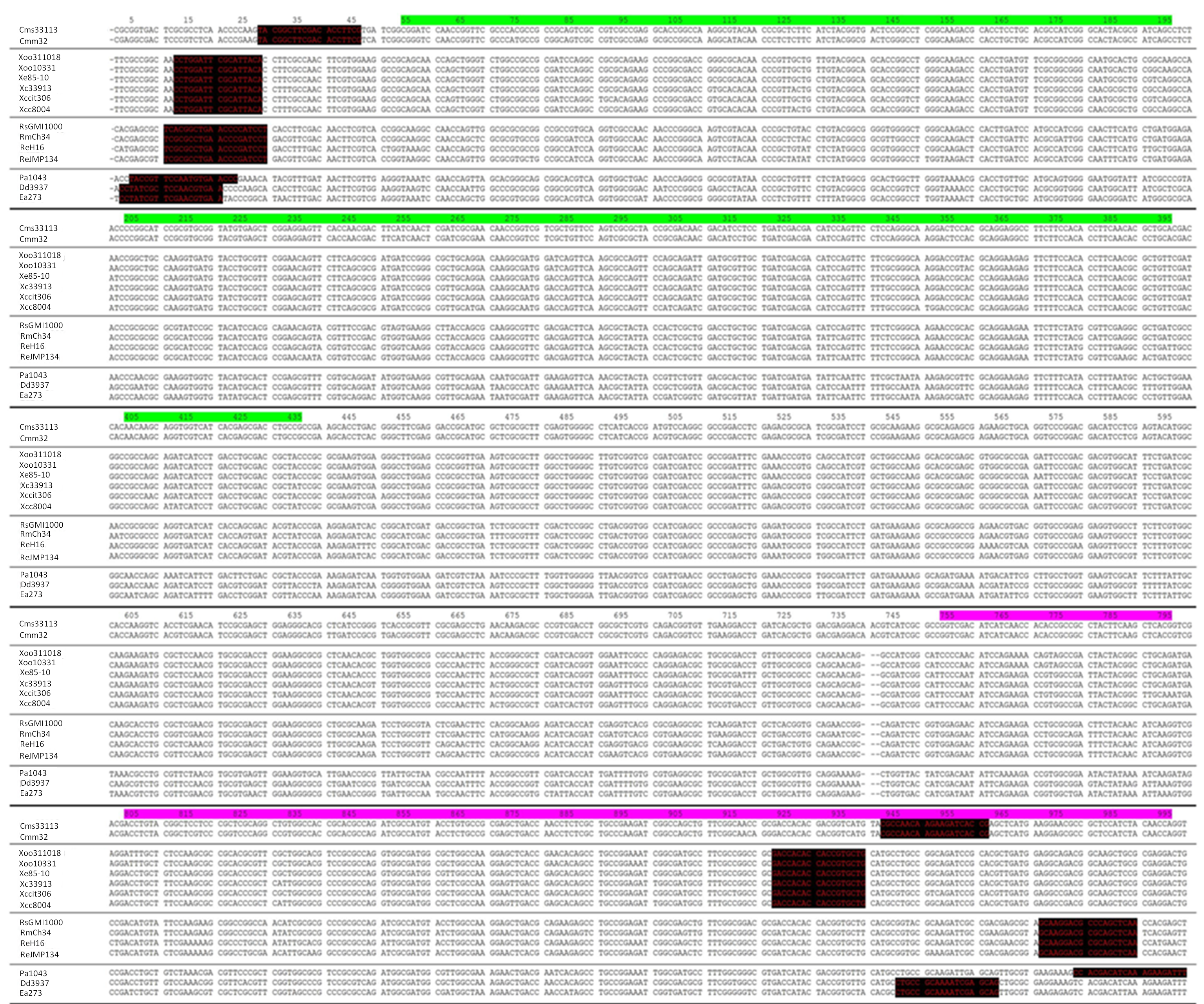

Supplement: Figure S1 — Multiple sequence alignment of nucleotides 311 to 1311 of the dnaA genes of six genera. Primer regions are shown for Clavibacter, Xanthomonas, Ralstonia, Erwinia, Dickeya and Pectobacterium. Primer binding regions are shown in red with black background. The AAA+ domain (green) [52] and the C-terminal domain (pink) [52] are highlighted. (TIF) [file pone.0018496.s001.tif]

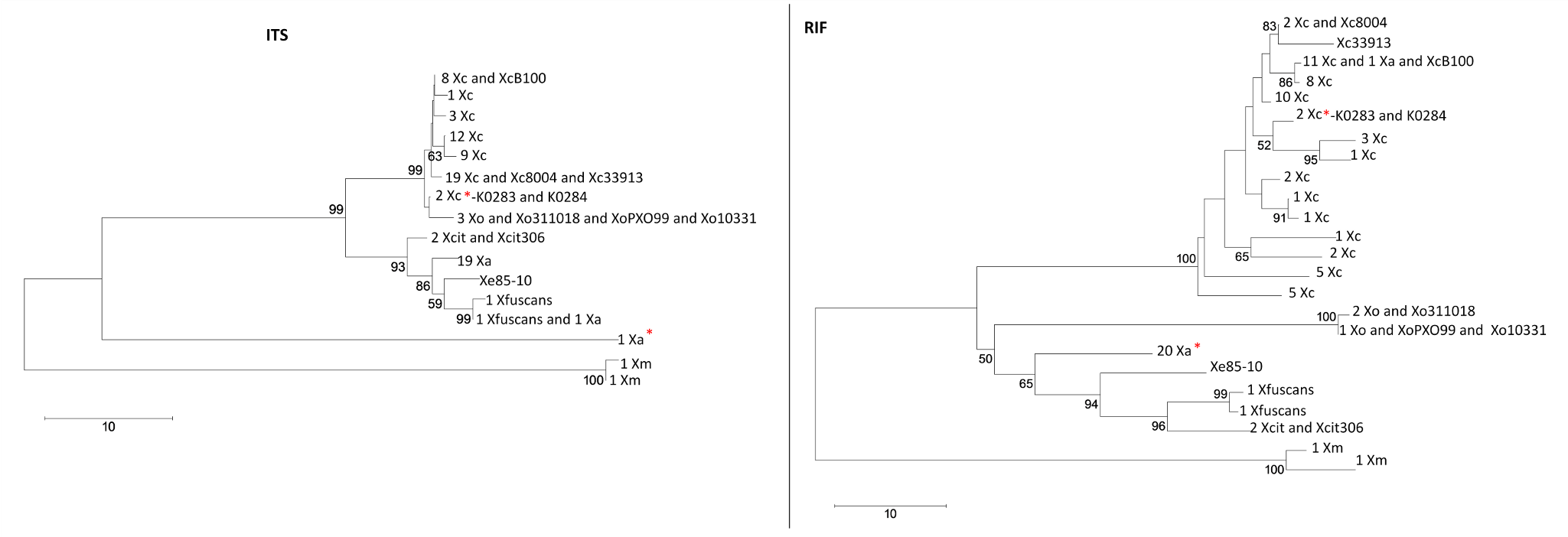

Supplement: Figure S2 — RIF distinguishes more Xanthomonas strains than ITS. Unrooted neighbor-joining trees for the RIF and ITS markers were constructed from eighty-four Xanthomonas strains from the PBC (Supplemental Table S2) and eight reference strains from GenBank (see Table 1 for strain names) . Identical sequences are represented only once and the number of sequenced strains is indicated on each leaf. Bootstrap values >50% (shown at the node) are expressed as a percentage of 5,000 replicates. Two X. campestris strains and one X. axonopodis strain localize to the appropriate clade with RIF but not ITS (red asterisk). Xc - X. campestris, Xa - X. axonopodis, Xe – X. euvesicatoria, Xcit – X. citri, Xo - X. oryzae, Xm – X. (Stenotrophomonas) maltophilia. (TIF) [file pone.0018496.s002.tif]

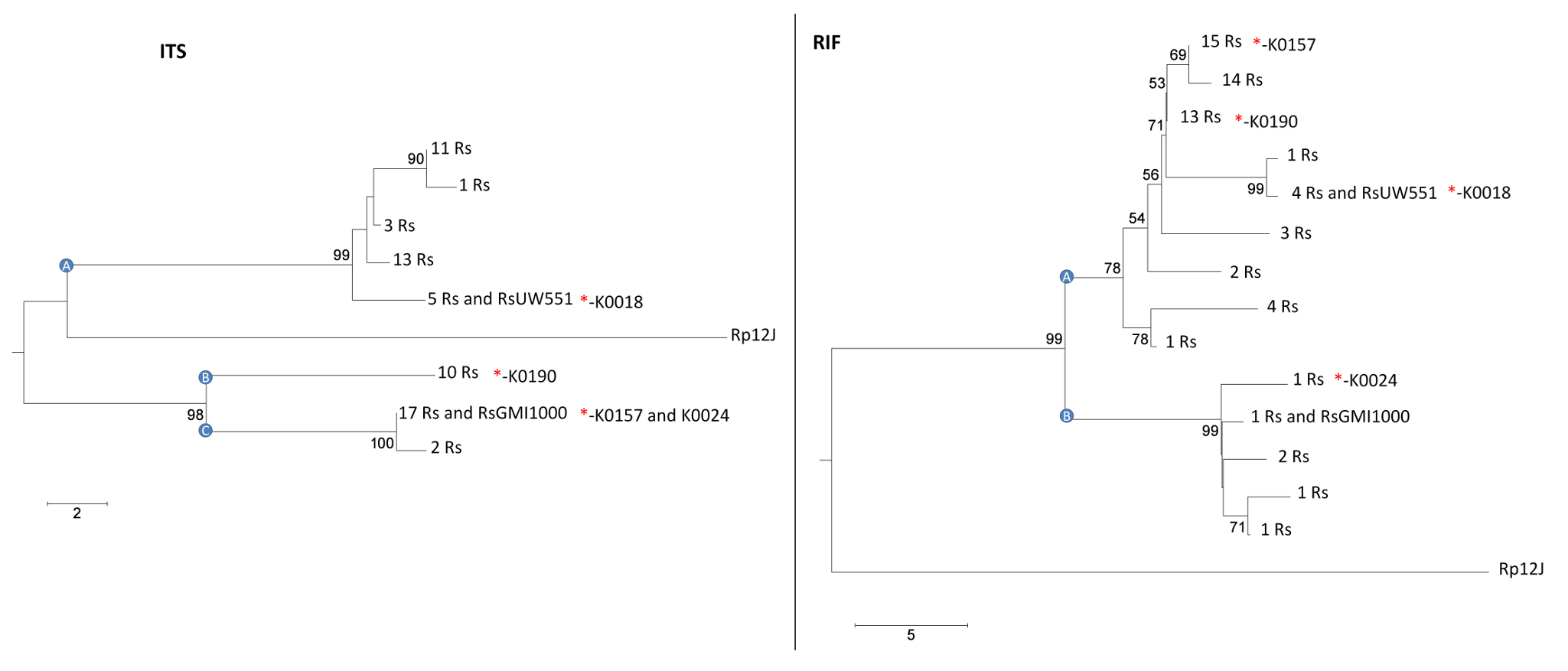

Supplement: Figure S3 — RIF sequences distinguish more Ralstonia strains than ITS. Unrooted neighbor-joining trees for the RIF and ITS markers were constructed from ninety-seven Ralstonia strains from the PBC (Supplemental Table S2) and three reference strains from GenBank (see Table 1 for strain names). Identical sequences are represented only once and the number of sequenced strains is indicated on each leaf. Bootstrap values >50% (shown on the node) are expressed as a percentage of 5,000 replicates. Rs strains grouped differently with the two markers, as illustrated by strains K0157, K0024, K0190 and K0018, which were re-sequenced and are marked with an asterisk. Although the average nucleotide difference between groups of Rs with the ITS marker is high, there is little sequence variation within each individual group (clades A, B and C), and fewer strains are resolved than with the RIF marker. Also, ITS sequence from Ralstonia pickettii strain 12J is placed within Rs clade B on the ITS tree, while RIF sequence from the same strain is placed outside Rs clade B on the RIF tree. (TIF) [file pone.0018496.s003.tif]

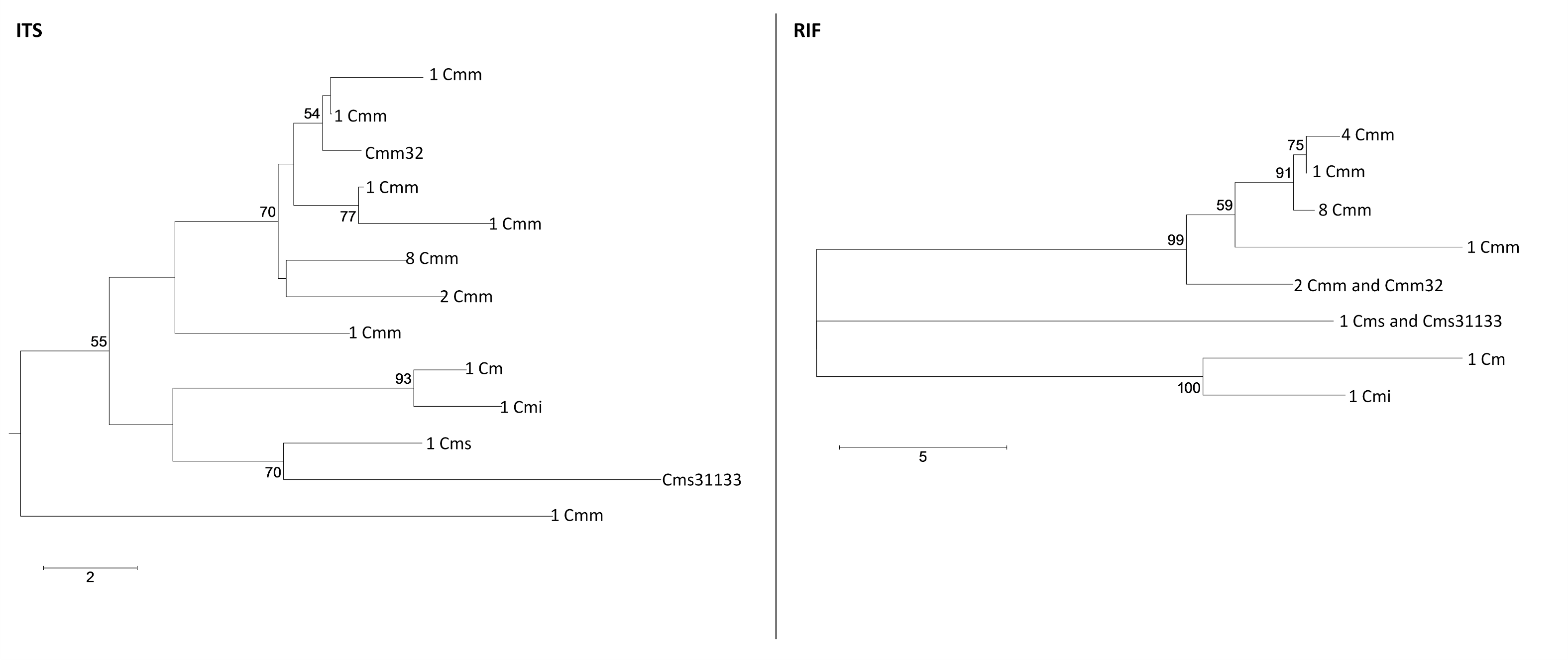

Supplement: Figure S4 — RIF sequences distinguish fewer strains of Clavibacter but produce a more robust tree. Unrooted neighbor-joining trees for the RIF and ITS markers were constructed from nineteen Clavibacter strains from the PBC (Supplemental Table S2) and two reference strains from GenBank (see Table 1 for strain names). Identical sequences are represented only once and the number of sequenced strains is indicated. Bootstrap values >50% (shown at the node) are expressed as a percentage of 5,000 replicates. (TIF) [file pone.0018496.s004.tif]

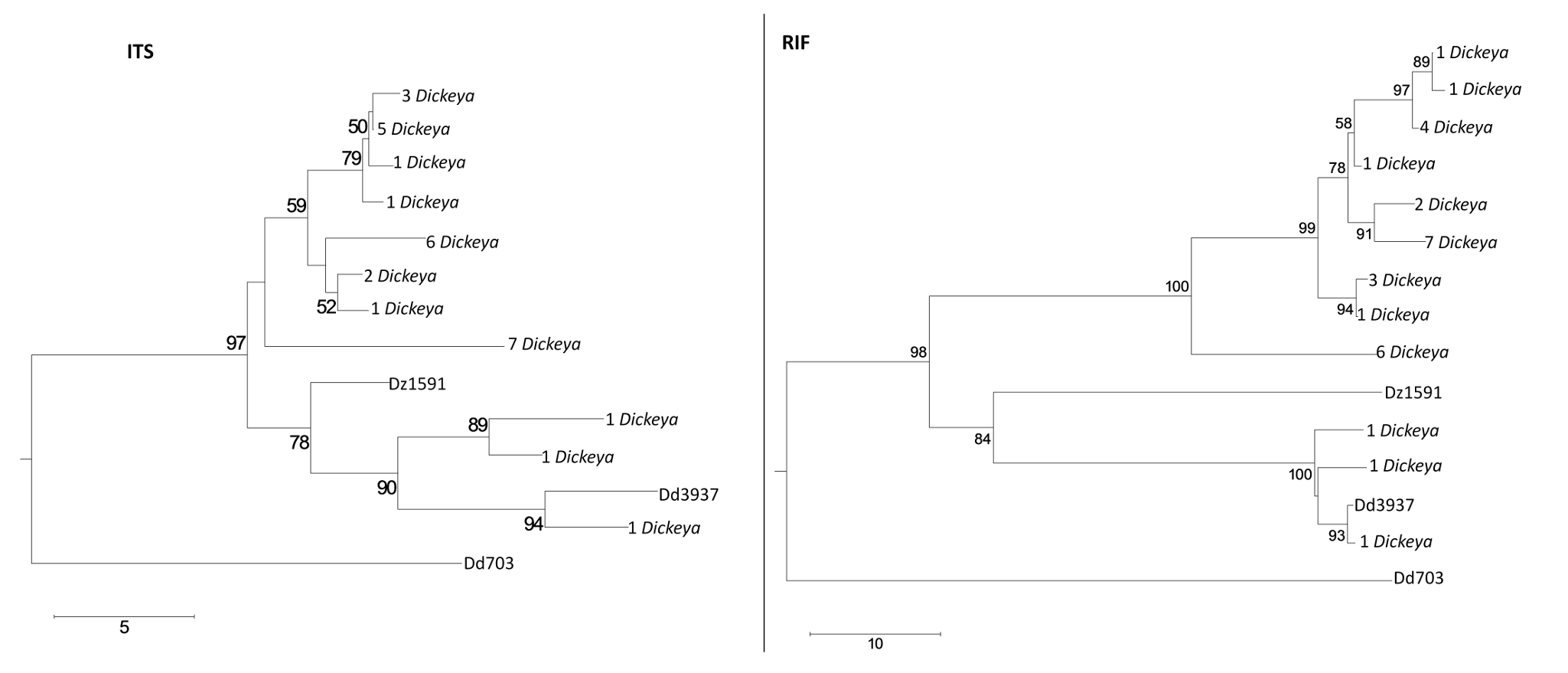

Supplement: Figure S5 — RIF sequences distinguish more Dickeya strains than ITS. Unrooted neighbor-joining trees for the RIF and ITS markers were constructed from twenty-nine Dickeya strains from the PBC (Supplemental Table S2) and three reference strains (with strain names) from GenBank (Supplemental Table S1). Identical sequences are represented only once and the number of sequenced strains is indicated. Bootstrap values >50% (shown at the node) are expressed as a percentage of 5,000 replicates. (TIF) [file pone.0018496.s005.tif]
