# Supplementary material for: Classification of Plant Associated Bacteria Using RIF, a Computationally Derived DNA Marker
Source: PLoS One. 2011 Apr 21;6(4):e18496. doi: 10.1371/journal.pone.0018496 (PMC3080875; doi:10.1371/journal.pone.0018496)
Supplement: Table S1 — Copy number and location of the dnaA genes in 1,067 sequenced NCBI strains. (PDF) [file pone.0018496.s006.pdf]

Supplemental Table S1. Copy number and location of the *dnaA* genes in 1,067 sequenced NCBI strains.

**Strain Name**	**NCBI Accession Number**	**Start of *dnaA***	**End of *dnaA***	**Length**
Blattabacterium sp. (Blattella germanica) Bge	CP001487.1	N/A	N/A	
Blattabacterium sp. (Periplaneta americana) BPLAN	CP001429.2	N/A	N/A	
Candidatus Blochmannia floridanus	BX248583.1	N/A	N/A	
Candidatus Blochmannia pennsylvanicus BPEN	CP000016.1	N/A	N/A	
Candidatus Carsonella ruddii	AP009180.1	N/A	N/A	
Candidatus Hodgkinia cicadicola	CP001266.1	N/A	N/A	
Candidatus Riesia pediculicola	CP001085.1	N/A	N/A	
Candidatus Sulcia muelleri DMIN	CP001981.1	N/A	N/A	
Candidatus Sulcia muelleri SMDSEM	CP001605.1	N/A	N/A	
Candidatus Sulcia muelleri GWSS	CP000770.1	N/A	N/A	
Wigglesworthia brevipalpis	BA000021.3	N/A	N/A	
Chlamydia muridarum	AE002160.2	630061	631431	1371
	AE002160.2	656551	657918	1368
Chlamydia trachomatis 434 Bu	AM884176.1	598341	599711	1371
	AM884176.1	624880	626247	1368
Chlamydia trachomatis A HAR-13	CP000051.1	282141	283511	1371
	CP000051.1	308684	310051	1368
Chlamydia trachomatis B Jali20 OT	FM872308.1	282056	283426	1371
	FM872308.1	308598	309965	1368
Chlamydia trachomatis B TZ1A828 OT	FM872307.1	281987	283357	1371
	FM872307.1	308526	309893	1368
Chlamydia trachomatis D UW 3 CX	AE001273.1	279889	281259	1371
	AE001273.1	306433	307800	1368
Chlamydia trachomatis E 11023 uid43141	CP001890.1	280292	281662	1371

	CP001890.1	306832	308199	1368
Chlamydia trachomatis E 150 uid43143	CP001886.1	280276	281646	1371
	CP001886.1	306817	308184	1368
Chlamydia trachomatis G 11074 uid43149	CP001889.1	280391	281761	1371
	CP001889.1	306938	308305	1368
Chlamydia trachomatis G 11222 uid43147	CP001888.1	280068	281438	1371
	CP001888.1	306610	307977	1368
Chlamydia trachomatis G 9301 uid45851	CP001930.1	280391	281761	1371
	CP001930.1	306938	308305	1368
Chlamydia trachomatis G 9768 uid43145	CP001887.1	280391	281761	1371
	CP001887.1	306937	308304	1368
Chlamydia trachomatis L2b UCH 1 proctitis	AM884177.1	598382	599752	1371
	AM884177.1	624921	626288	1368
Chlamydophila abortus S26 3	CR848038.1	529040	530422	1383
	CR848038.1	412801	414153	1353
Chlamydophila caviae	AE015925.1	535600	536982	1383
	AE015925.1	419450	420802	1353
Chlamydophila felis Fe C-56	AP006861.1	627434	628816	1383
	AP006861.1	742836	744188	1353
Chlamydophila pneumoniae AR39	AE002161.1	490309	491691	1383
	AE002161.1	370232	371635	1404
Chlamydophila pneumoniae CWL029	AE001363.1	349592	350974	1383
	AE001363.1	469612	470964	1353
Chlamydophila pneumoniae J138	BA000008.3	349214	350596	1383
	BA000008.3	469322	470674	1353
Chlamydophila pneumoniae LPCoLN uid17947	CP001713.1	492191	493573	1383
	CP001713.1	371695	373047	1353
Chlamydophila pneumoniae TW 183	AE009440.1	347006	348388	1383
	AE009440.1	467113	468465	1353
Desulfohalobium retbaense DSM 5692	CP001734.1	2830406	2831761	1356

	CP001734.1	932647	934029	1383
Desulfomicrobium baculatum DSM 4028	CP001629.1	3507040	3508320	1281
	CP001629.1	331	1680	1350
Desulfovibrio desulfuricans ATCC 27774	CP001358.1	2640	4013	1374
	CP001358.1	978500	979948	1449
Desulfovibrio desulfuricans G20	CP000112.1	189	1499	1311
	CP000112.1	2337250	2338635	1386
Desulfovibrio magneticus RS 1	AP010904.1	126	1466	1341
	AP010904.1	3486420	3487817	1398
Desulfovibrio salexigens DSM 2638	CP001649.1	4233747	4235051	1305
	CP001649.1	3548083	3549444	1362
Desulfovibrio vulgaris Miyazaki F	CP001197.1	2588354	2589688	1335
	CP001197.1	4	1500	1497
Desulfovibrio vulgaris DP4	CP000527.1	9812	11113	1302
	CP000527.1	1217610	1219124	1515
Desulfovibrio vulgaris Hildenborough	AE017285.1	151	1464	1314
	AE017285.1	2347717	2349192	1476
Fibrobacter succinogenes S85 uid32617	CP001792.1	3396130	3397593	1464
	CP001792.1	128	1453	1326
Lawsonia intracellularis PHE MN1-00	AM180252.1	979314	980636	1323
	AM180252.1	358	1794	1437
Mycobacterium bovis BCG Pasteur 1173P2	AM408590.1	1	1524	1524
	AM408590.1	29668	31191	1524
Mycoplasma mycoides capri GM12 uid19245	CP001621.1	1	1353	1353
	CP001621.1	1078308	1079660	1353
Parachlamydia sp UWE25	BX908798.1	1295440	1296795	1356
	BX908798.1	424280	425665	1386
Pedobacter heparinus DSM 2366	CP001681.1	4313026	4314456	1431
	CP001681.1	2753213	2754643	1431
Pirellula sp	BX119912.1	6246565	6247827	1263

	BX119912.1	889863	891638	1776
Pirellula staleyi DSM 6068 uid29845	CP001848.1	1279958	1281154	1197
	CP001848.1	234	1946	1713
Salinibacter ruber DSM 13855	CP000159.1	1718032	1719726	1695
	CP000159.1	2	1576	1575
Thermincola JR uid41467	CP002028.1	1137843	1138862	1020
	CP002028.1	98	1453	1356
Acaryochloris marina MBIC11017	CP000828.1	3124142	3125509	1368
Acetobacter pasteurianus IFO 3283 1	AP011121.1	1291083	1292516	1434
Acetobacter pasteurianus IFO 3283 1 42C uid31141	AP011163.1	1291083	1292516	1434
Acetobacter pasteurianus IFO 3283 12 uid32203	AP011170.1	1291077	1292510	1434
Acetobacter pasteurianus IFO 3283 22 uid31135	AP011142.1	1292094	1293527	1434
Acetobacter pasteurianus IFO 3283 26 uid31137	AP011149.1	1292094	1293527	1434
Acetobacter pasteurianus IFO 3283 3 uid31131	AP011128.1	1292094	1293527	1434
Acetobacter pasteurianus IFO 3283 32 uid31139	AP011156.1	1291077	1292510	1434
Acetobacter pasteurianus IFO 3283 7 uid31133	AP011135.1	1291077	1292510	1434
Acholeplasma laidlawii PG 8A	CP000896.1	289	1647	1359
Acidaminococcus fermentans DSM 20731 uid33685	CP001859.1	34	1194	1161
Acidaminococcus fermentans DSM 20731 uid33685	CP001859.1	2751	4139	1389
Acidimicrobium ferrooxidans DSM 10331	CP001631.1	87	1463	1377
Acidiphilium cryptum JF-5	CP000697.1	261	1709	1449
Acidithiobacillus ferrooxidans ATCC 23270	CP001219.1	2980841	2982199	1359
Acidithiobacillus ferrooxidans ATCC 53993	CP001132.1	252	1610	1359
Acidobacterium capsulatum ATCC 51196	CP001472.1	2480464	2481900	1437
Acidothermus cellulolyticus 11B	CP000481.1	75	1517	1443
Acidovorax avenae citrulli AAC00-1	CP000512.1	71	1498	1428
Acidovorax JS42	CP000539.1	4429148	4430569	1422
Acinetobacter baumannii AB0057	CP001182.1	21687	23084	1398
Acinetobacter baumannii AB307 294	CP001172.1	95	1492	1398
Acinetobacter baumannii ACICU	CP000863.1	5370	6767	1398

Acinetobacter baumannii ATCC 17978	CP000521.1	95	1492	1398
Acinetobacter baumannii AYE	CU459141.1	170	1567	1398
Acinetobacter baumannii SDF	CU468230.2	367	1764	1398
Acinetobacter sp ADP1	CR543861.1	201	1598	1398
Actinobacillus pleuropneumoniae L20	CP000569.1	63	1547	1485
Actinobacillus pleuropneumoniae serovar 3 JL03	CP000687.1	2	1486	1485
Actinobacillus pleuropneumoniae serovar 7 AP76	CP001091.1	2	1486	1485
Actinobacillus succinogenes 130Z	CP000746.1	252	1619	1368
Actinosynnema mirum DSM 43827	CP001630.1	98	1831	1734
Aeromonas hydrophila ATCC 7966	CP000462.1	223	1593	1371
Aeromonas salmonicida A449	CP000644.1	201	1571	1371
Aggregatibacter actinomycetemcomitans D11S 1 uid40107	CP001733.1	1895609	1896970	1362
Aggregatibacter aphrophilus NJ8700	CP001607.1	53733	55094	1362
Agrobacterium radiobacter K84	CP000628.1	496696	498246	1551
Agrobacterium tumefaciens C58 Cereon	AE007869.2	317547	319109	1563
Agrobacterium vitis S4	CP000633.1	336131	337705	1575
Akkermansia muciniphila ATCC BAA 835	CP001071.1	1882	3288	1407
Alcanivorax borkumensis SK2	AM286690.1	1	1425	1425
Alicyclobacillus acidocaldarius DSM 446	CP001727.1	112	1476	1365
Aliivibrio salmonicida LFI1238	FM178379.1	7427	8833	1407
Alkalilimnicola ehrlichei MLHE-1	CP000453.1	75	1430	1356
Alkaliphilus metalliredigens QYMF	CP000724.1	50	1396	1347
Alkaliphilus oremlandii OhILAs	CP000853.1	49	1404	1356
Allochromatium vinosum DSM 180 uid32547	CP001896.1	232	1593	1362
alpha proteobacterium IMCC1322 uid28081	CP001751.1	1721041	1722573	1533
Alteromonas macleodii Deep ecotype	CP001103.1	759	2360	1602
Aminobacterium colombiense DSM 12261 uid32587	CP001997.1	141	1469	1329
Ammonifex degensii KC4 uid12390	CP001785.1	188	1522	1335
Anabaena variabilis ATCC 29413	CP000117.1	119	1501	1383
Anaerocellum thermophilum DSM 6725	CP001393.1	45	1409	1365

Anaerococcus prevotii DSM 20548	CP001708.1	254	1633	1380
Anaeromyxobacter dehalogenans 2CP 1	CP001359.1	248	1624	1377
Anaeromyxobacter dehalogenans 2CP-C	CP000251.1	22	1395	1374
Anaeromyxobacter Fw109-5	CP000769.1	48	1430	1383
Anaeromyxobacter K	CP001131.1	36	1412	1377
Anaplasma centrale Israel uid32765	CP001759.1	850199	851614	1416
Anaplasma marginale Florida	CP001079.1	387758	389173	1416
Anaplasma marginale St Maries	CP000030.1	389525	390940	1416
Anaplasma phagocytophilum HZ	CP000235.1	501807	503186	1380
Anoxybacillus flavithermus WK1	CP000922.1	401	1744	1344
Aquifex aeolicus	AE000657.1	208642	209841	1200
Arcanobacterium haemolyticum DSM 20595 uid37925	CP002045.1	77	1711	1635
Arcobacter butzleri RM4018	CP000361.1	1	1317	1317
Arcobacter nitrofigilis DSM 7299 uid32593	CP001999.1	71	1384	1314
Aromatoleum aromaticum EbN1	CR555306.1	1693960	1695405	1446
Arthrobacter aurescens TC1	CP000474.1	1	1419	1419
Arthrobacter chlorophenolicus A6	CP001341.1	177	1598	1422
Arthrobacter FB24	CP000454.1	5	1429	1425
Atopobium parvulum DSM 20469	CP001721.1	1200	2729	1530
Azoarcus BH72	AM406670.1	1	1443	1443
Azorhizobium caulinodans ORS 571	AP009384.1	1138239	1139573	1335
Azospirillum B510 uid32551	AP010946.1	3055827	3057344	1518
Azotobacter vinelandii DJ	CP001157.1	101	1537	1437
Bacillus amyloliquefaciens FZB42	CP000560.1	412	1752	1341
Bacillus anthracis A0248	CP001598.1	307	1647	1341
Bacillus anthracis Ames	AE017334.2	407	1747	1341
Bacillus anthracis Ames 581	AE017334.2	407	1747	1341
Bacillus anthracis CDC 684	CP001215.1	281	1621	1341
Bacillus anthracis str Sterne	AE017225.1	408	1748	1341
Bacillus cereus 03BB102	CP001407.1	292	1632	1341

Bacillus cereus AH187	CP001177.1	409	1749	1341
Bacillus cereus AH820	CP001283.1	408	1748	1341
Bacillus cereus ATCC 10987	AE017194.1	408	1748	1341
Bacillus cereus ATCC14579	AE016877.1	281	1621	1341
Bacillus cereus B4264	CP001176.1	407	1747	1341
Bacillus cereus cytotoxis NVH 391-98	CP000764.1	521	1861	1341
Bacillus cereus G9842	CP001186.1	246	1586	1341
Bacillus cereus Q1	CP000227.1	408	1748	1341
Bacillus cereus ZK	CP000001.1	408	1748	1341
Bacillus clausii KSM-K16	AP006627.1	183	1538	1356
Bacillus halodurans	BA000004.3	584	1933	1350
Bacillus licheniformis ATCC 14580	CP000002.3	507	1847	1341
Bacillus licheniformis DSM 13	AE017333.1	311	1651	1341
Bacillus megaterium DSM319 uid42425	CP001982.1	1	1344	1344
Bacillus megaterium QM B1551 uid30165	CP001983.1	1	1344	1344
Bacillus pseudofirmus OF4 uid28811	CP001878.1	1573534	1574886	1353
Bacillus pumilus SAFR-032	CP000813.1	1	1341	1341
Bacillus selenitireducens MLS10 uid13376	CP001791.1	654	2009	1356
Bacillus subtilis	AL009126.3	410	1750	1341
Bacillus thuringiensis Al Hakam	CP000485.1	351	1748	1398
Bacillus thuringiensis BMB171 uid43631	CP001903.1	283	1623	1341
Bacillus thuringiensis konkukian	AE017355.1	409	1749	1341
Bacillus tusciae DSM 2912 uid31345	CP002017.1	369	1721	1353
Bacillus weihenstephanensis KBAB4	CP000903.1	106	1446	1341
Bacteroides fragilis NCTC 9434	CR626927.1	4268926	4270356	1431
Bacteroides fragilis YCH46	AP006841.1	4362998	4364428	1431
Bacteroides thetaiotaomicron VPI-5482	AE015928.1	2697457	2698869	1413
Bacteroides vulgatus ATCC 8482	CP000139.1	1	1416	1416
Bartonella bacilliformis KC583	CP000524.1	1324320	1325894	1575
Bartonella grahamii as4aup	CP001562.1	153867	155438	1572

Bartonella henselae Houston-1	BX897699.1	158771	160342	1572
Bartonella quintana Toulouse	BX897700.1	146196	147767	1572
Bartonella tribocorum CIP 105476	AM260525.1	156702	158273	1572
Bdellovibrio bacteriovorus	BX842601.2	1	1416	1416
Beijerinckia indica ATCC 9039	CP001016.1	1509	3041	1533
Beutenbergia cavernae DSM 12333	CP001618.1	322	1791	1470
Bifidobacterium adolescentis ATCC 15703	AP009256.1	1	1500	1500
Bifidobacterium animalis lactis AD011	CP001213.1	165	1916	1752
Bifidobacterium animalis lactis BB 12 uid42883	CP001853.1	635770	637533	1764
Bifidobacterium animalis lactis Bl 4	CP001515.1	134	1885	1752
Bifidobacterium animalis lactis DSM 10140	CP001606.1	134	1885	1752
Bifidobacterium animalis lactis V9 uid32515	CP001892.1	134	1885	1752
Bifidobacterium dentium Bd1 uid17583	CP001750.1	1	1503	1503
Bifidobacterium longum	AE014295.3	1624263	1625765	1503
Bifidobacterium longum DJO10A	CP000605.1	1665929	1667431	1503
Bifidobacterium longum infantis ATCC 15697	CP001095.1	210	1712	1503
Bifidobacterium longum JDM301 uid47579	CP002010.1	210	1712	1503
Bordetella avium 197N	AM167904.1	3709832	3711283	1452
Bordetella bronchiseptica	BX470250.1	5310125	5311567	1443
Bordetella parapertussis	BX470249.1	4744528	4745937	1410
Bordetella pertussis	BX470248.1	504563	505972	1410
Bordetella petrii	AM902716.1	1	1437	1437
Borrelia afzelii PKo	CP000395.1	460469	461926	1458
Borrelia burgdorferi	CP001205.1	456576	458036	1461
Borrelia burgdorferi ZS7	CP001205.1	452671	454134	1464
Borrelia duttonii Ly	CP000976.1	468262	469716	1455
Borrelia garinii PBi	CP000013.1	458789	460243	1455
Borrelia hermsii DAH	CP000048.1	460217	461671	1455
Borrelia recurrentis A1	CP000993.1	474985	476439	1455
Borrelia turicatae 91E135	CP000049.1	457651	459105	1455

Brachybacterium faecium DSM 4810	CP001643.1	44	1741	1698
Brachyspira hyodysenteriae WA1	CP001357.1	239539	240924	1386
Brachyspira murdochii DSM 12563 uid29543	CP001959.1	132	1511	1380
Bradyrhizobium BTAi1	CP000494.1	571	1998	1428
Bradyrhizobium japonicum	BA000040.2	893712	895124	1413
Bradyrhizobium ORS278	CU234118.1	936	2366	1431
Brevibacillus brevis NBRC 100599	AP008955.1	465	1826	1362
Brucella abortus bv 1 9 941	AE017223.1	784	2274	1491
Brucella abortus S19	CP000887.1	634	2274	1641
Brucella canis ATCC 23365	CP000872.1	634	2274	1641
Brucella melitensis	CP001488.1	2001077	2002660	1584
Brucella melitensis ATCC 23457	CP001488.1	634	2274	1641
Brucella melitensis biovar Abortus	AM040264.1	784	2274	1491
Brucella microti CCM 4915	CP001578.1	784	2274	1491
Brucella ovis	CP000708.1	785	2275	1491
Brucella suis 1330	AE014291.4	784	2274	1491
Brucella suis ATCC 23445	CP000911.1	634	2274	1641
Buchnera aphidicola	CP001161.1	13114	14487	1374
Buchnera aphidicola 5A Acyrthosiphon pisum	CP001161.1	12554	13918	1365
Buchnera aphidicola Cc Cinara cedri	CP000263.1	5647	6960	1314
Buchnera aphidicola Sg	AE013218.1	12397	13761	1365
Buchnera aphidicola Tuc7 Acyrthosiphon pisum	CP001158.1	12554	13918	1365
Buchnera sp	BA000003.2	12554	13918	1365
Burkholderia 383	CP000151.1	76	1653	1578
Burkholderia ambifaria AMMD	CP000440.1	203	1780	1578
Burkholderia ambifaria MC40 6	CP001025.1	302	1879	1578
Burkholderia CCGE1002 uid37719	CP002013.1	363	1961	1599
Burkholderia cenocepacia AU 1054	CP000378.1	2802249	2803934	1686
Burkholderia cenocepacia HI2424	CP000458.1	178	1755	1578
Burkholderia cenocepacia J2315	AM747720.1	467298	468875	1578

Burkholderia cenocepacia MC0 3	CP000958.1	303	1880	1578
Burkholderia glumae BGR1	CP001503.1	100	1704	1605
Burkholderia mallei ATCC 23344	CP000010.1	70	1626	1557
Burkholderia mallei NCTC 10229	CP000546.1	2267135	2268736	1602
Burkholderia mallei NCTC 10247	CP000548.1	25	1626	1602
Burkholderia mallei SAVP1	CP000526.1	2826231	2827832	1602
Burkholderia multivorans ATCC 17616 JGI	CP000868.1	74	1648	1575
Burkholderia multivorans ATCC 17616 Tohoku	AP009385.1	74658	76232	1575
Burkholderia phymatum STM815	CP001043.1	362	1918	1557
Burkholderia phytofirmans PsJN	CP001052.1	299	1936	1638
Burkholderia pseudomallei 1106a	CP000572.1	101194	102795	1602
Burkholderia pseudomallei 1710b	CP000124.1	310638	312239	1602
Burkholderia pseudomallei 668	CP000570.1	85283	86884	1602
Burkholderia pseudomallei K96243	BX571965.1	85351	86952	1602
Burkholderia thailandensis E264	CP000086.1	3690347	3691957	1611
Burkholderia vietnamiensis G4	CP000614.1	294	1871	1578
Burkholderia xenovorans LB400	CP000270.1	192	1826	1635
Caldicellulosiruptor saccharolyticus DSM 8903	CP000679.1	642	2006	1365
Campylobacter concisus 13826	CP000792.1	1	1311	1311
Campylobacter curvus 525 92	CP000767.1	1	1311	1311
Campylobacter fetus 82-40	CP000487.1	1	1311	1311
Campylobacter hominis ATCC BAA-381	CP000776.1	1	1314	1314
Campylobacter jejuni	CP000025.1	1	1323	1323
Campylobacter jejuni 81116	CP000814.1	1	1323	1323
Campylobacter jejuni 81-176	CP000538.1	1	1323	1323
Campylobacter jejuni doylei 269 97	CP000768.1	1	1323	1323
Campylobacter jejuni IA3902 uid28907	CP001876.1	1	1323	1323
Campylobacter jejuni RM1221	CP000025.1	1	1323	1323
Campylobacter lari RM2100	CP000932.1	1	1329	1329
Candidatus Accumulibacter phosphatis clade IIA UW 1	CP001715.1	239	1660	1422

Candidatus Amoebophilus asiaticus 5a2	CP001102.1	513	1946	1434
Candidatus Azobacteroides pseudotrichonymphae genomovar CFP2	AP010656.1	1	1368	1368
Candidatus Desulfococcus oleovorans Hxd3	CP000859.1	50	1432	1383
Candidatus Desulforudis audaxviator MP104C	CP000860.1	103	1440	1338
Candidatus Hamiltonella defensa 5AT Acyrthosiphon pisum	CP001277.1	1579736	1581100	1365
Candidatus Koribacter versatilis Ellin345	CP000360.1	33	1418	1386
Candidatus Liberibacter asiaticus psy62	CP001677.2	643376	644884	1509
Candidatus Pelagibacter ubique HTCC1062	CP000084.1	398915	400249	1335
Candidatus Phytoplasma australiense	AM422018.1	1	1368	1368
Candidatus Phytoplasma mali	CU469464.1	250608	251975	1368
Candidatus Ruthia magnifica Cm Calyptogena magnifica	CP000488.1	39	1331	1293
Candidatus Vesicomyosocius okutanii HA	AP009247.1	1	1293	1293
Capnocytophaga ochracea DSM 7271	CP001632.1	2323139	2324560	1422
Carboxydothermus hydrogenoformans Z-2901	CP000141.1	2399937	2401301	1365
Catenulispora acidiphila DSM 44928	CP001700.1	370	2082	1713
Caulobacter crescentus	CP001340.1	5636	7108	1473
Caulobacter crescentus NA1000	CP001340.1	5636	7108	1473
Caulobacter K31	CP000927.1	389	1852	1464
Caulobacter segnis ATCC 21756 uid37277	CP002008.1	20	1495	1476
Cellulomonas flavigena DSM 20109 uid19707	CP001964.1	68	1672	1605
Cellvibrio japonicus Ueda107	CP000934.1	1	1605	1605
Chitinophaga pinensis DSM 2588	CP001699.1	126	1559	1434
Chlorobaculum parvum NCIB 8327	CP001099.1	167	1648	1482
Chlorobium chlorochromatii CaD3	CP000108.1	346	1824	1479
Chlorobium limicola DSM 245	CP001097.1	162	1637	1476
Chlorobium luteolum DSM 273	CP000096.1	2363373	2364842	1470
Chlorobium phaeobacteroides BS1	CP001101.1	1	1476	1476
Chlorobium phaeobacteroides DSM 266	CP000492.1	1	1473	1473
Chlorobium tepidum TLS	AE006470.1	1398	2879	1482
Chloroflexus aggregans DSM 9485	CP001337.1	208	1644	1437

Chloroflexus aurantiacus J 10 fl	CP000909.1	336	1775	1440
Chloroflexus Y 400 fl	CP001364.1	294	1733	1440
Chloroherpeton thalassium ATCC 35110	CP001100.1	1	1491	1491
Chromobacterium violaceum	AE016825.1	245	1648	1404
Chromohalobacter salexigens DSM 3043	CP000285.1	93	1580	1488
Citrobacter koseri ATCC BAA-895	CP000822.1	46474	47808	1335
Citrobacter rodentium ICC168 uid34685	FN543502.1	4262871	4264277	1407
Clavibacter michiganensis NCPPB 382	AM711867.1	1	1434	1434
Clavibacter michiganensis sepedonicus	AM849034.1	1	1431	1431
Clostridiales genomosp BVAB3 UPII9 5 uid42555	CP001850.1	96822	98138	1317
Clostridium acetobutylicum	AE001437.1	467	1807	1341
Clostridium beijerinckii NCIMB 8052	CP000721.1	280	1629	1350
Clostridium botulinum A	CP000962.1	1	1347	1347
Clostridium botulinum A ATCC 19397	CP000726.1	1	1347	1347
Clostridium botulinum A Hall	CP000727.1	1	1347	1347
Clostridium botulinum A2 Kyoto	CP001581.1	157	1494	1338
Clostridium botulinum A3 Loch Maree	CP000962.1	14	1351	1338
Clostridium botulinum B Eklund 17B	CP001056.1	175	1545	1371
Clostridium botulinum B1 Okra	CP000939.1	38	1375	1338
Clostridium botulinum Ba4 657	CP001083.1	164	1510	1347
Clostridium botulinum E3 Alaska E43	CP001078.1	127	1497	1371
Clostridium botulinum F 230613 uid47575	CP002011.1	1	1338	1338
Clostridium botulinum F Langeland	CP000728.1	1	1338	1338
Clostridium cellulolyticum H10	CP001348.1	27	1349	1323
Clostridium difficile 630	AM180355.1	1	1320	1320
Clostridium difficile R20291	FN545816.1	1	1320	1320
Clostridium kluyveri DSM 555	CP000673.1	1	1356	1356
Clostridium kluyveri NBRC 12016	AP009049.1	1	1356	1356
Clostridium novyi NT	CP000382.1	2546147	2547493	1347
Clostridium perfringens	CP000246.1	410	1783	1374

Clostridium perfringens ATCC 13124	CP000246.1	411	1784	1374
Clostridium perfringens SM101 uid12521	CP000312.1	236	1609	1374
Clostridium phytofermentans ISDg	CP000885.1	75	1436	1362
Clostridium tetani E88	AE015927.1	51225	52253	1029
Clostridium thermocellum ATCC 27405	CP000568.1	2834293	2835624	1332
Colwellia psychrerythraea 34H	CP000083.1	72	1457	1386
Comamonas testosteroni CNB 1 uid29203	CP001220.1	1	1407	1407
Conexibacter woesei DSM 14684 uid20745	CP001854.1	80	1441	1362
Coprothermobacter proteolyticus DSM 5265	CP001145.1	57624	59255	1632
Coraliomargarita akajimensis DSM 45221 uid33365	CP001998.1	490	1899	1410
Corynebacterium aurimucosum ATCC 700975	CP001601.1	1	1641	1641
Corynebacterium diphtheriae	BX248353.1	19	1677	1659
Corynebacterium efficiens YS-314	BA000035.2	1	1722	1722
Corynebacterium glutamicum ATCC 13032 Bielefeld	BX927147.1	1	1575	1575
Corynebacterium glutamicum ATCC 13032 Kitasato	BA000036.3	1	1575	1575
Corynebacterium glutamicum R	AP009044.1	1	1575	1575
Corynebacterium jeikeium K411	CR931997.1	1	1752	1752
Corynebacterium kroppenstedtii DSM 44385	CP001620.1	1	1818	1818
Corynebacterium urealyticum DSM 7109	AM942444.1	1	1761	1761
Coxiella burnetii	CP001019.1	140	1495	1356
Coxiella burnetii CbuG Q212	CP001019.1	140	1495	1356
Coxiella burnetii CbuK Q154	CP001020.1	140	1495	1356
Coxiella burnetii Dugway 7E9-12	CP000733.1	140	1495	1356
Coxiella burnetii RSA 331	CP000890.1	140	1495	1356
Cronobacter turicensis uid39965	FN543093.1	34550	36010	1461
Cryptobacterium curtum DSM 15641	CP001682.1	130	1650	1521
Cupriavidus metallidurans CH34 uid250	CP000352.1	104	1843	1740
Cupriavidus taiwanensis	CU633749.1	707	2476	1770
Cyanobacteria bacterium Yellowstone A-Prime	CP000239.1	71	1474	1404
Cyanobacteria bacterium Yellowstone B-Prime	CP000240.1	64	1467	1404

Cyanothece ATCC 51142	CP000806.1	3796973	3798340	1368
Cyanothece PCC 7424	CP001291.1	90	1451	1362
Cyanothece PCC 7425	CP001344.1	48	1430	1383
Cyanothece PCC 8801	CP001287.1	1254	2615	1362
Cyanothece PCC 8802	CP001701.1	98	1459	1362
Cytophaga hutchinsonii ATCC 33406	CP000383.1	3450532	3451947	1416
Dechloromonas aromatica RCB	CP000089.1	151	1548	1398
Deferribacter desulfuricans SSM1 uid37285	AP011529.1	2230975	2232306	1332
Deferribacter desulfuricans SSM1 uid37285	AP011529.1	234637	236244	1608
Dehalococcoides BAV1	CP000688.1	245	1579	1335
Dehalococcoides CBDB1	AJ965256.1	261	1595	1335
Dehalococcoides ethenogenes 195	CP000027.1	261	1598	1338
Dehalococcoides GT uid36645	CP001924.1	245	1579	1335
Dehalococcoides VS uid18811	CP001827.1	259	1593	1335
Deinococcus deserti VCD115	CP001114.1	42	1421	1380
Deinococcus geothermalis DSM 11300	CP000359.1	222	1634	1413
Deinococcus radiodurans	AE000513.1	1904	3268	1365
Delftia acidovorans SPH-1	CP000884.1	16	1422	1407
Denitrovibrio acetiphilus DSM 12809 uid29431	CP001968.1	36	1376	1341
Desulfatibacillum alkenivorans AK 1	CP001322.1	423	1787	1365
Desulfitobacterium hafniense DCB 2	CP001336.1	93	1442	1350
Desulfitobacterium hafniense Y51	AP008230.1	1	1350	1350
Desulfobacterium autotrophicum HRM2	CP001087.1	2112000	2113409	1410
Desulfotalea psychrophila LSv54	CR522870.1	301842	303305	1464
Desulfotomaculum acetoxidans DSM 771	CP001720.1	31	1371	1341
Desulfotomaculum reducens MI-1	CP000612.1	365	1690	1326
Desulfurivibrio alkaliphilus AHT2 uid33629	CP001940.1	505	1860	1356
Diaphorobacter TPSY uid29975	CP001392.1	70	1491	1422
Dichelobacter nodosus VCS1703A	CP000513.1	130	1458	1329
Dickeya dadantii Ech586 uid33667	CP001836.1	270	1658	1389

Dickeya dadantii Ech703	CP001654.1	188	1576	1389
Dickeya zeae Ech1591	CP001655.1	35	1429	1395
Dictyoglomus thermophilum H 6 12	CP001146.1	1662563	1663888	1326
Dictyoglomus turgidum DSM 6724	CP001251.1	75	1406	1332
Dinoroseobacter shibae DFL 12	CP000830.1	3544704	3546086	1383
Dyadobacter fermentans DSM 18053	CP001619.1	2	1444	1443
Edwardsiella ictaluri 93 146	CP001600.1	60	1451	1392
Edwardsiella tarda EIB202 uid28539	CP001135.1	1	1323	1323
Eggerthella lenta DSM 2243	CP001726.1	1306	2868	1563
Ehrlichia canis Jake	CP000107.1	413518	414876	1359
Ehrlichia chaffeensis Arkansas	CP000236.1	822485	823879	1395
Ehrlichia ruminantium Gardel	CR925677.1	483666	485060	1395
Ehrlichia ruminantium str. Welgevonden CIRAD	CR925678.1	486450	487844	1395
Ehrlichia ruminantium Welgevonden UPSA	CR767821.1	506593	507987	1395
Elusimicrobium minutum Pei191	CP001055.1	556	1914	1359
Enterobacter 638	CP000653.1	518	1969	1452
Enterobacter cloacae ATCC 13047 uid45793	CP001918.1	82	1410	1329
Enterobacter sakazakii ATCC BAA-894	CP000783.1	3924054	3925370	1317
Enterococcus faecalis V583	AE016830.1	59	1402	1344
Erwinia amylovora ATCC 49946 uid43757	FN666575.1	3772963	3774354	1392
Erwinia amylovora CFBP1430 uid46805	FN434113.1	3772705	3774096	1392
Erwinia carotovora atroseptica SCRI1043	BX950851.1	4983699	4985096	1398
Erwinia pyrifoliae DSM 12163 uid37877	FN392235.1	3995572	3996999	1428
Erwinia pyrifoliae Ep1 96 uid34779	FP236842.1	3995609	3997000	1392
Erwinia tasmaniensis	CU468135.1	3850468	3851859	1392
Erythrobacter litoralis HTCC2594	CP000157.1	2142392	2143858	1467
Escherichia coli 127 H6 E2348 69	FM180568.1	4179650	4181053	1404
Escherichia coli 42 uid40647	FN554766.1	4317269	4318672	1404
Escherichia coli 536	CP000247.1	4066823	4068157	1335
Escherichia coli 55989	CU928145.2	4257487	4258890	1404

Escherichia coli APEC O1	CP000468.1	4176691	4178106	1416
Escherichia coli B REL606	CP000819.1	3842687	3844090	1404
Escherichia coli BL21 DE3	CP001509.3	347	1750	1404
Escherichia coli BL21 DE3 uid20713	CP001509.3	3771122	3772525	1404
Escherichia coli BL21 DE3 uid28965	AM946981.1	3769200	3770603	1404
Escherichia coli BW2952	CP001396.1	3768682	3770085	1404
Escherichia coli C ATCC 8739	CP000946.1	48	1451	1404
Escherichia coli CFT073	AE014075.1	2875039	2875785	747
Escherichia coli DH1 uid30031	CP001637.1	33	1436	1404
Escherichia coli E24377A	CP000800.1	4199088	4200491	1404
Escherichia coli ED1a	CU928162.2	4319836	4321239	1404
Escherichia coli HS	CP000802.1	3909497	3910900	1404
Escherichia coli IAI1	CU928160.2	3953613	3955016	1404
Escherichia coli IAI39	CU928164.2	4482664	4484067	1404
Escherichia coli IHE3034 uid43693	CP001969.1	4256793	4258196	1404
Escherichia coli K 12 substr DH10B	CP000948.1	3977933	3979336	1404
Escherichia coli K 12 substr MG1655	U00096.2	3880349	3881752	1404
Escherichia coli K 12 substr W3110	AP009048.1	3756686	3758089	1404
Escherichia coli O103 H2 12009 uid32511	AP010958.1	4580256	4581659	1404
Escherichia coli O111 H 11128 uid32513	AP010960.1	4551702	4553105	1404
Escherichia coli O157 H7 EC4115	CP001164.1	4771663	4773066	1404
Escherichia coli O157 H7 TW14359	CP001368.1	4727706	4729109	1404
Escherichia coli O157H7	AE005174.2	4668559	4669962	1404
Escherichia coli O157H7 EDL933	AE005174.2	4737542	4738945	1404
Escherichia coli O26 H11 11368 uid32509	AP010953.1	4904905	4906308	1404
Escherichia coli O55 H7 CB9615 uid42729	CP001846.1	4556053	4557456	1404
Escherichia coli S88	CU928161.2	4091404	4092807	1404
Escherichia coli SE11	AP009240.1	4131342	4132745	1404
Escherichia coli SE15 uid19053	AP009378.1	3840836	3842239	1404
Escherichia coli SMS 3 5	CP000970.1	4158994	4160397	1404

Escherichia coli UMN026	CU928163.2	4366052	4367455	1404
Escherichia coli UTI89	CP000243.1	4143894	4145297	1404
Escherichia fergusonii ATCC 35469	CU928158.2	4102156	4103568	1413
Eubacterium eligens ATCC 27750	CP001104.1	1	1356	1356
Eubacterium rectale ATCC 33656	CP001107.1	1	1362	1362
Exiguobacterium AT1b	CP001615.1	1699111	1700490	1380
Exiguobacterium sibiricum 255 15	CP001022.1	427	1821	1395
Fervidobacterium nodosum Rt17-B1	CP000771.1	43	1377	1335
Finegoldia magna ATCC 29328	AP008971.1	365	1822	1458
Flavobacteriaceae bacterium 3519 10	CP001673.1	543627	545093	1467
Flavobacterium johnsoniae UW101	CP000685.1	3174	4601	1428
Flavobacterium psychrophilum JIP02 86	AM398681.1	1399584	1401020	1437
Francisella philomiragia ATCC 25017	CP000937.1	896082	897557	1476
Francisella tularensis FSC 198	AM286280.1	1	1521	1521
Francisella tularensis holarctica	CP000803.1	1	1476	1476
Francisella tularensis holarctica	CP000803.1	275	1750	1476
Francisella tularensis holarctica FTNF002 0	CP000803.1	1	1476	1476
Francisella tularensis holarctica OSU18	CP000437.1	1	1476	1476
Francisella tularensis mediasiatica FSC147	CP000915.1	130	1521	1392
Francisella tularensis NE061598 uid38289	CP001633.1	1	1521	1521
Francisella tularensis novicida U112	CP000439.1	145	1620	1476
Francisella tularensis tularensis	AJ749949.2	1	1521	1521
Francisella tularensis WY96-3418	CP000608.1	46	1521	1476
Frankia alni ACN14a	CT573213.2	424	2028	1605
Frankia CcI3	CP000249.1	35	1723	1689
Frankia EAN1pec	CP000820.1	71	1654	1584
Fusobacterium nucleatum	AE009951.2	639955	641868	1914
Gardnerella vaginalis 409 5 uid31001	CP001849.1	53866	55485	1620
Gemmatimonas aurantiaca T 27	AP009153.1	101	1519	1419
Geobacillus C56 T3 uid41701	CP002050.1	219	1571	1353

Geobacillus kaustophilus HTA426	BA000043.1	88	1440	1353
Geobacillus thermodenitrificans NG80-2	CP000557.1	209	1561	1353
Geobacillus WCH70	CP001638.1	27	1379	1353
Geobacillus Y412MC10 uid27777	CP001793.1	166	1512	1347
Geobacillus Y412MC61 uid30537	CP001794.1	123	1475	1353
Geobacter bemidjiensis Bem	CP001124.1	166	1542	1377
Geobacter FRC 32	CP001390.1	185	1522	1338
Geobacter lovleyi SZ	CP001089.1	261	1640	1380
Geobacter M21	CP001661.1	119	1501	1383
Geobacter metallireducens GS-15	CP000148.1	261	1613	1353
Geobacter sulfurreducens	AE017180.1	30	1367	1338
Geobacter uraniumreducens Rf4	CP000698.1	46	1395	1350
Geodermatophilus obscurus DSM 43160 uid29547	CP001867.1	314	2068	1755
Gloeobacter violaceus	BA000045.2	1583759	1585087	1329
Gluconacetobacter diazotrophicus PAl 5 FAPERJ	AM889285.1	1798138	1799601	1464
Gluconacetobacter diazotrophicus PAl 5 JGI	CP001189.1	5	1438	1434
Gluconobacter oxydans 621H	CP000009.1	155	1594	1440
Gordonia bronchialis DSM 43247 uid29549	CP001802.1	333	1862	1530
Gramella forsetii KT0803	CU207366.1	699578	700087	510
Haemophilus ducreyi 35000HP	AE017143.1	678427	679773	1347
Haemophilus influenzae	CP000671.1	1055909	1057273	1365
Haemophilus influenzae PittEE	CP000671.1	1389072	1390436	1365
Haemophilus influenzae PittGG	CP000672.1	1584551	1585915	1365
Haemophilus parasuis SH0165	CP001321.1	1478942	1480276	1335
Haemophilus somnus 129PT	CP000436.1	122211	123569	1359
Haemophilus somnus 2336	CP000947.1	279	1646	1368
Hahella chejuensis KCTC 2396	CP000155.1	378	1781	1404
Haliangium ochraceum DSM 14365 uid28711	CP001804.1	109	1605	1497
Halorhodospira halophila SL1	CP000544.1	1333575	1334924	1350
Halothermothrix orenii H 168	CP001098.1	593	1984	1392

Halothiobacillus neapolitanus c2 uid31049	CP001801.1	150	1559	1410
Helicobacter acinonychis Sheeba	AM260522.1	1	1350	1350
Helicobacter hepaticus	AE017125.1	1080619	1081989	1371
Helicobacter mustelae 12198 uid40677	FN555004.1	1	1290	1290
Helicobacter pylori 26695	AE000511.1	1607624	1608997	1374
Helicobacter pylori 51 uid9627	CP000012.1	1506576	1507943	1368
Helicobacter pylori B38 uid39685	FM991728.1	1488636	1490003	1368
Helicobacter pylori G27	CP001173.1	1571935	1573302	1368
Helicobacter pylori HPAG1	CP000241.1	1435690	1437057	1368
Helicobacter pylori J99	AE001439.1	1557789	1559162	1374
Helicobacter pylori P12	CP001217.1	1592687	1594054	1368
Helicobacter pylori Shi470	CP001072.2	1527250	1528617	1368
Helicobacter pylori uid39507	CP001680.1	1485277	1486644	1368
Heliobacterium modesticaldum Ice1	CP000930.2	1088217	1089548	1332
Herminiimonas arsenicoxydans	CU207211.1	278	1660	1383
Herpetosiphon aurantiacus ATCC 23779	CP000875.1	77	1495	1419
Hirschia baltica ATCC 49814	CP001678.1	3337218	3338720	1503
Hydrogenobacter thermophilus TK 6 uid34131	AP011112.1	100	1299	1200
Hydrogenobaculum Y04AAS1	CP001130.1	265	1599	1335
Hyphomonas neptunium ATCC 15444	CP000158.1	561363	562736	1374
Idiomarina loihiensis L2TR	AE017340.1	362	1726	1365
Jannaschia CCS1	CP000264.1	48	1475	1428
Janthinobacterium Marseille	CP000269.1	1	1383	1383
Jonesia denitrificans DSM 20603	CP001706.1	159	1589	1431
Kangiella koreensis DSM 16069	CP001707.1	363	1748	1386
Kineococcus radiotolerans SRS30216	CP000750.2	2318319	2319875	1557
Klebsiella pneumoniae 342	CP000964.1	19	1422	1404
Klebsiella pneumoniae MGH 78578	CP000647.1	4495248	4496582	1335
Klebsiella pneumoniae NTUH K2044	AP006725.1	5212742	5214145	1404
Klebsiella variicola At 22 uid37701	CP001891.1	3051	4454	1404

Kocuria rhizophila DC2201	AP009152.1	1	1695	1695
Kosmotoga olearia TBF 19 5 1	CP001634.1	103	1446	1344
Kribbella flavida DSM 17836 uid21089	CP001736.1	342	2156	1815
Kytococcus sedentarius DSM 20547	CP001686.1	209	1729	1521
Lactobacillus acidophilus NCFM	CP000033.3	31	1398	1368
Lactobacillus brevis ATCC 367	CP000416.1	113	1471	1359
Lactobacillus casei	CP000423.1	81	1430	1350
Lactobacillus casei ATCC 334	CP000423.1	81	1430	1350
Lactobacillus crispatus ST1 uid46813	FN692037.1	1	1368	1368
Lactobacillus delbrueckii bulgaricus	CR954253.1	323	1687	1365
Lactobacillus delbrueckii bulgaricus ATCC BAA-365	CP000412.1	104	1468	1365
Lactobacillus fermentum IFO 3956	AP008937.1	1	1317	1317
Lactobacillus gasseri ATCC 33323	CP000413.1	102	1496	1395
Lactobacillus helveticus DPC 4571	CP000517.1	187	1554	1368
Lactobacillus johnsonii FI9785 uid36575	FN298497.1	1	1365	1365
Lactobacillus johnsonii NCC 533	AE017198.1	1	1365	1365
Lactobacillus plantarum	CP001617.1	1	1368	1368
Lactobacillus plantarum JDM1	CP001617.1	1	1368	1368
Lactobacillus reuteri DSM 20016	CP000705.1	381	1703	1323
Lactobacillus reuteri F275 Kitasato	AP007281.1	1	1323	1323
Lactobacillus rhamnosus GG	AP011548.1	1	1350	1350
Lactobacillus rhamnosus GG uid40637	AP011548.1	1	1350	1350
Lactobacillus rhamnosus Lc 705	FM179323.1	1	1350	1350
Lactobacillus sakei 23K	CR936503.1	210	1556	1347
Lactobacillus salivarius UCC118	CP000233.1	1	1365	1365
Lactococcus lactis	AM406671.1	358	1725	1368
Lactococcus lactis cremoris MG1363	AM406671.1	1	1365	1365
Lactococcus lactis cremoris SK11	CP000425.1	144	1508	1365
Lactococcus lactis KF147 uid41115	CP001834.1	575	1942	1368
Laribacter hongkongensis HLHK9	CP001154.1	3146262	3147707	1446

Legionella longbeachae NSW150 uid39579	FN650140.1	203	1579	1377
Legionella pneumophila 2300 99 Alcoy uid18743	CP001828.1	204	1562	1359
Legionella pneumophila Corby	CP000675.2	655	2013	1359
Legionella pneumophila Lens	CR628337.1	204	1562	1359
Legionella pneumophila Paris	CR628336.1	204	1562	1359
Legionella pneumophila Philadelphia 1	AE017354.1	654	2012	1359
Leifsonia xyli xyli CTCB0	AE016822.1	225	1646	1422
Leptospira biflexa serovar Patoc Patoc 1 Ames	CP000777.1	4188	5513	1326
Leptospira biflexa serovar Patoc Patoc 1 Paris	CP000786.1	141	1466	1326
Leptospira borgpetersenii serovar Hardjo-bovis JB197	CP000350.1	4254	5567	1314
Leptospira borgpetersenii serovar Hardjo-bovis L550	CP000348.1	4254	5567	1314
Leptospira interrogans serovar Copenhageni	AE016823.1	292	1623	1332
Leptospira interrogans serovar Lai	AE010300.2	234	1565	1332
Leptothrix cholodnii SP 6	CP001013.1	72	1604	1533
Leptotrichia buccalis DSM 1135	CP001685.1	687	2042	1356
Leuconostoc citreum KM20	DQ489736.1	347	1693	1347
Leuconostoc kimchii IMSNU11154 uid40837	CP001758.1	593324	594670	1347
Leuconostoc mesenteroides ATCC 8293	CP000414.1	116	1462	1347
Listeria innocua	AL592022.1	319	1674	1356
Listeria monocytogenes	AL591824.1	305	1673	1369
Listeria monocytogenes 4b F2365	AE017262.2	319	1674	1356
Listeria monocytogenes 8 5923 uid36363	CP001604.1	2997382	2998737	1356
Listeria monocytogenes Clip81459	FM242711.1	319	1674	1356
Listeria monocytogenes HCC23	CP001175.1	2652349	2653704	1356
Listeria monocytogenes uid36361	CP001602.1	3030616	3031971	1356
Listeria seeligeri serovar 1 2b SLCC3954 uid41123	FN557490.1	319	1674	1356
Listeria welshimeri serovar 6b SLCC5334	AM263198.1	318	1673	1356
Lysinibacillus sphaericus C3 41[a]	CP000817.1	4639741	1198	-5E+06
Macrococcus caseolyticus JCSC5402	AP009484.1	348	1685	1338
Magnetococcus MC-1	CP000471.1	181	1524	1344

Magnetospirillum magneticum AMB-1	AP007255.1	683815	685248	1434
Mannheimia succiniciproducens MBEL55E	AE016827.1	447961	449334	1374
Maricaulis maris MCS10	CP000449.1	14	1462	1449
Marinobacter aquaeolei VT8	CP000514.1	466	1929	1464
Marinomonas MWYL1	CP000749.1	262	1812	1551
Meiothermus ruber DSM 1279 uid28827	CP001743.1	37	1356	1320
Mesoplasma florum L1	AE017263.1	1	1332	1332
Mesorhizobium BNC1	CP000390.1	85	1524	1440
Mesorhizobium loti	BA000012.4	4477398	4478942	1545
Methylibium petroleiphilum PM1	CP000555.1	186	1589	1404
Methylobacillus flagellatus KT	CP000284.1	46	1458	1413
Methylobacterium 4 46	CP000943.1	176	1660	1485
Methylobacterium chloromethanicum CM4	CP001298.1	1438	2943	1506
Methylobacterium extorquens AM1	CP001510.1	712	2217	1506
Methylobacterium extorquens DM4	FP103042.2	710	2215	1506
Methylobacterium extorquens PA1	CP000908.1	44	1549	1506
Methylobacterium nodulans ORS 2060	CP001349.1	1	1500	1500
Methylobacterium populi BJ001	CP001029.1	1161	2666	1506
Methylobacterium radiotolerans JCM 2831	CP001001.1	769	2265	1497
Methylocella silvestris BL2	CP001280.1	362	1882	1521
Methylococcus capsulatus Bath	AE017282.2	3219779	3221107	1329
Methylotenera 301 uid39983	CP002056.1	94	1509	1416
Methylotenera mobilis JLW8	CP001672.1	202	1620	1419
Methylovorus SIP3 4	CP001674.1	138	1547	1410
Micrococcus luteus NCTC 2665	CP001628.1	256	1803	1548
Microcystis aeruginosa NIES 843	AP009552.1	2048563	2049897	1335
Moorella thermoacetica ATCC 39073	CP000232.1	450	1778	1329
Moraxella catarrhalis RH4 uid46869	CP002005.1	993	2396	1404
Mycobacterium abscessus ATCC 19977	CU458896.1	1	1476	1476
Mycobacterium avium 104	CP000479.1	33	1529	1497

Mycobacterium avium paratuberculosis	AE016958.1	1	1530	1530
Mycobacterium bovis	BX248333.1	1	1524	1524
Mycobacterium bovis BCG Tokyo 172	AP010918.1	1	1524	1524
Mycobacterium gilvum PYR-GCK	CP000656.1	858447	859925	1479
Mycobacterium JLS	CP000580.1	252	1739	1488
Mycobacterium KMS	CP000518.1	5935	7422	1488
Mycobacterium leprae	AL450380.1	1	1566	1566
Mycobacterium leprae Br4923	FM211192.1	1	1566	1566
Mycobacterium marinum M	CP000854.1	1	1533	1533
Mycobacterium MCS	CP000384.1	20	1507	1488
Mycobacterium smegmatis MC2 155	CP000480.1	6986600	6988114	1515
Mycobacterium tuberculosis CDC1551	AE000516.2	1	1524	1524
Mycobacterium tuberculosis F11	CP000717.1	102	1625	1524
Mycobacterium tuberculosis H37Ra	CP000611.1	1	1524	1524
Mycobacterium tuberculosis H37Rv	AL123456.2	1	1524	1524
Mycobacterium tuberculosis KZN 1435	CP001658.1	103	1626	1524
Mycobacterium ulcerans Agy99	CP000325.1	1	1533	1533
Mycobacterium vanbaalenii PYR-1	CP000511.1	249	1733	1485
Mycoplasma agalactiae PG2	CU179680.1	1	1401	1401
Mycoplasma arthritidis 158L3 1	CP001047.1	107	1471	1365
Mycoplasma capricolum ATCC 27343	CP000123.1	1	1353	1353
Mycoplasma conjunctivae HRC 581 uid32285	FM864216.2	101	1513	1413
Mycoplasma crocodyli MP145 uid29021	CP001991.1	1	1305	1305
Mycoplasma gallisepticum	CP001873.1	3163	4548	1386
Mycoplasma gallisepticum F uid43301	CP001873.1	3159	4544	1386
Mycoplasma gallisepticum R high uid43299	CP001872.1	3163	4548	1386
Mycoplasma genitalium	L43967.2	577268	578581	1314
Mycoplasma hyopneumoniae 232	AE017332.1	1	1392	1392
Mycoplasma hyopneumoniae 7448	AE017244.1	207	1598	1392
Mycoplasma hyopneumoniae J	AE017243.1	207	1598	1392

Mycoplasma mobile 163K	AE017308.1	215	1603	1389
Mycoplasma mycoides	BX293980.2	1	1440	1440
Mycoplasma mycoides capri GM12 uid39245	CP001668.1	1	1353	1353
Mycoplasma penetrans	BA000026.2	1	1359	1359
Mycoplasma pneumoniae	U00089.2	813468	814787	1320
Mycoplasma pulmonis	AL445566.1	222	1607	1386
Mycoplasma synoviae 53	AE017245.1	57	1427	1371
Myxococcus xanthus DK 1622	CP000113.1	87	1439	1353
Nakamurella multipartita DSM 44233	CP001737.1	176	1849	1674
Natranaerobius thermophilus JW NM WN LF	CP001034.1	146	1507	1362
Nautilia profundicola AmH	CP001279.1	101	1411	1311
Neisseria gonorrhoeae FA 1090	AE004969.1	159	1715	1557
Neisseria gonorrhoeae NCCP11945	CP001050.1	206	1714	1509
Neisseria meningitidis 53442	CP000381.1	321373	322779	1407
Neisseria meningitidis 8013 uid34687	FM999788.1	2010063	2011619	1557
Neisseria meningitidis alpha14	AM889136.1	281876	283432	1557
Neisseria meningitidis FAM18	AM421808.1	321483	323039	1557
Neisseria meningitidis MC58	AE002098.2	2004389	2005945	1557
Neisseria meningitidis Z2491	AL157959.1	527602	529158	1557
Neorickettsia risticii Illinois	CP001431.1	231140	232936	1797
Neorickettsia sennetsu Miyayama	CP000237.1	220315	221739	1425
Nitratiruptor SB155-2	AP009178.1	139	1470	1332
Nitrobacter hamburgensis X14	CP000319.1	107	1534	1428
Nitrobacter winogradskyi Nb-255	CP000115.1	567	1994	1428
Nitrosococcus halophilus Nc4 uid36589	CP001798.1	244	1596	1353
Nitrosococcus oceani ATCC 19707	CP000127.1	175	1524	1350
Nitrosomonas europaea	AL954747.1	211	1590	1380
Nitrosomonas eutropha C71	CP000450.1	35	1414	1380
Nitrosospira multiformis ATCC 25196	CP000103.1	69	1499	1431
Nocardia farcinica IFM10152	AP006618.1	1	1989	1989

Nocardioides JS614	CP000509.1	72	1658	1587
Nostoc punctiforme PCC 73102	CP001037.1	167	1546	1380
Nostoc sp	BA000019.2	2403018	2404397	1380
Novosphingobium aromaticivorans DSM 12444	CP000248.1	24	1520	1497
Oceanobacillus iheyensis	BA000028.3	300	1643	1344
Ochrobactrum anthropi ATCC 49188	CP000758.1	1439	3001	1563
Oenococcus oeni PSU-1	CP000411.1	1	1353	1353
Oligotropha carboxidovorans OM5	CP001196.1	527180	528613	1434
Opitutus terrae PB90 1	CP001032.1	20	1423	1404
Orientia tsutsugamushi Boryong	AM494475.1	22	1431	1410
Orientia tsutsugamushi Ikeda	AP008981.1	1601042	1602454	1413
Paenibacillus JDR 2	CP001656.1	27	1379	1353
Pantoea ananatis LMG 20103 uid43085	CP001875.1	95034	96257	1224
Parabacteroides distasonis ATCC 8503	CP000140.1	1	1398	1398
Paracoccus denitrificans PD1222	CP000490.1	405	1751	1347
Parvibaculum lavamentivorans DS-1	CP000774.1	1568	3127	1560
Pasteurella multocida	AE004439.1	1357003	1358358	1356
Pectobacterium carotovorum PC1	CP001657.1	72	1469	1398
Pectobacterium wasabiae WPP163 uid31293	CP001790.1	53	1450	1398
Pediococcus pentosaceus ATCC 25745	CP000422.1	50	1390	1341
Pelobacter carbinolicus	CP000142.2	72	1430	1359
Pelobacter propionicus DSM 2379	CP000482.1	1	1353	1353
Pelodictyon phaeoclathratiforme BU 1	CP001110.1	1	1464	1464
Pelotomaculum thermopropionicum SI	AP009389.1	1	1344	1344
Persephonella marina EX H1	CP001230.1	867511	868824	1314
Petrotoga mobilis SJ95	CP000879.1	53	1417	1365
Phenylobacterium zucineum HLK1	CP000747.1	3882661	3884115	1455
Photobacterium profundum SS9	CR354531.1	7364	8788	1425
Photorhabdus asymbiotica	FM162591.1	133	1452	1320
Photorhabdus luminescens	BX470251.1	234	1622	1389

Planctomyces limnophilus DSM 3776 uid29411	CP001744.1	614	3166	2553
Polaromonas JS666	CP000316.1	40	1434	1395
Polaromonas naphthalenivorans CJ2	CP000529.1	4402850	4404247	1398
Polynucleobacter necessarius asymbioticus QLW P1DMWA 1	CP000655.1	33	1460	1428
Polynucleobacter necessarius STIR1	CP001010.1	113	1537	1425
Porphyromonas gingivalis ATCC 33277	AP009380.1	1	1422	1422
Porphyromonas gingivalis W83	AE015924.1	1	1422	1422
Prevotella ruminicola 23 uid10619	CP002006.1	1529107	1530528	1422
Prochlorococcus marinus AS9601	CP000551.1	541765	543159	1395
Prochlorococcus marinus CCMP1375	AE017126.1	537957	539342	1386
Prochlorococcus marinus MED4	BX548174.1	531716	533107	1392
Prochlorococcus marinus MIT 9211	CP000878.1	530649	532013	1365
Prochlorococcus marinus MIT 9215	CP000825.1	567695	569089	1395
Prochlorococcus marinus MIT 9301	CP000576.1	516187	517581	1395
Prochlorococcus marinus MIT 9303	CP000554.1	755354	756745	1392
Prochlorococcus marinus MIT 9312	CP000111.1	526327	527721	1395
Prochlorococcus marinus MIT 9515	CP000552.1	562236	563627	1392
Prochlorococcus marinus MIT9313	BX548175.1	1277265	1278662	1398
Prochlorococcus marinus NATL1A	CP000553.1	562863	564257	1395
Prochlorococcus marinus NATL2A	CP000095.2	550495	551895	1401
Propionibacterium acnes KPA171202	AE017283.1	245	1747	1503
Propionibacterium acnes SK137 uid31005	CP001977.1	241	1743	1503
Prosthecochloris aestuarii DSM 271	CP001108.1	1	1479	1479
Prosthecochloris vibrioformis DSM 265	CP000607.1	1	1464	1464
Proteus mirabilis	AM942759.1	3445820	3447220	1401
Pseudoalteromonas atlantica T6c	CP000388.1	96	1520	1425
Pseudoalteromonas haloplanktis TAC125	CR954246.1	118	1542	1425
Pseudomonas aeruginosa	FM209186.1	483	2027	1545
Pseudomonas aeruginosa LESB58	FM209186.1	483	2027	1545
Pseudomonas aeruginosa PA7	CP000744.1	488	2026	1539

Pseudomonas aeruginosa UCBPP-PA14	CP000438.1	483	2027	1545
Pseudomonas entomophila L48	CT573326.1	552	2078	1527
Pseudomonas fluorescens Pf0 1	CP000094.2	565	2088	1524
Pseudomonas fluorescens Pf-5	CP000076.1	101	1642	1542
Pseudomonas fluorescens SBW25	AM181176.4	1	1506	1506
Pseudomonas mendocina ymp	CP000680.1	82	1566	1485
Pseudomonas putida F1	CP000712.1	385	1902	1518
Pseudomonas putida GB 1	CP000926.1	1152	2684	1533
Pseudomonas putida KT2440	AE015451.1	9542	11062	1521
Pseudomonas putida W619	CP000949.1	386	1921	1536
Pseudomonas stutzeri A1501	CP000304.1	380	1924	1545
Pseudomonas syringae phaseolicola 1448A	CP000058.1	238	1773	1536
Pseudomonas syringae pv B728a	CP000075.1	1	1536	1536
Pseudomonas syringae tomato DC3000	AE016853.1	339	1874	1536
Psychrobacter arcticum 273-4	CP000082.1	520	1965	1446
Psychrobacter cryohalolentis K5	CP000323.1	1365	2810	1446
Psychrobacter PRwf-1	CP000713.1	1311	2723	1413
Psychromonas ingrahamii 37	CP000510.1	4524210	4525586	1377
Ralstonia eutropha H16	AM260479.1	689	2455	1767
Ralstonia eutropha JMP134	CP000090.1	184	2019	1836
Ralstonia pickettii 12D	CP001644.1	613	2202	1590
Ralstonia pickettii 12J	CP001068.1	328	1917	1590
Ralstonia solanacearum	AL646052.1	3714708	3716276	1569
Renibacterium salmoninarum ATCC 33209	CP000910.1	144	1547	1404
Rhizobium etli CFN 42	CP000133.1	370494	372044	1551
Rhizobium etli CIAT 652	CP001074.1	406075	407625	1551
Rhizobium leguminosarum bv trifolii WSM1325	CP001622.1	55	1605	1551
Rhizobium leguminosarum bv trifolii WSM2304	CP001191.1	620	2170	1551
Rhizobium leguminosarum bv viciae 3841	AM236080.1	410392	411840	1449
Rhizobium NGR234	CP001389.1	202	1644	1443

Rhodobacter capsulatus SB1003 uid55	CP001312.1	351	1724	1374
Rhodobacter sphaeroides 2 4 1	CP000143.1	3110775	3112142	1368
Rhodobacter sphaeroides ATCC 17025	CP000661.1	842	2227	1386
Rhodobacter sphaeroides ATCC 17029	CP000577.1	9930	11297	1368
Rhodobacter sphaeroides KD131	CP001150.1	2784388	2785731	1344
Rhodococcus erythropolis PR4	AP008957.1	768	2330	1563
Rhodococcus jostii RHA1	CP000431.1	3872441	3874027	1587
Rhodococcus opacus B4 uid34839	AP011115.1	3776104	3777690	1587
Rhodoferax ferrireducens T118	CP000267.1	7	1419	1413
Rhodopseudomonas palustris BisA53	CP000463.1	641	2065	1425
Rhodopseudomonas palustris BisB18	CP000301.1	758	2179	1422
Rhodopseudomonas palustris BisB5	CP000283.1	649	2067	1419
Rhodopseudomonas palustris CGA009	BX571963.1	679	2097	1419
Rhodopseudomonas palustris HaA2	CP000250.1	676	2094	1419
Rhodopseudomonas palustris TIE 1	CP001096.1	220	1638	1419
Rhodospirillum centenum SW	CP000613.2	3127693	3129216	1524
Rhodospirillum rubrum ATCC 11170	CP000230.1	228	1757	1530
Rhodothermus marinus DSM 4252 uid29281	CP001807.1	165	1676	1512
Rickettsia africae ESF 5	CP001612.1	871344	872735	1392
Rickettsia akari Hartford	CP000847.1	846716	848107	1392
Rickettsia bellii OSU 85-389	CP000849.1	498632	500023	1392
Rickettsia bellii RML369-C	CP000087.1	932808	934199	1392
Rickettsia canadensis McKiel	CP000409.1	404050	405441	1392
Rickettsia conorii	AE006914.1	862914	864305	1392
Rickettsia felis URRWXCal2	CP000053.1	382683	384074	1392
Rickettsia massiliae MTU5	CP000683.1	945604	946995	1392
Rickettsia peacockii Rustic	CP001227.1	694602	695993	1392
Rickettsia prowazekii	CP001584.1	756866	758257	1392
Rickettsia prowazekii Rp22 uid19813	CP001584.1	756779	758170	1392
Rickettsia rickettsii Iowa	CP000766.1	866869	868260	1392

Rickettsia rickettsii Sheila Smith	CP000848.1	855505	856896	1392
Rickettsia typhi wilmington	AE017197.1	762664	764055	1392
Robiginitalea biformata HTCC2501	CP001712.1	2781545	2782969	1425
Roseiflexus castenholzii DSM 13941	CP000804.1	370	1812	1443
Roseiflexus RS-1	CP000686.1	415	1860	1446
Roseobacter denitrificans OCh 114	CP000362.1	204958	206322	1365
Rothia mucilaginosa uid38547	AP011540.1	1	1695	1695
Rubrobacter xylanophilus DSM 9941	CP000386.1	2	1354	1353
Ruegeria pomeroyi DSS 3	CP000031.1	164331	165737	1407
Saccharomonospora viridis DSM 43017	CP001683.1	99	1892	1794
Saccharophagus degradans 14642	CP000282.1	383	1957	1575
Saccharopolyspora erythraea NRRL 2338	AM420293.1	1	1779	1779
Salinispora arenicola CNS-205	CP000850.1	148	1923	1776
Salinispora tropica CNB-440	CP000667.1	625	2388	1764
Salmonella enterica arizonae serovar 62 z4 z23	CP000880.1	3740840	3742249	1410
Salmonella enterica Choleraesuis	AE017220.1	3982617	3984017	1401
Salmonella enterica Paratypi ATCC 9150	CP000026.1	3825575	3826975	1401
Salmonella enterica serovar Agona SL483	CP001138.1	3964318	3965718	1401
Salmonella enterica serovar Dublin CT 2021853	CP001144.1	4076670	4078070	1401
Salmonella enterica serovar Enteritidis P125109	AM933172.1	3918280	3919680	1401
Salmonella enterica serovar Gallinarum 287 91	AM933173.1	3779210	3780610	1401
Salmonella enterica serovar Heidelberg SL476	CP001120.1	4057833	4059233	1401
Salmonella enterica serovar Newport SL254	CP001113.1	4014207	4015607	1401
Salmonella enterica serovar Paratyphi A AKU 12601	FM200053.1	3821254	3822654	1401
Salmonella enterica serovar Paratyphi B SPB7	CP000886.1	3992516	3993916	1401
Salmonella enterica serovar Paratyphi C RKS4594	CP000857.1	3979370	3980782	1413
Salmonella enterica serovar Schwarzengrund CVM19633	CP001127.1	3923952	3925352	1401
Salmonella enterica serovar Typhi Ty2	AE014613.1	3790618	3792018	1401
Salmonella enterica serovar Typhimurium 14028S uid33067	CP001363.1	4057317	4058717	1401
Salmonella enterica serovar Typhimurium uid40625	FN424405.1	4066500	4067900	1401

Salmonella typhi	AE006468.1	3805127	3806527	1401
Salmonella typhimurium LT2	AE006468.1	4043624	4045024	1401
Sanguibacter keddieii DSM 10542 uid19711	CP001819.1	137	1609	1473
Sebaldella termitidis ATCC 33386 uid29539	CP001739.1	361	1719	1359
Segniliparus rotundus DSM 44985 uid37711	CP001958.1	130	1593	1464
Serratia proteamaculans 568	CP000826.1	30606	31997	1392
Shewanella amazonensis SB2B	CP000507.1	10775	12148	1374
Shewanella ANA-3	CP000469.1	9651	11033	1383
Shewanella baltica OS155	CP000563.1	41	1429	1389
Shewanella baltica OS185	CP000753.1	382	1770	1389
Shewanella baltica OS195	CP000891.1	382	1770	1389
Shewanella baltica OS223	CP001252.1	77	1465	1389
Shewanella denitrificans OS217	CP000302.1	10	1398	1389
Shewanella frigidimarina NCIMB 400	CP000447.1	23	1408	1386
Shewanella halifaxensis HAW EB4	CP000931.1	81	1466	1386
Shewanella loihica PV-4	CP000606.1	111	1490	1380
Shewanella MR-4	CP000446.1	79	1461	1383
Shewanella MR-7	CP000444.1	81	1463	1383
Shewanella oneidensis	AE014299.1	6873	8255	1383
Shewanella pealeana ATCC 700345	CP000851.1	223	1608	1386
Shewanella piezotolerans WP3	CP000472.1	16217	17602	1386
Shewanella putrefaciens CN-32	CP000681.1	147	1532	1386
Shewanella sediminis HAW-EB3	CP000821.1	4812	6200	1389
Shewanella violacea DSS12 uid34739	AP011177.1	30205	31593	1389
Shewanella W3-18-1	CP000503.1	150	1535	1386
Shewanella woodyi ATCC 51908	CP000961.1	397	1785	1389
Shigella boydii CDC 3083 94	CP001063.1	3946927	3948330	1404
Shigella boydii Sb227	CP000036.1	3689193	3690596	1404
Shigella dysenteriae	CP000034.1	3918205	3919608	1404
Shigella flexneri 2002017 uid33639	CP001383.1	3893394	3894797	1404

Shigella flexneri 2a	AE014073.1	3869099	3870325	1227
Shigella flexneri 2a 2457T	AE014073.1	3904162	3905565	1404
Shigella flexneri 5 8401	CP000266.1	3903225	3904556	1332
Shigella sonnei Ss046	CP000038.1	3823237	3824640	1404
Sideroxydans lithotrophicus ES 1 uid33161	CP001965.1	72	1415	1344
Silicibacter TM1040	CP000377.1	45	1457	1413
Sinorhizobium medicae WSM419	CP000738.1	1405	2850	1446
Sinorhizobium meliloti	AL591688.1	399330	400853	1524
Slackia heliotrinireducens DSM 20476	CP001684.1	42	1547	1506
Sodalis glossinidius morsitans	AP008232.1	1	1395	1395
Sorangium cellulosum So ce 56	AM746676.1	1	1416	1416
Sphaerobacter thermophilus DSM 20745 uid21087	CP001823.1	47	1462	1416
Sphingobium japonicum UT26S uid19949	AP010803.1	835602	837011	1410
Sphingomonas wittichii RW1	CP000699.1	245	1678	1434
Sphingopyxis alaskensis RB2256	CP000356.1	173	1534	1362
Spirosoma linguale DSM 74 uid28817	CP001769.1	60	1469	1410
Stackebrandtia nassauensis DSM 44728 uid19713	CP001778.1	64	1764	1701
Staphylococcus aureus 4 2981 uid34809	CP001844.1	517	1878	1362
Staphylococcus aureus aureus MRSA252	BX571856.1	517	1878	1362
Staphylococcus aureus aureus MSSA476	BX571857.1	517	1878	1362
Staphylococcus aureus COL	CP000046.1	544	1905	1362
Staphylococcus aureus ED98 uid39547	CP001781.1	517	1878	1362
Staphylococcus aureus JH1	CP000736.1	641	2002	1362
Staphylococcus aureus JH9	CP000703.1	572	1933	1362
Staphylococcus aureus Mu3	AP009324.1	517	1878	1362
Staphylococcus aureus Mu50	BA000017.4	517	1878	1362
Staphylococcus aureus MW2	BA000033.2	517	1878	1362
Staphylococcus aureus N315	BA000018.3	517	1878	1362
Staphylococcus aureus NCTC 8325	CP000253.1	517	1878	1362
Staphylococcus aureus Newman	AP009351.1	517	1878	1362

Staphylococcus aureus RF122	AJ938182.1	517	1878	1362
Staphylococcus aureus ST398 uid29427	AM990992.1	517	1878	1362
Staphylococcus aureus TW20 uid36647	FN433596.1	517	1878	1362
Staphylococcus aureus USA300 FPR3757	CP000255.1	544	1905	1362
Staphylococcus aureus USA300 TCH1516	CP000730.1	544	1905	1362
Staphylococcus carnosus TM300	AM295250.1	2564508	2565869	1362
Staphylococcus epidermidis ATCC 12228	AE015929.1	362	1717	1356
Staphylococcus epidermidis RP62A	CP000029.1	2614976	2616331	1356
Staphylococcus haemolyticus	AP006716.1	508	1863	1356
Staphylococcus lugdunensis HKU09 1 uid42395	CP001837.1	2625430	2626797	1368
Staphylococcus saprophyticus	AP008934.1	509	1876	1368
Stenotrophomonas maltophilia K279a	AM743169.1	1	1332	1332
Stenotrophomonas maltophilia R551 3	CP001111.1	215	1546	1332
Streptobacillus moniliformis DSM 12112 uid29309	CP001779.1	66	1391	1326
Streptococcus agalactiae 2603	AE009948.1	102	1463	1362
Streptococcus agalactiae A909	CP000114.1	101	1462	1362
Streptococcus agalactiae NEM316	AL732656.1	176	1537	1362
Streptococcus dysgalactiae equisimilis GGS 124	AP010935.1	225	1580	1356
Streptococcus equi 4047	FM204883.1	1	1353	1353
Streptococcus equi zooepidemicus	FM204884.1	1	1353	1353
Streptococcus equi zooepidemicus MGCS10565	CP001129.1	233	1585	1353
Streptococcus gallolyticus UCN34 uid34729	FN597254.1	170	1525	1356
Streptococcus gordonii Challis substr CH1	CP000725.1	166	1518	1353
Streptococcus mitis B6 uid16302	FN568063.1	1	1362	1362
Streptococcus mutans	AE014133.1	194	1552	1359
Streptococcus mutans NN2025 uid28997	AP010655.1	194	1552	1359
Streptococcus pneumoniae 70585	CP000918.1	197	1558	1362
Streptococcus pneumoniae ATCC 700669	FM211187.1	186	1547	1362
Streptococcus pneumoniae CGSP14	CP001033.1	197	1558	1362
Streptococcus pneumoniae D39	CP000410.1	1	1362	1362

Streptococcus pneumoniae G54	CP001015.1	197	1558	1362
Streptococcus pneumoniae Hungary19A 6	CP000936.1	197	1558	1362
Streptococcus pneumoniae JJA	CP000919.1	197	1558	1362
Streptococcus pneumoniae P1031	CP000920.1	197	1558	1362
Streptococcus pneumoniae R6	AE007317.1	1	1362	1362
Streptococcus pneumoniae Taiwan19F 14	CP000921.1	197	1558	1362
Streptococcus pneumoniae TIGR4	AE005672.3	197	1558	1362
Streptococcus pyogenes M1 GAS	AE004092.1	232	1587	1356
Streptococcus pyogenes Manfredo	AM295007.1	202	1557	1356
Streptococcus pyogenes MGAS10270	CP000260.1	202	1557	1356
Streptococcus pyogenes MGAS10394	CP000003.1	202	1557	1356
Streptococcus pyogenes MGAS10750	CP000262.1	202	1557	1356
Streptococcus pyogenes MGAS2096	CP000261.1	202	1557	1356
Streptococcus pyogenes MGAS315	AE014074.1	232	1587	1356
Streptococcus pyogenes MGAS5005	CP000017.1	202	1557	1356
Streptococcus pyogenes MGAS6180	CP000056.1	202	1557	1356
Streptococcus pyogenes MGAS8232	AE009949.1	202	1557	1356
Streptococcus pyogenes MGAS9429	CP000259.1	202	1557	1356
Streptococcus pyogenes NZ131	CP000829.1	232	1587	1356
Streptococcus pyogenes SSI-1	BA000034.2	232	1587	1356
Streptococcus sanguinis SK36	CP000387.1	214	1566	1353
Streptococcus suis 05ZYH33	CP000407.1	1	1374	1374
Streptococcus suis 98HAH33	CP000408.1	1	1374	1374
Streptococcus suis BM407	FM252032.1	38	1372	1335
Streptococcus suis GZ1 uid18737	CP000837.1	1	1374	1374
Streptococcus suis P1 7 uid352	AM946016.1	1	1374	1374
Streptococcus suis SC84	FM252031.1	1	1374	1374
Streptococcus thermophilus CNRZ1066	CP000024.1	186	1550	1365
Streptococcus thermophilus LMD-9	CP000419.1	101	1465	1365
Streptococcus thermophilus LMG 18311	CP000023.1	186	1550	1365

Streptococcus uberis 0140J	AM946015.1	1	1356	1356
Streptomyces avermitilis	BA000030.3	5285972	5287933	1962
Streptomyces bingchenggensis BCW 1 uid46847	CP002047.1	6602155	6604011	1857
Streptomyces coelicolor	AL645882.2	4270778	4272748	1971
Streptomyces griseus NBRC 13350	AP009493.1	4322502	4324370	1869
Streptomyces scabiei 87 22 uid40749	FN554889.1	5139761	5141785	2025
Streptosporangium roseum DSM 43021 uid21083	CP001814.1	361	2109	1749
Sulfurihydrogenibium azorense Az Fu1	CP001229.1	696482	697330	849
Sulfurihydrogenibium YO3AOP1	CP001080.1	129	1508	1380
Sulfurospirillum deleyianum DSM 6946 uid29529	CP001816.1	1277	2602	1326
Sulfurovum NBC37-1	AP009179.1	65	1393	1329
Symbiobacterium thermophilum IAM14863	AP006840.1	1	1377	1377
Synechococcus CC9311	CP000435.1	1723679	1725085	1407
Synechococcus CC9605	CP000110.1	927763	929115	1353
Synechococcus CC9902	CP000097.1	881683	883071	1389
Synechococcus elongatus PCC 6301	AP008231.1	502252	503700	1449
Synechococcus elongatus PCC 7942	CP000100.1	1117103	1118551	1449
Synechococcus PCC 7002	CP000951.1	1	1350	1350
Synechococcus RCC307	CT978603.1	756450	757835	1386
Synechococcus sp WH8102	BX548020.1	1479573	1480970	1398
Synechococcus WH 7803	CT971583.1	697164	698558	1395
Synechocystis PCC6803	BA000022.2	1350236	1351579	1344
Syntrophobacter fumaroxidans MPOB	CP000478.1	346	1698	1353
Syntrophomonas wolfei Goettingen	CP000448.1	83	1393	1311
Syntrophothermus lipocalidus DSM 12680 uid37873	CP002048.1	108	1418	1311
Syntrophus aciditrophicus SB	CP000252.1	155	1528	1374
Teredinibacter turnerae T7901	CP001614.2	541	2322	1782
Thauera MZ1T	CP001281.2	250	1752	1503
Thermanaerovibrio acidaminovorans DSM 6589 uid29531	CP001818.1	97	1440	1344
Thermoanaerobacter italicus Ab9 uid33157	CP001936.1	424	1755	1332

Thermoanaerobacter mathranii A3 uid33329	CP002032.1	99	1430	1332
Thermoanaerobacter pseudethanolicus ATCC 33223	CP000924.1	79	1410	1332
Thermoanaerobacter tengcongensis	AE008691.1	365	1696	1332
Thermoanaerobacter X514	CP000923.1	195	1526	1332
Thermobaculum terrenum ATCC BAA 798 uid29523	CP001825.1	85	1455	1371
Thermobifida fusca YX	CP000088.1	195	2042	1848
Thermobispora bispora DSM 43833 uid20737	CP001874.1	270	1853	1584
Thermocrinis albus DSM 14484 uid37275	CP001931.1	164163	165347	1185
Thermodesulfovibrio yellowstonii DSM 11347	CP001147.1	13985	15301	1317
Thermomicrobium roseum DSM 5159	CP001275.1	1157859	1159256	1398
Thermomonospora curvata DSM 43183 uid20825	CP001738.1	61	2226	2166
Thermosipho africanus TCF52B	CP001185.1	219924	221240	1317
Thermosipho melanesiensis BI429	CP000716.1	162	1475	1314
Thermosynechococcus elongatus	BA000039.2	609990	611351	1362
Thermotoga lettingae TMO	CP000812.1	137	1471	1335
Thermotoga maritima	AE000512.1	943332	944273	942
Thermotoga naphthophila RKU 10 uid33663	CP001839.1	38	1360	1323
Thermotoga neapolitana DSM 4359	CP000916.1	1617713	1619065	1353
Thermotoga petrophila RKU-1	CP000702.1	72	1394	1323
Thermotoga RQ2	CP000969.1	62	1384	1323
Thermus thermophilus HB27	AE017221.1	1523000	1524340	1341
Thermus thermophilus HB8	AP008226.1	1848185	1849495	1311
Thioalkalivibrio HL EbGR7	CP001339.1	139	1506	1368
Thioalkalivibrio K90mix uid30759	CP001905.1	118	1485	1368
Thiobacillus denitrificans ATCC 25259	CP000116.1	137	1510	1374
Thiomicrospira crunogena XCL-2	CP000109.2	1	1404	1404
Thiomicrospira denitrificans ATCC 33889	CP000153.1	15	1322	1308
Thiomonas intermedia K12 uid33641	CP002021.1	433	1887	1455
Tolumonas auensis DSM 9187	CP001616.1	12	1397	1386
Treponema denticola ATCC 35405	AE017226.1	166	1575	1410

Treponema pallidum	CP001752.1	4	1398	1395
Treponema pallidum Chicago uid39981	CP001752.1	4	1398	1395
Treponema pallidum SS14	CP000805.1	4	1398	1395
Trichodesmium erythraeum IMS101	CP000393.1	27	1397	1371
Tropheryma whipplei TW08 27	BX072543.1	1	1437	1437
Tropheryma whipplei Twist	AE014184.1	1	1437	1437
Truepera radiovictrix DSM 17093 uid38371	CP002049.1	166	1500	1335
Tsukamurella paurometabola DSM 20162 uid29399	CP001966.1	190	1662	1473
Ureaplasma parvum serovar 3 ATCC 27815	CP000942.1	27	1400	1374
Ureaplasma urealyticum	CP001184.1	1	1374	1374
Ureaplasma urealyticum serovar 10 ATCC 33699	CP001184.1	17	1390	1374
Variovorax paradoxus S110	CP001635.1	37	1416	1380
Veillonella parvula DSM 2008 uid21091	CP001820.1	248	1822	1575
Verminephrobacter eiseniae EF01-2	CP000542.1	493	1944	1452
Vibrio cholerae	CP001485.1	7397	8815	1419
Vibrio cholerae M66 2	CP001233.1	7397	8815	1419
Vibrio cholerae MJ 1236	CP001485.1	517742	519145	1404
Vibrio cholerae O395	CP001235.1	2660512	2661930	1419
Vibrio cholerae O395 uid32853	CP001235.1	167860	169278	1419
Vibrio Ex25 uid40507	CP001805.1	418744	420150	1407
Vibrio fischeri ES114	CP000020.2	7401	8810	1410
Vibrio fischeri MJ11	CP001139.1	7492	8832	1341
Vibrio harveyi ATCC BAA-1116	CP000789.1	423785	425191	1407
Vibrio parahaemolyticus	BA000031.2	7680	9086	1407
Vibrio splendidus LGP32	FM954972.2	7498	8919	1422
Vibrio vulnificus YJ016	BA000037.2	7450	8856	1407
Wolbachia endosymbiont of Brugia malayi TRS	AE017321.1	361157	362539	1383
Wolbachia endosymbiont of Culex quinquefasciatus Pel	AM999887.1	152	1534	1383
Wolbachia endosymbiont of Drosophila melanogaster	AE017196.1	153	1535	1383
Wolbachia wRi	CP001391.1	153	1535	1383

Wolinella succinogenes	BX571656.1	1	1314	1314
Xanthobacter autotrophicus Py2	CP000781.1	330	1850	1521
Xanthomonas campestris 8004	CP000050.1	42	1370	1329
Xanthomonas campestris ATCC 33913	AE008922.1	42	1370	1329
Xanthomonas campestris B100	AM920689.1	1	1329	1329
Xanthomonas campestris vesicatoria 85-10	AM039952.1	1	1329	1329
Xanthomonas citri	AE008923.1	42	1370	1329
Xanthomonas oryzae KACC10331	AE013598.1	42	1373	1332
Xanthomonas oryzae MAFF 311018	AP008229.1	41	1369	1329
Xanthomonas oryzae PXO99A	CP000967.1	45	1373	1329
Xenorhabdus bovienii SS 2004 uid13399	FN667741.1	159	1547	1389
Xylanimonas cellulosilytica DSM 15894 uid19715	CP001821.1	150	1556	1407
Xylella fastidiosa	AE009442.1	143	1462	1320
Xylella fastidiosa M12	CP000941.1	20	1351	1332
Xylella fastidiosa M23	CP001011.1	25	1356	1332
Xylella fastidiosa Temecula1	AE009442.1	146	1465	1320
Yersinia enterocolitica 8081	AM286415.1	4572805	4574193	1389
Yersinia pestis Angola	CP000901.1	4468868	4470256	1389
Yersinia pestis Antiqua	CP000308.1	4666903	4668291	1389
Yersinia pestis biovar Microtus 91001	AE017042.1	4559438	4560826	1389
Yersinia pestis CO92	AL590842.1	4618341	4619729	1389
Yersinia pestis D106004 uid36507[b]	CP001585.1	4605331	4606721	1391
Yersinia pestis D182038 uid36545	CP001589.1	4591558	4592946	1389
Yersinia pestis KIM 10 uid288	AE009952.1	4565119	4566519	1401
Yersinia pestis Nepal516	CP000305.1	4499203	4500591	1389
Yersinia pestis Pestoides F	CP000668.1	34	1422	1389
Yersinia pestis Z176003 uid36547	CP001593.1	4518199	4519587	1389
Yersinia pseudotuberculosis IP 31758	CP000720.1	4688891	4690279	1389
Yersinia pseudotuberculosis IP32953	BX936398.1	4709259	4710647	1389
Yersinia pseudotuberculosis PB1	CP001048.1	37	1389	1353

Yersinia pseudotuberculosis YPIII	CP000950.1	37	1389	1353
Zunongwangia profunda SM A87 uid38641	CP001650.1	3877760	3879187	1428
Zymomonas mobilis NCIMB 11163 uid34821	CP001722.1	274	1728	1455
Zymomonas mobilis ZM4	AE008692.2	1370644	1372098	1455

Eleven strains lacking a *dnaA* gene in their chromosomes are shown first, followed by forty strains that have two copies of the *dnaA* gene (the second copy is listed after the first) and 1,016 strains that have a single copy of the *dnaA* gene. The strains lacking a *dnaA* gene are all endosymbionts of insects with reduced genomes. Strains containing two copies of the *dnaA* gene are pathogens of animals, extremophiles and environmental cultures from water. All plant pathogens contain a single copy of the *dnaA* gene.

[a] The *dnaA* gene of strain *Lysinibacillus sphaericus* C3 41 begins at position 4,639,741 of 4,639,821 of the circular chromosome instead of position 1.

[b] The *dnaA* gene of this strain *Yersinia pestis* D106004 contains a frameshift mutation due to a single nucleotide insertion at position 498. The *dnaA* gene was identified for this genome with bl2seq using *Yersinia pestis* Angola (CP000901.1).
