# Supplementary material for: Classification of Plant Associated Bacteria Using RIF, a Computationally Derived DNA Marker
Source: PLoS One. 2011 Apr 21;6(4):e18496. doi: 10.1371/journal.pone.0018496 (PMC3080875; doi:10.1371/journal.pone.0018496)
Supplement: Table S2 — Strains used in this study. (PDF) [file pone.0018496.s007.pdf]

Supplemental Table S2. Strains used in this study.

K Number	A Number	Other ID	Genus	Species	Subspecies	Pathovar	Race, Biovar	Host	Location	Provider	ITS Amplicon (Present/ Absent)	ITS sequence (Obtained/ Not Obtained)	Nassar amplicon (Present/ Absent)	egl amplicon (Present/ Absent)	egl sequence (Obtained/Not Obtained/ Not Applicable)	RIF Amplicon (Present/ Absent)	RIF Sequence (Obtained/ Not Obtained)	RIF Pectobacterium amplification at 51°C (Present/ Absent)
K0001	A3292	GMI1000, QR85	*Ralstonia*	*solanacearum*			Race 1, Biovar 1	Tomato	French Guyana	C. Boucher	P	Obtained		P	Obtained	P	Obtained	
K0002	A3299		*Ralstonia*	*solanacearum*			Race 1, Biovar 1	Tomato	North Carolina, USA	L. Sequeira	P	NotObtained		A	N/A	P	Obtained	
K0003	A5370	BB	*Ralstonia*	*solanacearum*			Race 1, Biovar 1	Tomato	Hawaii, USA	B. Bushe	P	Obtained		P	Obtained	P	Obtained	
K0004	A5345	Rs-5	*Ralstonia*	*solanacearum*			Race 1, Biovar 1	Tomato	Florida, USA	M. T. Momol	P	Obtained		P	Obtained	P	Obtained	
K0005	A3450	UW30	*Ralstonia*	*solanacearum*			Race 1, Biovar 1	Tomato	Trinidad	D. Cook	P	Obtained		P	Obtained	P	Obtained	
K0006	A3381	167	*Ralstonia*	*solanacearum*			Race 2, Biovar 1	Banana	Costa Rica	D. Cook	P	Obtained		P	Obtained	P	Obtained	
K0007	A3912	909-128	*Ralstonia*	*solanacearum*			Race 2, Biovar 1	Banana	Hawaii, USA	S. Ferreira	P	Obtained		A	N/A	A	NotObtained	
K0008	A4125	BIOTE1694	*Ralstonia*	*solanacearum*			Race 2, Biovar 1	Banana	Philippines	M. Natural	P	Obtained		P	Obtained	P	Obtained	
K0009	A4522	PS6	*Ralstonia*	*solanacearum*			Race 2, F or D	Banana	Honduras	I.W. Buddenhagen	P	Obtained		P	Obtained	P	Obtained	
K0010	A4606	BD-1	*Ralstonia*	*solanacearum*			Blood Disease	Banana	Sulawesi, Indonesia	I.W. Buddenhagen	P	Obtained		P	Obtained	P	Obtained	
K0011	A4610	BD-4	*Ralstonia*	*solanacearum*			Blood Disease	Banana	Sulawesi, Indonesia	I.W. Buddenhagen	P	Obtained		P	Obtained	P	Obtained	
K0012	A4612	BD-6	*Ralstonia*	*solanacearum*			Blood Disease	Banana	Sulawesi, Indonesia	I.W. Buddenhagen	P	Obtained		P	NotObtained	P	Obtained	
K0013	A3903	P6	*Ralstonia*	*solanacearum*			Race 2, Biovar 1	Heliconia	Hawaii, USA	B. Bushe	P	Obtained		P	Obtained	P	Obtained	
K0014	A3906	KV1.1	*Ralstonia*	*solanacearum*			Race 2,	Heliconia	Hawaii, USA	S. Ferreira	P	Obtained		P	Obtained	P	NotObtained	

							Biovar 1											
K0015	A3908	KV2.2	*Ralstonia*	*solanacearum*			Race 2,<br>Biovar 1	Heliconia	Hawaii, USA	S. Ferreira	P	Obtained		P	Obtained	P	Obtained	
K0016	A5290	UW551	*Ralstonia*	*solanacearum*			Race 3,<br>Biovar 2	Geranium	Kenya	C. Allen	P	Obtained		P	Obtained	P	Obtained	
K0017	A4613	PS-11	*Ralstonia*	*solanacearum*			unknow<br>n Race	Geranium	Guatemala	D. Thomas	P	Obtained		P	Obtained	P	Obtained	
K0018	A3447	UW19	*Ralstonia*	*solanacearum*			Race 3<br>Biovar 2	Potato	Columbia	D. Cook	P	Obtained		P	Obtained	P	Obtained	
K0019	A5287	UW257	*Ralstonia*	*solanacearum*			Race 3,<br>Biovar 2	Potato	Costa Rica	C. Allen	P	Obtained		P	Obtained	P	Obtained	
K0020	A5289	UW550	*Ralstonia*	*solanacearum*			Race 3,<br>Biovar 2	Potato	Netherlands	C. Allen	P	Obtained		P	Obtained	P	Obtained	
K0021	A3786	ZO 36	*Ralstonia*	*solanacearum*			Race 1	Ginger	Philippines	M. Natural	P	Obtained		P	Obtained	P	Obtained	
K0022	A4515	GW-1	*Ralstonia*	*solanacearum*			Race 4	Ginger	Hawaii, USA	E. E. Trujillo	P	Obtained		P	Obtained	P	Obtained	
K0023	A4679		*Ralstonia*	*solanacearum*			Race 4	Ginger	Hawaii, USA	B. Bushe	P	NotObtained		A	N/A	P	Obtained	
K0024	A5192	PO-7	*Ralstonia*	*solanacearum*			Race 4	Ginger	Hawaii, USA	P. Hepperly	P	Obtained		P	NotObtained	P	Obtained	
K0025	A5719	D108	*Xanthomonas*	*axonopodis*		*dieffenbachia<br>e*		Anthurium	Hawaii, USA	A. Alvarez	P	NotObtained				P	Obtained	
K0026	A5720	D150	*Xanthomonas*	*axonopodis*		*dieffenbachia<br>e*		Anthurium	Hawaii, USA	A. Alvarez	P	Obtained				P	Obtained	
K0027	A5721	D182	*Xanthomonas*	*axonopodis*		*dieffenbachia<br>e*		Anthurium	Hawaii, USA	A. Alvarez	P	Obtained				P	Obtained	
K0028	A2650	J2	*Xanthomonas*	*axonopodis*		*dieffenbachia<br>e*		Anthurium	Jamaica	A. Alvarez	P	Obtained				P	Obtained	
K0029	A4957	A249	*Xanthomonas*	*campestris*		*campestris*		Cabbage	Hawaii, USA	A. Alvarez	P	NotObtained				P	Obtained	
K0030	A1135	EEXC114	*Xanthomonas*	*campestris*		*campestris*		Cabbage	North<br>Carolina,<br>USA	Echandi	P	Obtained				P	Obtained	
K0031	A4961	G2-12	*Xanthomonas*	*campestris*		*campestris*		Cabbage	Hawaii, USA	A. Alvarez	P	NotObtained				P	Obtained	
K0032	A4965	Xcc528,<br>ATCC 33913	*Xanthomonas*	*campestris*		*campestris*		Brassica<br>oleracea	United<br>Kingdom	ATCC	P	Obtained				P	Obtained	
K0033	A4954	G3-38A	*Xanthomonas*	*campestris*		*campestris*		Cabbage	Hawaii, USA	A. Alvarez	P	Obtained				P	Obtained	
K0034	A4964	X3	*Xanthomonas*	*campestris*		*campestris*		Cabbage	Florida,	J. Hunter	P	Obtained				P	Obtained	

									USA									
K0035	A1785	EWCII Com.gard	*Xanthomonas*	*euvesicatoria*				Pepper	Hawaii, USA	B. S. Kim	P	NotObtained				P	NotObtained	
K0036	A5342	M5	*Xanthomonas*	*citri*	*citri*			Citrus	Florida, USA	T. Schubert	P	NotObtained				P	Obtained	
K0037	A1942	83-4	*Xanthomonas*	*euvesicatoria*				Tomato	Florida, USA	J. B. Jones	P	NotObtained				P	NotObtained	
K0038	A3788	Xv138	*Xanthomonas*	*euvesicatoria*				Tomato	Indiana, USA	J. B. Jones	P	Obtained				P	Obtained	
K0039	A3013	Xc 62A	*Xanthomonas*	*citri*	*citri*			Citrus	Japan	E. Civerolo	P	Obtained				P	Obtained	
K0040	A3015	Xc 64B	*Xanthomonas*	*fuscans*	*aurantifolii*			Lemon	Argentina	E. Civerolo	P	Obtained				P	Obtained	
K0041	A3018	Xc 70C	*Xanthomonas*	*fuscans*	*aurantifolii*			Lime	Brazil	E. Civerolo	P	Obtained				P	Obtained	
K0042	A5337	2032	*Xanthomonas*	*citri*	*citri*			Citrus	Florida, USA	T. Schubert	P	Obtained				P	Obtained	
K0043	A5341	M9	*Xanthomonas*	*citri*	*citri*			Citrus	Florida, USA	T. Schubert	P	NotObtained				P	Obtained	
K0044	A2231	PXO 61	*Xanthomonas*	*oryzae*		*oryzae*	Race 1	Rice	Philippines	T. W. Mew	P	Obtained				P	Obtained	
K0045	A2595	PXO 158	*Xanthomonas*	*oryzae*		*oryzae*	Race 2	Rice	Philippines	P.Teng	P	NotObtained				P	Obtained	
K0046	A2495	PXO 69	*Xanthomonas*	*oryzae*		*oryzae*	Race 3	Rice	Philippines	T. W. Mew	P	Obtained				P	Obtained	
K0047	A2504	PXO 70	*Xanthomonas*	*oryzae*		*oryzae*	Race 4	Rice	Philippines	T. W. Mew	P	NotObtained				P	Obtained	
K0048	A2237	PXO 99	*Xanthomonas*	*oryzae*		*oryzae*	Race 5	Rice	Philippines	T. W. Mew	P	Obtained				P	Obtained	
K0073	A2058	H-160	*Clavibacter*	*michiganensis*	*michiganensis*			Tomato	Idaho, USA	H. Azad	P	Obtained				P	Obtained	
K0074	A4758	N 212	*Clavibacter*	*michiganensis*	*michiganensis*			Tomato	China	G. Hoyos	P	Obtained				P	Obtained	
K0075	A4763	N 7388A	*Clavibacter*	*michiganensis*	*michiganensis*			Tomato	Morocco	G. Hoyos	P	Obtained				P	Obtained	
K0076	A4757	N 211	*Clavibacter*	*michiganensis*	*michiganensis*			Tomato	China	G. Hoyos	P	Obtained				P	Obtained	
K0077	A4691	cmm 462	*Clavibacter*	*michiganensis*	*michiganensis*			Tomato	Portugal	M. S. Santos	P	Obtained				P	Obtained	
K0078	A1749	A 518-5	*Clavibacter*	*michiganensis*	*michiganensis*			Tomato	Hawaii, USA	W.Chun	P	Obtained				P	Obtained	
K0079	A4755	N 202A	*Clavibacter*	*michiganensis*	*michiganensi*			Tomato	Chile	G. Hoyos	P	Obtained				P	Obtained	

					s													
K0080	A4918	E3	*Clavibacter*	*michiganensis*	*michiganensis*			Pepper	Ohio, USA	M. Ivey	P	Obtained				P	Obtained	
K0081	A5747	C222	*Clavibacter*	*michiganensis*	*michiganensis*			Tomato	Oregon, USA	L. di Nitto	P	Obtained				P	Obtained	
K0082	A4791	71421	*Clavibacter*	*michiganensis*	*michiganensis*			Tomato	China	D. Coutu	P	Obtained				P	Obtained	
K0083	A1753	A 438-1	*Clavibacter*	*michiganensis*	*michiganensis*			Tomato	Hawaii, USA	W.Chun	P	Obtained				P	Obtained	
K0084	A4748	IPO 543	*Clavibacter*	*michiganensis*	*michiganensis*			Tomato	Kenya	I. de Vries	P	NotObtained				P	Obtained	
K0085	A2645	S47	*Clavibacter*	*michiganensis*	*michiganensis*			Tomato	California, USA	H. Bolkan	P	Obtained				P	Obtained	
K0086	A4588	cmm016	*Clavibacter*	*michiganensis*	*michiganensis*			Tomato	Washington, USA	M. Derie	P	NotObtained				P	Obtained	
K0087	A2974	OSURSC76	*Clavibacter*	*michiganensis*	*michiganensis*			Tomato	Ohio, USA	R. Clevenstine	P	Obtained				P	Obtained	
K0088	A5131	ZUM 3036	*Clavibacter*	*michiganensis*	*michiganensis*			Tomato	Netherlands	A. Alvarez	P	Obtained				P	Obtained	
K0089	A5748	C217	*Clavibacter*	*michiganensis*	*michiganensis*			Tomato	Oregon, USA	L. di Nitto	P	Obtained				P	Obtained	
K0090	A1146	QR-77 (ATCC9850)	*Clavibacter*	*michiganensis*	*sepedonicus*			Potato	USA	W. H. Burkholder	P	Obtained				P	Obtained	
K0091	A1149	QR-80 (ATCC10253)	*Clavibacter*	*michiganensis*	*insidiosus*			Alfalfa	Kansas, USA	W. H. Burkholder	P	Obtained				P	Obtained	
K0093	A4750	IPO 545	*Clavibacter*	*michiganensis*	*michiganensis*			Tomato	South Africa	I. de Vries	P	NotObtained				P	Obtained	
K0094	A4780	71169	*Clavibacter*	*michiganensis*	*michiganensis*			Tomato	China	D. Coutu	P	Obtained				P	Obtained	
K0095	A5749	C91-A	*Clavibacter*	*michiganensis*	*michiganensis*			Tomato	California, USA	W. Kaneshiro	P	NotObtained				P	Obtained	
K0096	A5750	C6-A	*Clavibacter*	*michiganensis*	*michiganensis*			Tomato	California, USA	W. Kaneshiro	P	NotObtained				P	Obtained	
K0097	A3290	A476-1A	*Ralstonia*	*solanacearum*			Race 1	Pepper	Hawaii, USA	A. Alvarez	P	Obtained		P	NotObtained	P	Obtained	

K0098	A3291	A616	*Ralstonia*	*solanacearum*			Race 1	Tomato	Hawaii, USA	A. Alvarez	P	Obtained		P	NotObtained	P	Obtained	
K0099	A3293	QR-88, K60	*Ralstonia*	*solanacearum*			Race 1	Tomato	North Carolina, USA	A. Kelman	P	Obtained		P	NotObtained	P	Obtained	
K0100	A3294	QR-87, B-1	*Ralstonia*	*solanacearum*			Race 1	Tomato	North Carolina, USA	A. Kelman	P	Obtained		P	NotObtained	P	Obtained	
K0101	A3313	001	*Ralstonia*	*solanacearum*			Race 1	Peanut	Indonesia	A. C. Hayward	P	Obtained		P	NotObtained	P	Obtained	
K0102	A3314	732	*Ralstonia*	*solanacearum*			Race 1	Peanut	Indonesia	A. C. Hayward	P	Obtained		P	Obtained	P	Obtained	
K0103	A3315	171	*Ralstonia*	*solanacearum*			Race 1	Peanut	Indonesia	A. C. Hayward	P	NotObtained		A	N/A	P	NotObtained	
K0104	A3316	1017	*Ralstonia*	*solanacearum*			Race 1	Peanut	Indonesia	A. C. Hayward	P	Obtained		P	Obtained	P	Obtained	
K0105	A3317	3699	*Ralstonia*	*solanacearum*			Race 1	Peanut	Indonesia	A. C. Hayward	P	Obtained		P	Obtained	P	Obtained	
K0106	A3318	190	*Ralstonia*	*solanacearum*			Race 1	Peanut	Indonesia	A. C. Hayward	P	Obtained		P	Obtained	P	Obtained	
K0107	A3319	234	*Ralstonia*	*solanacearum*			Race 1	Peanut	Indonesia	A. C. Hayward	P	Obtained		P	Obtained	P	Obtained	
K0108	A3344	A476-1B	*Ralstonia*	*solanacearum*			Race 1	Bell Pepper	Hawaii, USA	A. Alvarez	P	Obtained		P	NotObtained	P	Obtained	
K0109	A3445	UW23	*Ralstonia*	*solanacearum*			Race 3, Biovar 2	Potato	Israel	D. Cook (Volcani)	P	Obtained		A	N/A	A	NotObtained	
K0110	A3446	UW255	*Ralstonia*	*solanacearum*			Race 1, Biovar 3	Potato	Costa Rica	D. Cook (Gonzalez)	P	NotObtained		P	NotObtained	P	Obtained	
K0111	A3448	UW378	*Ralstonia*	*solanacearum*			Race 1, Biovar 4	Olive	China	D. Cook (He)	P	Obtained		P	NotObtained	P	Obtained	
K0112	A3449	UW278	*Ralstonia*	*solanacearum*			Race 1, Biovar 1	Tobacco	Mexico	D. Cook (Fucikovsky)	P	Obtained		P	Obtained	P	Obtained	
K0113	A3451	UW151	*Ralstonia*	*solanacearum*			Race 1, Biovar 4	Ginger	Australia	D. Cook (Hayward)	P	Obtained		P	Obtained	P	Obtained	
K0114	A3452	UW359	*Ralstonia*	*solanacearum*			Race 1, Biovar 4	Ginger	China	D. Cook (He)	P	NotObtained		P	Obtained	P	Obtained	
K0115	A3453	UW256	*Ralstonia*	*solanacearum*			Race 1, Biovar 1	Potato	Costa Rica	D. Cook (Gonzalez)	P	Obtained		A	N/A	P	Obtained	
K0116	A3454	UW147	*Ralstonia*	*solanacearum*			Race 1, Biovar 3	Tobacco	Australia	D. Cook(Hayward)	P	NotObtained		A	N/A	P	Obtained	
K0117	A3455	UW73	*Ralstonia*	*solanacearum*			Race 3, Biovar 2	Potato	Sri Lanka	D. Cook	P	Obtained		P	NotObtained	P	NotObtained	
K0118	A3456	UW152	*Ralstonia*	*solanacearum*			Race 1,	Potato	Australia	D. Cook	P	NotObtained		P	Obtained	P	Obtained	

							Biovar 3			(Hayward)								
K0119	A3457	UW27	*Ralstonia*	*solanacearum*			Race 1,<br>Biovar 4	Tobacco	Florida,<br>USA	D. Cook<br>(Kelman)	P	Obtained		P	Obtained	P	Obtained	
K0120	A3458	UW154	*Ralstonia*	*solanacearum*			Race 1,<br>Biovar 1	Tobacco	Colombia	D. Cook<br>(Grenada)	P	Obtained		A	N/A	P	Obtained	
K0121	A3459	UW130	*Ralstonia*	*solanacearum*			Race 1,<br>Biovar 3	Tomato	Peru	D. Cook<br>(Sequeira)	A	Obtained		P	NotObtained	P	Obtained	
K0122	A3460	UW119	*Ralstonia*	*solanacearum*			Race 1,<br>Biovar 3	Potato	Costa Rica	D. Cook<br>(Gonzalez)	P	Obtained		P	NotObtained	P	Obtained	
K0123	A3461	UW8	*Ralstonia*	*solanacearum*			Race 1,<br>Biovar 3	Eupatorium<br>odoratum	Costa Rica	D. Cook<br>(Sequeira)	P	Obtained		P	Obtained	P	Obtained	
K0124	A3462	UW373	*Ralstonia*	*solanacearum*			Race 1,<br>Biovar 5	Mulberry	China	D. Cook (He)	P	Obtained		P	NotObtained	P	Obtained	
K0125	A3463	UW275	*Ralstonia*	*solanacearum*			Race 1,<br>Biovar 1	Melanpodium<br>perfoliatum	Costa Rica	D. Cook<br>(Sequeira)	P	Obtained		P	Obtained	P	Obtained	
K0126	A3464	UW360	*Ralstonia*	*solanacearum*			Race 1,<br>Biovar 4	Mulberry	China	D. Cook (He)	P	Obtained		P	Obtained	P	Obtained	
K0127	A3465	UW26	*Ralstonia*	*solanacearum*			Race 1,<br>Biovar 1	Tomato	Georgia,<br>USA	D. Cook<br>(Kelman)	P	Obtained		P	Obtained	P	Obtained	
K0128	A3466	UW141	*Ralstonia*	*solanacearum*			Race 1,<br>Biovar 4	Ginger	Australia	D. Cook<br>(Hayward)	P	Obtained		P	Obtained	P	Obtained	
K0129	A3467	UW380	*Ralstonia*	*solanacearum*			Race 1,<br>Biovar 3	Olive	China	D. Cook (He)	P	NotObtained		A	N/A	A	NotObtained	
K0130	A3468	UW90	*Ralstonia*	*solanacearum*			Race 1,<br>Biovar 1	Tobacco	Brazil	D. Cook<br>(Robbs)	P	NotObtained		P	Obtained	P	Obtained	
K0131	A3469	UW74	*Ralstonia*	*solanacearum*			Race 1,<br>Biovar 4	Potato	Sri Lanka	D. Cook	P	Obtained		A	N/A	P	Obtained	
K0132	A3470	UW361	*Ralstonia*	*solanacearum*			Race 1,<br>Biovar 5	Mulberry	China	D. Cook (He)	P	NotObtained		P	Obtained	P	Obtained	
K0133	A3527	1605WS	*Ralstonia*	*solanacearum*			Race 1	Peanut	Indonesia	Y. Maryudani	P	Obtained		P	NotObtained	P	Obtained	
K0134	A3528	1405WS	*Ralstonia*	*solanacearum*			Race 1	Peanut	Indonesia	Y. Maryudani	P	Obtained		P	Obtained	P	Obtained	
K0135	A3529	1005Mr	*Ralstonia*	*solanacearum*			Race 1	Peanut	Indonesia	Y. Maryudani	P	Obtained		P	Obtained	P	Obtained	
K0136	A3530	ENO290	*Ralstonia*	*solanacearum*			Race 1	Peanut	Indonesia	Y. Maryudani	P	Obtained		P	Obtained	P	Obtained	
K0137	A3531	T435	*Ralstonia*	*solanacearum*			Race 1	Peanut	Indonesia	Y. Maryudani	P	Obtained		P	Obtained	P	Obtained	

K0138	A3532	T440	*Ralstonia*	*solanacearum*			Race 1	Peanut	Indonesia	Y. Maryudani	P	Obtained		P	Obtained	P	Obtained	
K0139	A3533	2305L	*Ralstonia*	*solanacearum*			Race 1	Peanut	Indonesia	Y. Maryudani	P	NotObtained		P	Obtained	P	Obtained	
K0140	A3534	S718	*Ralstonia*	*solanacearum*			Race 1	Peanut	Indonesia	Y. Maryudani	P	NotObtained		P	Obtained	P	Obtained	
K0141	A3535	1905Mr	*Ralstonia*	*solanacearum*			Race 1	Peanut	Indonesia	Y. Maryudani	P	NotObtained		P	Obtained	P	Obtained	
K0142	A3536	1105B	*Ralstonia*	*solanacearum*			Race 1	Peanut	Indonesia	Y. Maryudani	P	Obtained		P	Obtained	P	Obtained	
K0143	A3587	Biovar 4-90.5	*Ralstonia*	*solanacearum*			Race 1	Sweet Potato	China	L. Y. He	P	Obtained		P	NotObtained	P	Obtained	
K0144	A3772	A12	*Ralstonia*	*solanacearum*			Race 1	Ampalaya	Philippines	M. Natural	P	Obtained		P	Obtained	P	Obtained	
K0145	A3776	T6	*Ralstonia*	*solanacearum*			Race 1	Tomato	Philippines	M. Natural	P	Obtained		P	NotObtained	P	Obtained	
K0146	A3777	C2	*Ralstonia*	*solanacearum*			Race 1	Peanut	Philippines	M. Natural	P	Obtained		P	Obtained	P	NotObtained	
K0147	A4492	S-118, K119, UW6	*Ralstonia*	*solanacearum*			Race 2	Heliconia	Costa Rica	L. Sequeira	P	Obtained		P	Obtained	P	Obtained	
K0148	A3779	WP17	*Ralstonia*	*solanacearum*			Race 1	Potato	Philippines	M. Natural	P	Obtained		P	Obtained	P	Obtained	
K0149	A3780	SQ21	*Ralstonia*	*solanacearum*			Race 1	Squash	Philippines	M. Natural	P	Obtained		P	Obtained	P	Obtained	
K0150	A3784	MT1	*Ralstonia*	*solanacearum*			Race 2	Abaca	Philippines	M. Natural	P	Obtained		P	Obtained	P	Obtained	
K0151	A3904	P5	*Ralstonia*	*solanacearum*			Race 2	Heliconia	Hawaii, USA	B. Bushe	P	NotObtained		P	Obtained	P	Obtained	
K0152	A3907	KV1.2	*Ralstonia*	*solanacearum*			Race 2	Heliconia	Hawaii, USA	S. Ferreira	P	Obtained		P	Obtained	P	Obtained	
K0153	A3909	KV3.1	*Ralstonia*	*solanacearum*			Race 2	Heliconia	Hawaii, USA	S. Ferreira	P	NotObtained		P	Obtained	P	Obtained	
K0154	A3910	909-87	*Ralstonia*	*solanacearum*			Race 2	Heliconia	Hawaii, USA	S. Ferreira	P	NotObtained		P	Obtained	P	Obtained	
K0155	A3911	C1017-1A	*Ralstonia*	*solanacearum*			Race 2	Heliconia	Hawaii, USA	S. Ferreira	P	Obtained		W	Obtained	P	Obtained	
K0156	A3913	KV5.1	*Ralstonia*	*solanacearum*			Race 2	Heliconia	Hawaii, USA	S. Ferreira	P	Obtained		P	Obtained	P	Obtained	
K0157	A4071	317	*Ralstonia*	*solanacearum*			Race 2	Heliconia	Hawaii, USA	B. Bushe	P	Obtained		A	N/A	P	Obtained	
K0158	A4087	399CP	*Ralstonia*	*solanacearum*			Race 4	Ginger	Hawaii, USA	B. Bushe	P	Obtained		P	Obtained	P	NotObtained	
K0159	A4492	S-118, K119, UW6	*Ralstonia*	*solanacearum*			Race 2	Heliconia	Costa Rica	L. Sequeira	P	Obtained		P	Obtained	P	Obtained	
K0160	A4126	BIOTE1705	*Ralstonia*	*solanacearum*			Race 2	Banana	Philippines	M. Natural	P	Obtained		P	Obtained	P	Obtained	
K0161	A4127	BIOTE1713	*Ralstonia*	*solanacearum*			Race 2	Banana	Philippines	M. Natural	P	Obtained		P	Obtained	P	Obtained	
K0162	A4128	BIOTE1690	*Ralstonia*	*solanacearum*			Race 2	Banana	Philippines	M. Natural	P	Obtained		P	Obtained	P	Obtained	
K0163	A4129	BIOTE1709	*Ralstonia*	*solanacearum*			Race 2	Banana	Philippines	M. Natural	P	Obtained		A	N/A	P	Obtained	
K0164	A4130	BIOTE1702	*Ralstonia*	*solanacearum*			Race 2	Banana	Philippines	M. Natural	P	Obtained		P	Obtained	P	Obtained	
K0165	A4131	BIOTE1699	*Ralstonia*	*solanacearum*			Race 2	Banana	Philippines	M. Natural	P	Obtained		P	Obtained	P	Obtained	

K0166	A4132	BIOTE1715	*Ralstonia*	*solanacearum*			Race 2	Banana	Philippines	M. Natural	P	Obtained		P	NotObtained	P	Obtained	
K0167	A4133	BIOTE1707	*Ralstonia*	*solanacearum*			Race 2	Banana	Philippines	M. Natural	P	Obtained		A	N/A	P	Obtained	
K0168	A4380	38606	*Ralstonia*	*solanacearum*			Race 2	Banana	India	S. S. Gnanamanicka m	P	Obtained		P	Obtained	P	Obtained	
K0169	A4637	6C-1	*Ralstonia*	*solanacearum*			Blood Disease	Banana	Indonesia	J. Bartlett	P	Obtained		P	Obtained	P	Obtained	
K0170	A4493	S-164, K204, UW 10	*Ralstonia*	*solanacearum*			Race 2	Heliconia	Costa Rica	L. Sequeira	P	Obtained		P	Obtained	P	Obtained	
K0171	A4494	S-200, UW 15	*Ralstonia*	*solanacearum*			Race 2, SFR	Banana (Cavendish)	Honduras	L. Sequeira	P	Obtained		P	Obtained	P	Obtained	
K0172	A4495	S215, K260, UW20	*Ralstonia*	*solanacearum*			Race 2	Banana	Venezuela	I.W. Buddenhagen	P	Obtained		P	Obtained	P	Obtained	
K0173	A4642	Sample3	*Ralstonia*	*solanacearum*			Blood Disease	Banana	Indonesia	J. Bartlett	P	Obtained		P	Obtained	P	Obtained	
K0174	A4516	GW-2	*Ralstonia*	*solanacearum*			Race 4	Ginger	Hawaii, USA	E. E. Trujillo	P	Obtained		P	Obtained	P	Obtained	
K0175	A4519	PS3	*Ralstonia*	*solanacearum*			Race 2	Plantain	Honduras	I.W. Buddenhagen	P	Obtained		P	Obtained	P	Obtained	
K0176	A4520	PS4	*Ralstonia*	*solanacearum*			Race 2	Banana	Philippines	I.W. Buddenhagen	P	Obtained		P	Obtained	P	Obtained	
K0177	A4523	PS7	*Ralstonia*	*solanacearum*			Race 2	Banana	Honduras	I.W. Buddenhagen	P	Obtained		P	Obtained	P	Obtained	
K0178	A4524	PS8/BSF I	*Ralstonia*	*solanacearum*			Race 2	Banana	Honduras	I.W. Buddenhagen	P	Obtained		P	Obtained	P	Obtained	
K0179	A4526	PS10/BS I	*Ralstonia*	*solanacearum*			Race 2	Banana	Honduras	I.W. Buddenhagen	P	NotObtained		P	Obtained	P	NotObtained	
K0180	A4527	PS11/M I	*Ralstonia*	*solanacearum*			Race 2	Banana	Honduras	I.W. Buddenhagen	P	NotObtained		P	Obtained	P	NotObtained	
K0181	A4528	PS12/M 2	*Ralstonia*	*solanacearum*			Race 2	Banana	Honduras	I.W. Buddenhagen	P	Obtained		P	Obtained	P	Obtained	
K0182	A4546	PSCA3	*Ralstonia*	*solanacearum*			Race 2	Banana	Honduras	I.W. Buddenhagen	P	Obtained		A	N/A	P	Obtained	
K0183	A4547	PSCA4	*Ralstonia*	*solanacearum*			Race 2	Banana	Honduras	I.W. Buddenhagen	P	NotObtained		P	NotObtained	P	Obtained	
K0184	A4548	PSCA5	*Ralstonia*	*solanacearum*			Race 2	Banana	Honduras	I.W.	P	Obtained		P	NotObtained	P	Obtained	

										Buddenhagen								
K0185	A4549	PSCA6	*Ralstonia*	*solanacearum*			Race 2	Banana	Honduras	I.W. Buddenhagen	P	Obtained		P	Obtained	P	Obtained	
K0186	A4550	PSCA7	*Ralstonia*	*solanacearum*			Race 2	Banana	Honduras	I.W. Buddenhagen	P	Obtained		P	Obtained	P	Obtained	
K0187	A4607	BD-1 mutant	*Ralstonia*	*solanacearum*			Blood Disease	Banana	Sulawesi, Indonesia	I.W. Buddenhagen	P	Obtained		P	Obtained	P	Obtained	
K0188	A4608	BD-2	*Ralstonia*	*solanacearum*			Blood Disease	Banana	Sulawesi, Indonesia	I.W. Buddenhagen	P	Obtained		P	Obtained	P	Obtained	
K0189	A4609	BD-3	*Ralstonia*	*solanacearum*			Blood Disease	Banana	Sulawesi, Indonesia	I.W. Buddenhagen	P	Obtained		P	Obtained	P	Obtained	
K0190	A4611	BD-5	*Ralstonia*	*solanacearum*			Blood Disease	Banana	Sulawesi, Indonesia	I.W. Buddenhagen	P	Obtained		P	Obtained	P	Obtained	
K0191	A4635	6A-1	*Ralstonia*	*solanacearum*			Blood Disease	Banana	Indonesia	J. Bartlett	P	Obtained		P	Obtained	P	Obtained	
K0192	A4636	6B-1	*Ralstonia*	*solanacearum*			Blood Disease	Banana	Indonesia	J. Bartlett	P	Obtained		P	NotObtained	P	Obtained	
K0193	A4929	XLS-2	*Xanthomonas*	*campestris*		*armoraciae*		Cabbage	Louisiana, USA	L. Black	P	Obtained				P	Obtained	
K0194	A4930	XLS-3	*Xanthomonas*	*campestris*		*armoraciae*		Cabbage	Louisiana, USA	L. Black	P	Obtained				P	Obtained	
K0195	A4931	XLS-5	*Xanthomonas*	*campestris*		*armoraciae*		Cabbage	Louisiana, USA	L. Black	P	Obtained				P	Obtained	
K0196	A4932	XLS-10	*Xanthomonas*	*campestris*		*armoraciae*		Cabbage	California, USA	J. Watterson	P	Obtained				P	Obtained	
K0197	A4933	XLS-11	*Xanthomonas*	*campestris*		*armoraciae*		Cabbage	Louisiana, USA	L. Black	P	Obtained				P	Obtained	
K0198	A4934	XLS-13	*Xanthomonas*	*campestris*		*armoraciae*		Cabbage	Minnesota, USA	L. Black	P	Obtained				P	Obtained	
K0199	A4935	XLS-22	*Xanthomonas*	*campestris*		*armoraciae*		Cabbage	Florida, USA	L. Black	P	Obtained				P	Obtained	
K0200	A4944	XLS-1	*Xanthomonas*	*campestris*		*armoraciae*		Cabbage	Louisiana, USA	L. Black	P	Obtained				P	Obtained	
K0201	A4936	XLS-24	*Xanthomonas*	*campestris*		*armoraciae*		Cabbage	Louisiana, USA	L. Black	P	NotObtained				P	Obtained	

K0202	A4937	XLS-25	*Xanthomonas*	*campestris*		*armoraciae*		Cabbage	Louisiana, USA	L. Black	P	Obtained				P	Obtained	
K0203	A4938	XLS-26	*Xanthomonas*	*campestris*		*armoraciae*		Cabbage	Louisiana, USA	L. Black	P	Obtained				P	Obtained	
K0204	A4939	XCR-1, NCPPB 1946	*Xanthomonas*	*campestris*		*raphani*		Radish	New Zealand	L. Black	P	Obtained				P	Obtained	
K0205	A4940	XCR-3, PDDCC 1640	*Xanthomonas*	*campestris*		*raphani*		Radish	United Kingdom	L. Black	P	NotObtained				P	Obtained	
K0206	A4941	XCR-4, PDDCC 1641	*Xanthomonas*	*campestris*		*raphani*		Radish	United Kingdom	L. Black	P	Obtained				P	NotObtained	
K0207	A4942	XCR-5, PDDCC 1645	*Xanthomonas*	*campestris*		*raphani*		Radish	United Kingdom	L. Black	P	Obtained				P	Obtained	
K0208	A4943	XCA-3	*Xanthomonas*	*campestris*		*armoraciae*		Candytuff	Tanzania	L. Black	P	Obtained				P	Obtained	
K0209	A4945	XCA-2	*Xanthomonas*	*campestris*		*armoraciae*		Candytuff	Tanzania	L. Black	P	Obtained				P	Obtained	
K0210	A4946	756	*Xanthomonas*	*campestris*		*armoraciae*		Cabbage	California, USA	J. Watterson	P	Obtained				P	Obtained	
K0211	A4947	A342	*Xanthomonas*	*campestris*		*armoraciae*		Broccoli	Hawaii, USA	A. Alvarez	P	Obtained				P	Obtained	
K0212	A4948	G3-27	*Xanthomonas*	*campestris*		*armoraciae*		Cabbage seed	Hawaii, USA	A. Alvarez	P	Obtained				P	Obtained	
K0213	A4949	XLS-10	*Xanthomonas*	*campestris*		*armoraciae*		Cabbage seed	Louisiana, USA	L. Black	P	Obtained				P	Obtained	
K0214	A4950	417	*Xanthomonas*	*campestris*		*armoraciae*		Cabbage	California, USA	J. Watterson	P	Obtained				P	Obtained	
K0215	A4951	XLS-2	*Xanthomonas*	*campestris*		*armoraciae*		Cabbage	Louisiana, USA	L. Black	P	Obtained				P	Obtained	
K0216	A4952	XLS-6	*Xanthomonas*	*campestris*		*armoraciae*		Cabbage	Louisiana, USA	L. Black	P	NotObtained				P	Obtained	
K0217	A4953	GAC-20	*Xanthomonas*	*campestris*		*campestris*		Cabbage	Hawaii, USA	A. Alvarez	P	Obtained				P	Obtained	
K0218	A4954	G3-38A	*Xanthomonas*	*campestris*		*campestris*		Cabbage	Hawaii, USA	A. Alvarez	P	Obtained				P	Obtained	
K0219	A4955	GAC-137	*Xanthomonas*	*campestris*		*campestris*		Cabbage	Hawaii, USA	A. Alvarez	P	Obtained				P	Obtained	
K0220	A4956	GAC-17	*Xanthomonas*	*campestris*		*campestris*		Cabbage	Hawaii, USA	A. Alvarez	P	NotObtained				P	Obtained	
K0221	A4957	A249	*Xanthomonas*	*campestris*		*campestris*		Cabbage	Hawaii, USA	A. Alvarez	P	Obtained				P	Obtained	

K0222	A4958	PHW46	*Xanthomonas*	*campestris*		*campestris*		Brassica oleracea	Wisconsin, USA	P. Williams	P	NotObtained				P	Obtained	
K0223	A4959	HM	*Xanthomonas*	*campestris*		*campestris*		Cabbage	New York, USA	J. Hunter	P	Obtained				P	Obtained	
K0224	A4960	EEXC114	*Xanthomonas*	*campestris*		*campestris*		Cabbage	North Carolina, USA	E. Echandi	P	NotObtained				P	Obtained	
K0225	A4964	X3	*Xanthomonas*	*campestris*		*campestris*		Cabbage	Florida, USA	J. Hunter	P	Obtained				P	Obtained	
K0226	A4961	G2-12	*Xanthomonas*	*campestris*		*campestris*		Cabbage	Hawaii, USA	A. Alvarez	P	Obtained				P	Obtained	
K0227	A4962	G2-17	*Xanthomonas*	*campestris*		*campestris*		Cabbage	Hawaii, USA	A. Alvarez	P	Obtained				P	Obtained	
K0228	A4963	A3263	*Xanthomonas*	*campestris*		*campestris*		Brussel sprouts	United Kingdom	R. Gilaitis	P	Obtained				P	Obtained	
K0229	A1299	DR-63a purified	*Xanthomonas*	*maltophilia*				Cabbage seed	California, USA	A. Alvarez	P	Obtained				P	Obtained	
K0230	A1308	DR-79 purified	*Xanthomonas*	*maltophilia*				Cabbage seed	California, USA	A. Alvarez	P	Obtained				P	Obtained	
K0231	A1823	GAC 87aR	*Xanthomonas*	*campestris*		*campestris*		Cabbage	Hawaii, USA	A. Alvarez	P	Obtained				P	NotObtained	
K0232	A1826	GAC 119	*Xanthomonas*	*campestris*		*campestris*		Cabbage	Hawaii, USA	A. Alvarez	P	NotObtained				P	Obtained	
K0233	A1902	KOGF 8D	*Xanthomonas*	*campestris*		*campestris*		Cabbage	Hawaii, USA	A. Alvarez	P	NotObtained				P	Obtained	
K0234	A1908	KOGF 7A	*Xanthomonas*	*campestris*		*campestris*		Cabbage	Hawaii, USA	A. Alvarez	P	Obtained				P	Obtained	
K0235	A1909	KOGF 7B	*Xanthomonas*	*campestris*		*campestris*		Cabbage	Hawaii, USA	A. Alvarez	P	Obtained				P	Obtained	
K0236	A1910	A206.2A	*Xanthomonas*	*campestris*		*campestris*		Cabbage	Hawaii, USA	A. Alvarez	P	Obtained				P	Obtained	
K0237	A2125	XCC-Caberet	*Xanthomonas*	*campestris*		*campestris*		Cabbage	New York, USA	J. Hunter	P	Obtained				P	Obtained	
K0238	A2347	1A1	*Xanthomonas*	*campestris*		*campestris*		Cabbage	Panama	R. Alvarez	P	Obtained				P	Obtained	
K0239	A2352	1C2	*Xanthomonas*	*campestris*		*campestris*		Cabbage	Panama	R. Alvarez	P	NotObtained				P	Obtained	
K0240	A1915	KOGF 7EL	*Xanthomonas*	*campestris*		*campestris*		Cabbage	Hawaii, USA	A. Alvarez	P	Obtained				P	Obtained	
K0241	A2351	1C1	*Xanthomonas*	*campestris*		*campestris*		Cabbage	Panama	R. Alvarez	P	Obtained				P	Obtained	
K0242	A2521	Br2	*Xanthomonas*	*campestris*		*campestris*		Cabbage	Panama	R. Alvarez	P	Obtained				P	Obtained	
K0243	A2110	D147	*Xanthomonas*	*axonopodis*		*dieffenbachia e*		Anthurium	Hawaii, USA	R. Lipp	P	Obtained				P	Obtained	
K0244	A2111	D194	*Xanthomonas*	*axonopodis*		*dieffenbachia*		Colocasia	Hawaii, USA	R. Lipp	P	Obtained				P	Obtained	

						e												
K0245	A3052	K3-1-12	*Xanthomonas*	*campestris*		*campestris*		Cabbage	Hawaii, USA	K.W. Liew	P	Obtained				P	Obtained	
K0246	A3053	RR68	*Xanthomonas*	*campestris*		*campestris*		Cabbage	Wisconsin, USA	P. Williams	P	Obtained				P	Obtained	
K0247	A3054	EEXC114	*Xanthomonas*	*campestris*		*campestris*		Cabbage	North Carolina, USA	E. Echandi	P	Obtained				P	Obtained	
K0248	A4834	DhC-1	*Xanthomonas*	*campestris*		*campestris*		Cabbage	Dhusalar, Nepal	D. Shakya	P	NotObtained				P	Obtained	
K0249	A4835	DhC-2	*Xanthomonas*	*campestris*		*campestris*		Cabbage	Dhusalar, Nepal	D. Shakya	P	NotObtained				P	Obtained	
K0250	A4836	DhC-3	*Xanthomonas*	*campestris*		*campestris*		Cabbage	Dhusalar, Nepal	D. Shakya	P	NotObtained				P	Obtained	
K0251	A4837	DhC-4	*Xanthomonas*	*campestris*		*campestris*		Cabbage	Dhusalar, Nepal	D. Shakya	P	NotObtained				P	Obtained	
K0252	A4838	ChaC-1	*Xanthomonas*	*campestris*		*campestris*		Cabbage	Charaudi, Nepal	D. Shakya	P	NotObtained				P	Obtained	
K0253	A4839	ChaC-2	*Xanthomonas*	*campestris*		*campestris*		Cabbage	Charaudi, Nepal	D. Shakya	P	Obtained				P	Obtained	
K0254	A4840	ChiFC-1	*Xanthomonas*	*campestris*		*campestris*		Cabbage	Chitwan Fasera, Nepal	D. Shakya	P	NotObtained				P	Obtained	
K0255	A4841	ChiFC-2	*Xanthomonas*	*campestris*		*campestris*		Cabbage	Chitwan Fasera, Nepal	D. Shakya	P	NotObtained				P	Obtained	
K0256	A4842	ChiKB-1	*Xanthomonas*	*campestris*		*campestris*		Broccoli	Chitwan Khairani, Nepal	D. Shakya	P	NotObtained				P	Obtained	
K0257	A4843	ChiKB-2	*Xanthomonas*	*campestris*		*campestris*		Broccoli	Chitwan Khairani, Nepal	D. Shakya	P	Obtained				P	Obtained	
K0258	A5722	D-3-L	*Xanthomonas*	*axonopodis*		*dieffenbachiae*		Aglonema	Hawaii, USA	R. Lipp	P	Obtained				P	NotObtained	
K0259	A5723	D-15-3	*Xanthomonas*	*axonopodis*		*dieffenbachiae*		Anthurium	Hawaii, USA	R. Lipp	P	NotObtained				P	Obtained	

K0260	A5724	D-16-2	*Xanthomonas*	*axonopodis*		*dieffenbachiae*		Anthurium	Hawaii, USA	R. Lipp	P	Obtained				P	Obtained	
K0261	A5725	D-21-2	*Xanthomonas*	*axonopodis*		*dieffenbachiae*		Anthurium	Hawaii, USA	R. Lipp	P	Obtained				P	Obtained	
K0262	A5726	D-27-2-L	*Xanthomonas*	*axonopodis*		*dieffenbachiae*		Aglonema	Hawaii, USA	R. Lipp	P	Obtained				P	Obtained	
K0263	A5727	D-34-3a	*Xanthomonas*	*axonopodis*		*dieffenbachiae*		Anthurium	Hawaii, USA	R. Lipp	P	Obtained				P	Obtained	
K0264	A5728	D-34-3b	*Xanthomonas*	*axonopodis*		*dieffenbachiae*		Anthurium	Hawaii, USA	R. Lipp	P	Obtained				P	Obtained	
K0265	A5717	D-36-1	*Xanthomonas*	*axonopodis*		*dieffenbachiae*		Syngonium	Hawaii, USA	R. Lipp	P	Obtained				P	Obtained	
K0266	A5729	D-37	*Xanthomonas*	*axonopodis*		*dieffenbachiae*		Epipremnum	Hawaii, USA	R. Lipp	P	Obtained				P	Obtained	
K0267	A5730	D-38-3	*Xanthomonas*	*axonopodis*		*dieffenbachiae*		Anthurium	Hawaii, USA	R. Lipp	P	NotObtained				P	Obtained	
K0268	A5731	D-56-3	*Xanthomonas*	*axonopodis*		*dieffenbachiae*		Anthurium	Hawaii, USA	R. Lipp	P	Obtained				P	Obtained	
K0269	A5732	D-14-2	*Xanthomonas*	*axonopodis*		*dieffenbachiae*		Anthurium	Hawaii, USA	R. Lipp	P	Obtained				P	Obtained	
K0270	A4723	D71.1	*Xanthomonas*	*axonopodis*		*dieffenbachiae*		Anthurium	Hawaii, USA	T. Sakai	P	Obtained				P	Obtained	
K0271	A5733	D-88-1	*Xanthomonas*	*axonopodis*		*dieffenbachiae*		Anthurium	Hawaii, USA	R. Lipp	P	Obtained				P	Obtained	
K0272	A5734	D-91a	*Xanthomonas*	*axonopodis*		*dieffenbachiae*		Anthurium	Hawaii, USA	R. Lipp	P	Obtained				P	Obtained	
K0273	A5735	D-92	*Xanthomonas*	*axonopodis*		*dieffenbachiae*		Anthurium	Hawaii, USA	R. Lipp	P	Obtained				P	Obtained	
K0274	A5736	D-93	*Xanthomonas*	*axonopodis*		*dieffenbachiae*		Spathiphyllum	Hawaii, USA	R. Lipp	P	Obtained				A	NotObtained	
K0275	A5737	D-99	*Xanthomonas*	*axonopodis*		*dieffenbachiae*		Xanthosoma	Florida, USA	R. Lipp	P	NotObtained				P	NotObtained	
K0276	A5738	D-102	*Xanthomonas*	*axonopodis*		*dieffenbachiae*		Anthurium	Hawaii, USA	R. Lipp	P	Obtained				P	Obtained	
K0277	A5739	D119	*Xanthomonas*	*axonopodis*		*dieffenbachiae*		Anthurium	Hawaii, USA	R. Lipp	P	NotObtained				P	Obtained	

K0278	A5740	D123	*Xanthomonas*	*axonopodis*		*dieffenbachiae*		Anthurium	Hawaii, USA	R. Lipp	P	NotObtained				P	Obtained	
K0279	A5741	D147	*Xanthomonas*	*axonopodis*		*dieffenbachiae*		Anthurium	Hawaii, USA	R. Lipp	P	NotObtained				P	Obtained	
K0280	A5742	R-D-14-1	*Xanthomonas*	*axonopodis*		*dieffenbachiae*		Anthurium	Hawaii, USA	R. Lipp	P	Obtained				P	Obtained	
K0281	A5743	A-755-1	*Xanthomonas*	*axonopodis*		*dieffenbachiae*		Anthurium	Hawaii, USA	R. Lipp	P	Obtained				P	Obtained	
K0282	A1105	QR-27	*Xanthomonas*	*campestris*		*aberrans*		Cabbage	California, USA	M. P. Starr	P	Obtained				P	Obtained	
K0283	A2692	PDDCC574	*Xanthomonas*	*campestris*		*incanae*		Methiola incana	California, USA	M. P. Starr	P	Obtained				P	Obtained	
K0284	A2693	PDDCC1404	*Xanthomonas*	*campestris*		*raphani*		Radish	California, USA	N.J. Palleroni	P	Obtained				P	NotObtained	
K0285	A3591		*Xanthomonas*	*campestris*		*campestris*		Cabbage	Panama	A. Alvarez	P	Obtained				P	Obtained	
K0286	A3592		*Xanthomonas*	*campestris*		*campestris*		Cabbage	Panama	A. Alvarez	P	Obtained				P	Obtained	
K0287	A3593		*Xanthomonas*	*campestris*		*campestris*		Cabbage	Panama	A. Alvarez	P	Obtained				P	Obtained	
K0288	A3594		*Xanthomonas*	*campestris*		*campestris*		Cabbage	Panama	A. Alvarez	P	Obtained				P	Obtained	
K0289	A3596		*Xanthomonas*	*campestris*		*campestris*		Broccoli	Panama	A. Alvarez	P	Obtained				P	Obtained	
K0290	A3597		*Xanthomonas*	*campestris*		*campestris*		Brussel sprouts	Panama	A. Alvarez	P					P	Obtained	
K0291	A3598		*Xanthomonas*	*campestris*		*campestris*		Brussel sprouts	Panama	A. Alvarez	P					P	Obtained	
K0292	A4844	ChiKK-1	*Xanthomonas*	*campestris*		*campestris*		crucifer	Chitwan Khairani, Nepal	D. Shakya	P					P	Obtained	
K0293	A4845	ChiKK-2	*Xanthomonas*	*campestris*		*campestris*		crucifer	Chitwan Khairani, Nepal	D. Shakya	P					P	Obtained	
K0294	A4846	NapC-1	*Xanthomonas*	*campestris*		*campestris*		Cabbage	Nawalparasi, Nepal	D. Shakya	P					P	Obtained	
K0295	A4898	B4 10TB-19	*Xanthomonas*	*axonopodis*		*vitians*		Lettuce	Hawaii, USA	J. Cho	P					P	Obtained	
K0296	A4967	Box24-2	*Xanthomonas*	*campestris*		*campestris*		Cabbage	Washington, USA	R. Gabrielson	P					P	Obtained	
K0297	A4968	Box24-3	*Xanthomonas*	*campestris*		*campestris*		Cabbage	Washington, USA	R. Gabrielson	P					P	Obtained	

K0298	A4970	Box 24-5	*Xanthomonas*	*campestris*		*campestris*		Cabbage	Washington, USA	R. Gabrielson	P					P	Obtained	
K0299	A4971	Box 24-6	*Xanthomonas*	*campestris*		*campestris*		Cabbage	Washington, USA	R. Gabrielson	P					P	Obtained	
K0300	A4972	Box 24-7	*Xanthomonas*	*campestris*		*campestris*		Cabbage	Washington, USA	R. Gabrielson	P					P	Obtained	
K0301	A4973	Box 24-8	*Xanthomonas*	*campestris*		*campestris*		Cabbage	Washington, USA	R. Gabrielson	P					P	Obtained	
K0302	A4975	Box 24-10	*Xanthomonas*	*campestris*		*campestris*		Cabbage	Washington, USA	R. Gabrielson	P					P	Obtained	
K0303	A4976	Box 24-11	*Xanthomonas*	*campestris*		*campestris*		Cabbage	Washington, USA	R. Gabrielson	P					P	Obtained	
K0304	A4978	Box 24-13	*Xanthomonas*	*campestris*		*campestris*		Cabbage	Washington, USA	R. Gabrielson	P					P	Obtained	
K0305	A4979	Box 24-14	*Xanthomonas*	*campestris*		*campestris*		Cabbage	Washington, USA	R. Gabrielson	P					P	Obtained	
K0306	A4980	Box 24-15	*Xanthomonas*	*campestris*		*campestris*		Cabbage	Washington, USA	R. Gabrielson	P					P	Obtained	
K0307	A4981	Box 24-16	*Xanthomonas*	*campestris*		*campestris*		Cabbage	Washington, USA	R. Gabrielson	P					P	Obtained	
K0308	A4982	Box 24-17	*Xanthomonas*	*campestris*		*campestris*		Cabbage	Washington, USA	R. Gabrielson	P					P	Obtained	
K0309	A4983	Box 24-18	*Xanthomonas*	*campestris*		*campestris*		Cabbage	Washington, USA	R. Gabrielson	P					P	Obtained	
K0310	A4985	Box 24-20	*Xanthomonas*	*campestris*		*campestris*		Cabbage	Washington, USA	R. Gabrielson	P					P	Obtained	
K0311	A1779	XCO	*Xanthomonas*	*oryzae*		*oryzae*		Rice	California, USA	I. W. Buddenhagen	P					P	Obtained	
K0312	A2214	JBG1	*Xanthomonas*	*oryzae*		*oryzae*		Rice	Texas, USA	C. Gonzalez	P					A	NotObtained	
K0313	A2215	JBG2	*Xanthomonas*	*oryzae*		*oryzae*		Rice	Texas, USA	C. Gonzalez	P					A	NotObtained	
K0314	A2216	JBG3	*Xanthomonas*	*oryzae*		*oryzae*		Rice	Texas, USA	C. Gonzalez	P					A	NotObtained	
K0315	A2220	JBG7	*Xanthomonas*	*oryzae*		*oryzae*		Rice	Texas, USA	C. Gonzalez	P					P	Obtained	
K0316	A2221	JBG8	*Xanthomonas*	*oryzae*		*oryzae*		Rice	Texas, USA	C. Gonzalez	P					P	Obtained	
K0317	A2222	JBG9	*Xanthomonas*	*oryzae*		*oryzae*		Rice	Texas, USA	C. Gonzalez	P					P	NotObtained	

K0318	A2223	JBG10	*Xanthomonas*	*oryzae*		*oryzae*		Rice	Texas, USA	C. Gonzalez	P					P	Obtained	
K0319	A2224	XO16	*Xanthomonas*	*oryzae*		*oryzae*		Rice	Hawaii, USA	M. Grainge	P					P	Obtained	
K0320	A2226	XO22	*Xanthomonas*	*oryzae*		*oryzae*		Rice	Hawaii, USA	M. Grainge	P					A	NotObtained	
K0321	A2235	PXO86	*Xanthomonas*	*oryzae*		*oryzae*		Rice	Philippines	T. W. Mew	P					P	Obtained	
K0322	A2246	JXO T7133	*Xanthomonas*	*oryzae*		*oryzae*		Rice	Colorado	J. Leach	P					P	Obtained	
K0323	A2262	LARU 87-6	*Xanthomonas*	*oryzae*		*oryzae*		Rice	Colorado	J. Leach	P					A	NotObtained	
K0324	A2263	LARU 87-8	*Xanthomonas*	*oryzae*		*oryzae*		Rice	Colorado	J. Leach	P					A	NotObtained	
K0325	A2462	PXO1	*Xanthomonas*	*oryzae*		*oryzae*		Rice	Philippines	T. W. Mew	P					P	Obtained	
K0326	A2217	JBG4	*Xanthomonas*	*oryzae*		*oryzae*		Rice	Texas, USA	C. Gonzalez	P					A	NotObtained	
K0327	A2218	JBG5	*Xanthomonas*	*oryzae*		*oryzae*		Rice	Texas, USA	C. Gonzalez	P					P	Obtained	
K0328	A2219	JBG6	*Xanthomonas*	*oryzae*		*oryzae*		Rice	Texas, USA	C. Gonzalez	P					P	Obtained	
K0329	A5336	XCC406	*Xanthomonas*	*citri*	*citri*			Citrus	Florida, USA	T. Schubert	P					P	Obtained	
K0330	A5338	X2002-01035	*Xanthomonas*	*citri*	*citri*			Citrus	Florida, USA	T. Schubert	P					P	NotObtained	
K0331	A5339	XS2001-00004	*Xanthomonas*	*alfalfae*	*citrumelonis*			Citrus	Florida, USA	T. Schubert	P					P	Obtained	
K0332	A5340	XC2005-00252	*Xanthomonas*	*alfalfae*	*citrumelonis*			Citrus	Florida, USA	T. Schubert	P					P	Obtained	
K0333	A5344	X2004-1469	*Xanthomonas*	*citri*	*citri*			Citrus	Florida, USA	T. Schubert	P					P	NotObtained	
K0334	A638-1	A638-1	*Xanthomonas*	*euvesicatoria*				Pepper	Hawaii, USA	A. Alvarez	P					P	Obtained	
K0335	A1780	A571-1	*Xanthomonas*	*euvesicatoria*				Pepper	Hawaii, USA	B. S. Kim	P					P	Obtained	
K0336	A1781	Poamoho	*Xanthomonas*	*euvesicatoria*				Pepper	Hawaii, USA	B. S. Kim	P					P	Obtained	
K0337	A1782	Fukuyama Kahuku	*Xanthomonas*	*euvesicatoria*				Pepper	Hawaii, USA	B. S. Kim	P					P	Obtained	
K0338	A1785	EWCII Com.gard	*Xanthomonas*	*euvesicatoria*				Pepper	Hawaii, USA	B. S. Kim	P					A	NotObtained	
K0339	A1786	Kawai I Uni. Chi	*Xanthomonas*	*euvesicatoria*				Pepper	Hawaii, USA	B. S. Kim	P					P	Obtained	
K0340	A5335	XCC290	*Xanthomonas*	*citri*	*citri*			Citrus	Florida, USA	T. Schubert	P					A	NotObtained	
K0341	A5343	XC2002-	*Xanthomonas*	*citri*	*citri*			Citrus	Florida,	T. Schubert	P					A	NotObtained	

		00010							USA									
K0342	A1787	Kawai II Uni.Chi.	*Xanthomonas*	*euvesicatoria*				Tomato	Hawaii, USA	B. S. Kim	P					P	Obtained	
K0343	A5716	82-7a	*Xanthomonas*	*euvesicatoria*				Tomato	Florida, USA	J. B. Jones	P					P	Obtained	
K0344	A5744	82-16	*Xanthomonas*	*euvesicatoria*				Tomato	Florida, USA	J. B. Jones	P					P	Obtained	
K0345	A5745	83-13b	*Xanthomonas*	*euvesicatoria*				Tomato	Florida, USA	J. B. Jones	P					P	Obtained	
K0346	A5746	83-14	*Xanthomonas*	*euvesicatoria*				Tomato	Florida, USA	J. B. Jones	P					P	Obtained	
K0347	A3477	XVP26	*Xanthomonas*	*euvesicatoria*				Tomato	Florida, USA	J. B. Jones	P					P	Obtained	
K0348	A3478	XVT8	*Xanthomonas*	*euvesicatoria*				Tomato	Florida, USA	J. B. Jones	P					P	Obtained	
K0349	A3479	XVP29	*Xanthomonas*	*euvesicatoria*				Tomato	Florida, USA	J. B. Jones	P					P	Obtained	
K0350	A3480	XVT20	*Xanthomonas*	*euvesicatoria*				Tomato	Florida, USA	J. B. Jones	P					P	Obtained	
K0351	A3481	XVT14	*Xanthomonas*	*euvesicatoria*				Tomato	Florida, USA	J. B. Jones	P					A	NotObtained	
K0352	A3483	XVT18	*Xanthomonas*	*euvesicatoria*				Tomato	Florida, USA	J. B. Jones	P					P	Obtained	
K0353	A3484	XVT5	*Xanthomonas*	*euvesicatoria*				Tomato	Florida, USA	J. B. Jones	P					P	Obtained	
K0354	A5087	A-3	*Xanthomonas*	*campestris*		*campestris*		Cabbage	Georgia, USA	R. Gitaitis	P					P	Obtained	
K0355	A5058	A-4	*Xanthomonas*	*campestris*		*campestris*		Cabbage	Georgia, USA	R. Gitaitis	P					P	Obtained	
K0356	A5060	A-6	*Xanthomonas*	*campestris*		*campestris*		Cabbage	Georgia, USA	R. Gitaitis	P					P	Obtained	
K0357	A5088	A-7	*Xanthomonas*	*campestris*		*campestris*		Cabbage	Georgia, USA	R. Gitaitis	P					P	Obtained	
K0358	A5089	A-10	*Xanthomonas*	*campestris*		*campestris*		Cabbage	Georgia, USA	R. Gitaitis	P					P	Obtained	

K0359	A5061	B-1	*Xanthomonas*	*campestris*		*campestris*		Cabbage	Georgia,<br>USA	R. Gitaitis	P					P	Obtained	
K0360	A5062	B-2	*Xanthomonas*	*campestris*		*campestris*		Cabbage	Georgia,<br>USA	R. Gitaitis	P					P	Obtained	
K0361	A5063	B-3	*Xanthomonas*	*campestris*		*campestris*		Cabbage	Georgia,<br>USA	R. Gitaitis	P					P	Obtained	
K0362	A5372	B-4	*Xanthomonas*	*campestris*		*campestris*		Cabbage	Georgia,<br>USA	R. Gitaitis	P					P	Obtained	
K0363	A5064	B-7	*Xanthomonas*	*campestris*		*campestris*		Cabbage	Georgia,<br>USA	R. Gitaitis	P					P	Obtained	
K0364	A5067	C-1	*Xanthomonas*	*campestris*		*campestris*		Cabbage	Georgia,<br>USA	R. Gitaitis	P					P	Obtained	
K0365	A5092	C-2	*Xanthomonas*	*campestris*		*campestris*		Cabbage	Georgia,<br>USA	R. Gitaitis	P					P	Obtained	
K0366	A5093	C-3	*Xanthomonas*	*campestris*		*campestris*		Cabbage	Georgia,<br>USA	R. Gitaitis	P					P	Obtained	
K0367	A5068	C-4	*Xanthomonas*	*campestris*		*campestris*		Cabbage	Georgia,<br>USA	R. Gitaitis	P					P	Obtained	
K0368	A5069	C-6	*Xanthomonas*	*campestris*		*campestris*		Cabbage	Georgia,<br>USA	R. Gitaitis	P					P	Obtained	
K0369	A5070	C-7	*Xanthomonas*	*campestris*		*campestris*		Cabbage	Georgia,<br>USA	R. Gitaitis	P					P	Obtained	
K0370	A5094	C-8	*Xanthomonas*	*campestris*		*campestris*		Cabbage	Georgia,<br>USA	R. Gitaitis	P					P	Obtained	
K0371	A5373	D-2	*Xanthomonas*	*campestris*		*campestris*		Cabbage	Georgia,<br>USA	R. Gitaitis	P					P	Obtained	
K0372	A5095	D-3	*Xanthomonas*	*campestris*		*campestris*		Cabbage	Georgia,<br>USA	R. Gitaitis	P					P	Obtained	
K0373	A5072	D-5	*Xanthomonas*	*campestris*		*campestris*		Cabbage	Georgia,<br>USA	R. Gitaitis	P					P	Obtained	
K0374	A5073	D-7	*Xanthomonas*	*campestris*		*campestris*		Cabbage	Georgia,<br>USA	R. Gitaitis	P					P	Obtained	
K0375	A5066	B-9	*Xanthomonas*	*campestris*		*campestris*		Cabbage	Georgia,<br>USA	R. Gitaitis	P					P	Obtained	
K0376	A1105a	QR-27a	*Xanthomonas*	*campestris*		*aberrans*		Cabbage	California,<br>USA	M. P. Starr	P					P	Obtained	

K0377	A2690	PDDCC 4805	*Xanthomonas*	*campestris*		*aberrans*		Cabbage	New Zealand	J. Young	P					P	Obtained	
K0378	A5115	E-1	*Xanthomonas*	*campestris*		*campestris*		Cabbage	Georgia, USA	R. Gitaitis	P					A	NotObtained	
K0379	A5074	E-2	*Xanthomonas*	*campestris*		*campestris*		Cabbage	Georgia, USA	R. Gitaitis	P					P	Obtained	
K0380	A5075	E-3	*Xanthomonas*	*campestris*		*campestris*		Cabbage	Georgia, USA	R. Gitaitis	P					P	Obtained	
K0381	A5076	E-5	*Xanthomonas*	*campestris*		*campestris*		Cabbage	Georgia, USA	R. Gitaitis	P					P	Obtained	
K0382	A5079	F-5	*Xanthomonas*	*campestris*		*campestris*		Cabbage	Georgia, USA	R. Gitaitis	P					A	NotObtained	
K0383	A5718	F-8	*Xanthomonas*	*campestris*		*campestris*		Cabbage	Georgia, USA	R. Gitaitis	P					P	Obtained	
K0384	A5097	F-10	*Xanthomonas*	*campestris*		*campestris*		Cabbage	Georgia, USA	R. Gitaitis	P					P	Obtained	
K0385	A1746	A 518-1	*Clavibacter*	*michiganensis*	*michiganensis*			Tomato	Hawaii, USA	W.Chun						P	Obtained	
K0386	A1949	B-125	*Clavibacter*	*michiganensis*	*michiganensis*			Tomato	California, USA	J. Watterson						P	Obtained	
K0387	A2069	CM95	*Clavibacter*	*michiganensis*	*michiganensis*			Tomato	Ohio, USA	S. Nameth						P	Obtained	
K0388	A2071	CM97	*Clavibacter*	*michiganensis*	*michiganensis*			Tomato	Ohio, USA	S. Nameth						P	Obtained	
K0389	A2072	CM98	*Clavibacter*	*michiganensis*	*michiganensis*			Tomato	Ohio, USA	S. Nameth						P	Obtained	
K0390	A2073	CM99	*Clavibacter*	*michiganensis*	*michiganensis*			Tomato	Ohio, USA	S. Nameth						P	Obtained	
K0391	A2074	CM100	*Clavibacter*	*michiganensis*	*michiganensis*			Tomato	Ohio, USA	S. Nameth						P	Obtained	
K0392	A2297	CM5	*Clavibacter*	*michiganensis*	*michiganensis*			Tomato	Ohio, USA	D. Coplin						P	Obtained	
K0393	A2626	C12	*Clavibacter*	*michiganensis*	*michiganensis*			Tomato	California, USA	H.Bolkan						P	Obtained	
K0394	A2627	C19	*Clavibacter*	*michiganensis*	*michiganensis*			Tomato	California, USA	H.Bolkan						P	Obtained	

K0395	A2644	S44	*Clavibacter*	*michiganensis*	*michiganensis*			Tomato	California, USA	H.Bolkan						P	Obtained	
K0396	A2646	S51	*Clavibacter*	*michiganensis*	*michiganensis*			Tomato	California, USA	H.Bolkan						P	Obtained	
K0397	A2647	S52	*Clavibacter*	*michiganensis*	*michiganensis*			Tomato	California, USA	H.Bolkan						P	Obtained	
K0398	A2648	S53	*Clavibacter*	*michiganensis*	*michiganensis*			Tomato	California, USA	H.Bolkan						P	Obtained	
K0399	A2696	CM36	*Clavibacter*	*michiganensis*	*michiganensis*			Tomato	North Carolina, USA	J. Beagle-Ristiano						P	Obtained	
K0400	A2697	CM33	*Clavibacter*	*michiganensis*	*michiganensis*			Tomato	North Carolina, USA	J. Beagle-Ristiano						P	Obtained	
K0401	A2698	CM Finley	*Clavibacter*	*michiganensis*	*michiganensis*			Tomato	North Carolina, USA	J. Beagle-Ristiano						P	Obtained	
K0402	A2700	CM Kuykendall	*Clavibacter*	*michiganensis*	*michiganensis*			Tomato	North Carolina, USA	J. Beagle-Ristiano						P	Obtained	
K0403	A2701	CM Leatherwood	*Clavibacter*	*michiganensis*	*michiganensis*			Tomato	North Carolina, USA	J. Beagle-Ristiano						P	Obtained	
K0404	A3994	DR73	*Clavibacter*	*michiganensis*	*michiganensis*			Tomato	Iowa, USA	E. Braun						P	Obtained	
K0405	A3995	DR60-R1	*Clavibacter*	*michiganensis*	*michiganensis*			Tomato	Iowa, USA	E. Braun						P	Obtained	
K0406	A3997	1(A)	*Clavibacter*	*michiganensis*	*michiganensis*			Tomato	Ohio, USA	R. Clevenstine						P	Obtained	
K0407	A3998	8(A-B)	*Clavibacter*	*michiganensis*	*michiganensis*			Tomato	Ohio, USA	R. Clevenstine						P	Obtained	
K0408	A3999	17	*Clavibacter*	*michiganensis*	*michiganensis*			Tomato	Ohio, USA	R. Clevenstine						P	Obtained	
K0409	A4000	75	*Clavibacter*	*michiganensis*	*michiganensis*			Tomato	Ohio, USA	R. Clevenstine						P	Obtained	
K0410	A4001	72	*Clavibacter*	*michiganensis*	*michiganensi*			Tomato	Ohio, USA	R. Clevenstine						P	Obtained	

					s													
K0411	A4002	57	*Clavibacter*	*michiganensis*	*michiganensis*			Tomato	Ohio, USA	R. Clevenstine						P	Obtained	
K0412	A4003	73	*Clavibacter*	*michiganensis*	*michiganensis*			Tomato	Ohio, USA	R. Clevenstine						P	Obtained	
K0413	A4010	18(E)	*Clavibacter*	*michiganensis*	*michiganensis*			Tomato	Ohio, USA	R. Clevenstine						P	Obtained	
K0414	A4012	34C	*Clavibacter*	*michiganensis*	*michiganensis*			Tomato	Ohio, USA	R. Clevenstine						P	Obtained	
K0415	A4013	7	*Clavibacter*	*michiganensis*	*michiganensis*			Tomato	Ohio, USA	R. Clevenstine						P	Obtained	
K0416	A4014	24	*Clavibacter*	*michiganensis*	*michiganensis*			Tomato	Ohio, USA	R. Clevenstine						P	Obtained	
K0417	A4015	9	*Clavibacter*	*michiganensis*	*michiganensis*			Tomato	Ohio, USA	R. Clevenstine						P	Obtained	
K0418	A4016	29	*Clavibacter*	*michiganensis*	*michiganensis*			Tomato	Ohio, USA	R. Clevenstine						P	Obtained	
K0419	A4017	41	*Clavibacter*	*michiganensis*	*michiganensis*			Tomato	Ohio, USA	R. Clevenstine						P	Obtained	
K0420	A4019	4	*Clavibacter*	*michiganensis*	*michiganensis*			Tomato	Ohio, USA	R. Clevenstine						P	Obtained	
K0421	A4020	2(C)	*Clavibacter*	*michiganensis*	*michiganensis*			Tomato	Ohio, USA	R. Clevenstine						P	Obtained	
K0422	A4021	70	*Clavibacter*	*michiganensis*	*michiganensis*			Tomato	Ohio, USA	R. Clevenstine						P	Obtained	
K0423	A4022	5	*Clavibacter*	*michiganensis*	*michiganensis*			Tomato	Ohio, USA	R. Clevenstine						P	Obtained	
K0424	A4023	19	*Clavibacter*	*michiganensis*	*michiganensis*			Tomato	Ohio, USA	R. Clevenstine						P	Obtained	
K0425	A4024	10	*Clavibacter*	*michiganensis*	*michiganensis*			Tomato	Ohio, USA	R. Clevenstine						P	Obtained	
K0426	A4025	27	*Clavibacter*	*michiganensis*	*michiganensis*			Tomato	Ohio, USA	R. Clevenstine						P	Obtained	
K0427	A4026	20	*Clavibacter*	*michiganensis*	*michiganensis*			Tomato	Ohio, USA	R. Clevenstine						P	Obtained	

K0428	A4027	6(A)	*Clavibacter*	*michiganensis*	*michiganensis*			Tomato	Ohio, USA	R. Clevenstine						P	Obtained	
K0429	A4028	38	*Clavibacter*	*michiganensis*	*michiganensis*			Tomato	Ohio, USA	R. Clevenstine						P	Obtained	
K0430	A4029	22	*Clavibacter*	*michiganensis*	*michiganensis*			Tomato	Ohio, USA	R. Clevenstine						P	Obtained	
K0431	A4030	12	*Clavibacter*	*michiganensis*	*michiganensis*			Tomato	Ohio, USA	R. Clevenstine						P	Obtained	
K0432	A4031	37	*Clavibacter*	*michiganensis*	*michiganensis*			Tomato	Ohio, USA	R. Clevenstine						P	Obtained	
K0433	A4032	33	*Clavibacter*	*michiganensis*	*michiganensis*			Tomato	Ohio, USA	R. Clevenstine						P	Obtained	
K0434	A4034	28	*Clavibacter*	*michiganensis*	*michiganensis*			Tomato	Ohio, USA	R. Clevenstine						P	Obtained	
K0435	A4035	74	*Clavibacter*	*michiganensis*	*michiganensis*			Tomato	Ohio, USA	R. Clevenstine						P	Obtained	
K0436	A4036	16	*Clavibacter*	*michiganensis*	*michiganensis*			Tomato	Ohio, USA	R. Clevenstine						P	Obtained	
K0437	A4037	1(C)	*Clavibacter*	*michiganensis*	*michiganensis*			Tomato	Ohio, USA	R. Clevenstine						P	Obtained	
K0438	A4038	44	*Clavibacter*	*michiganensis*	*michiganensis*			Tomato	Ohio, USA	R. Clevenstine						P	Obtained	
K0439	A4042	DR59	*Clavibacter*	*michiganensis*	*michiganensis*			Tomato	Iowa, USA	E. Braun						P	Obtained	
K0440	A4043	BR4	*Clavibacter*	*michiganensis*	*michiganensis*			Tomato	Iowa, USA	E. Braun						P	Obtained	
K0441	A4047	11	*Clavibacter*	*michiganensis*	*michiganensis*			Tomato	Ohio, USA	R. Clevenstine						P	Obtained	
K0442	A4048	35	*Clavibacter*	*michiganensis*	*michiganensis*			Tomato	Ohio, USA	R. Clevenstine						P	Obtained	
K0443	A4049	45	*Clavibacter*	*michiganensis*	*michiganensis*			Tomato	Ohio, USA	R. Clevenstine						P	Obtained	
K0444	A4050	25	*Clavibacter*	*michiganensis*	*michiganensis*			Tomato	Ohio, USA	R. Clevenstine						P	Obtained	
K0445	A4051	69	*Clavibacter*	*michiganensis*	*michiganensis*			Tomato	Ohio, USA	R. Clevenstine						P	Obtained	

K0446	A4052	6(C)	*Clavibacter*	*michiganensis*	*michiganensis*			Tomato	Ohio, USA	R. Clevenstine						P	Obtained	
K0447	A4053	1(H)	*Clavibacter*	*michiganensis*	*michiganensis*			Tomato	Ohio, USA	R. Clevenstine						P	Obtained	
K0448	A4054	18(D)	*Clavibacter*	*michiganensis*	*michiganensis*			Tomato	Ohio, USA	R. Clevenstine						P	Obtained	
K0449	A4056	26	*Clavibacter*	*michiganensis*	*michiganensis*			Tomato	Ohio, USA	R. Clevenstine						P	Obtained	
K0450	A4057	34(E)	*Clavibacter*	*michiganensis*	*michiganensis*			Tomato	Ohio, USA	R. Clevenstine						P	Obtained	
K0451	A4059	36	*Clavibacter*	*michiganensis*	*michiganensis*			Tomato	Ohio, USA	R. Clevenstine						P	Obtained	
K0452	A4061	8(C)	*Clavibacter*	*michiganensis*	*michiganensis*			Tomato	Ohio, USA	R. Clevenstine						P	Obtained	
K0453	A4065	21	*Clavibacter*	*michiganensis*	*michiganensis*			Tomato	Ohio, USA	R. Clevenstine						P	Obtained	
K0454	A4067	14	*Clavibacter*	*michiganensis*	*michiganensis*			Tomato	Ohio, USA	R. Clevenstine						P	Obtained	
K0455	A4068	43	*Clavibacter*	*michiganensis*	*michiganensis*			Tomato	Ohio, USA	R. Clevenstine						P	Obtained	
K0456	A4069	2(A)	*Clavibacter*	*michiganensis*	*michiganensis*			Tomato	Ohio, USA	R. Clevenstine						P	Obtained	
K0457	A4504		*Clavibacter*	*michiganensis*	*michiganensis*			Tomato	Ohio, USA	D. Maddox						P	Obtained	
K0458	A4505		*Clavibacter*	*michiganensis*	*michiganensis*			Tomato	Ohio, USA	D. Maddox						P	Obtained	
K0459	A4506		*Clavibacter*	*michiganensis*	*michiganensis*			Tomato	Ohio, USA	D. Maddox						P	Obtained	
K0460	A4589	cmm018	*Clavibacter*	*michiganensis*	*michiganensis*			Tomato	Washington, USA	M. Derie						P	Obtained	
K0461	A4591	cmm024	*Clavibacter*	*michiganensis*	*michiganensis*			Tomato	Washington, USA	M. Derie						P	Obtained	
K0462	A4596	cmm035	*Clavibacter*	*michiganensis*	*michiganensis*			Tomato	Washington, USA	M. Derie						P	Obtained	
K0463	A4597	cmm037	*Clavibacter*	*michiganensis*	*michiganensis*			Tomato	Washington, USA	M. Derie						P	Obtained	

K0464	A4603	cmm055	*Clavibacter*	*michiganensis*	*michiganensis*			Tomato	Washington, USA	M. Derie						P	Obtained	
K0465	A4690	cmm 461	*Clavibacter*	*michiganensis*	*michiganensis*			Tomato	Portugal	M. S. Santos						P	Obtained	
K0466	A4691	cmm 462	*Clavibacter*	*michiganensis*	*michiganensis*			Tomato	Portugal	M. S. Santos						P	NotObtained	
K0467	A4744	IPO 500	*Clavibacter*	*michiganensis*	*michiganensis*			Tomato	UK	I. de Vries						P	Obtained	
K0468	A4745	IPO 501	*Clavibacter*	*michiganensis*	*michiganensis*			Tomato	Hungary	I. de Vries						P	Obtained	
K0469	A4746	IPO 541	*Clavibacter*	*michiganensis*	*michiganensis*			Tomato	UK	I. de Vries						P	Obtained	
K0470	A4747	IPO 542	*Clavibacter*	*michiganensis*	*michiganensis*			Tomato	Italy	I. de Vries						P	Obtained	
K0471	A4749	IPO 544	*Clavibacter*	*michiganensis*	*michiganensis*			Tomato	Hungary	I. de Vries						P	Obtained	
K0472	A4751	IPO 630	*Clavibacter*	*michiganensis*	*michiganensis*			Tomato	Netherlands	I. de Vries						P	Obtained	
K0473	A4752	IPO 1799	*Clavibacter*	*michiganensis*	*michiganensis*			Tomato	Chile	G. Hoyos						P	Obtained	
K0474	A4753	N 197	*Clavibacter*	*michiganensis*	*michiganensis*			Tomato	California, USA	G. Hoyos						P	Obtained	
K0475	A4754	N 198	*Clavibacter*	*michiganensis*	*michiganensis*			Tomato	California, USA	G. Hoyos						P	Obtained	
K0476	A4755	N 202A	*Clavibacter*	*michiganensis*	*michiganensis*			Tomato	Chile	G. Hoyos						P	Obtained	
K0477	A4756	N202B	*Clavibacter*	*michiganensis*	*michiganensis*			Tomato	Chile	G. Hoyos						P	Obtained	
K0478	A4757	N 211	*Clavibacter*	*michiganensis*	*michiganensis*			Tomato	China	G. Hoyos						P	Obtained	
K0479	A4759	N 213	*Clavibacter*	*michiganensis*	*michiganensis*			Tomato	Chile	G. Hoyos						P	Obtained	
K0480	A4769	N 713P	*Clavibacter*	*michiganensis*	*michiganensis*			Tomato	Chile	G. Hoyos						P	Obtained	
K0481	A1042	A1042-2	*Dickeya*	*sp.*				Philodendron	Hawaii, USA	A. Alvarez	P		P			P	Obtained	

K0482	A4584	9X	*Pectobacterium*	*carotovorum*				Aglaonema sp.	Hawaii, USA	R. Arias	P	Obtained	P			P	Obtained	
K0483	A5263	1-1A	*Dickeya*	*sp.*				Pineapple	Hawaii, USA	A. Alvarez	P	Obtained	P			P	Obtained	
K0484	A5264	1-2A	*Dickeya*	*sp.*				Pineapple	Hawaii, USA	A. Alvarez	P	Obtained	P			P	Obtained	
K0485	A5265	1-3A	*Dickeya*	*sp.*				Pineapple	Hawaii, USA	A. Alvarez	P	Obtained	P			P	Obtained	
K0486	A5266	1-4A	*Dickeya*	*sp.*				Pineapple	Hawaii, USA	A. Alvarez	P		P			P	Obtained	
K0487	A5267	1-5A	*Dickeya*	*sp.*				Pineapple	Hawaii, USA	A. Alvarez	P	Obtained	P			P	Obtained	
K0488	A5268	1-5B	*Dickeya*	*sp.*				Pineapple	Hawaii, USA	A. Alvarez	P		P			P	Obtained	
K0489	A5269	1-6A	*Dickeya*	*sp.*				Pineapple	Hawaii, USA	A. Alvarez	P	Obtained	P			P	Obtained	
K0490	A5270	1-7B	*Dickeya*	*sp.*				Pineapple	Hawaii, USA	A. Alvarez	P	Obtained	P			P	Obtained	
K0491	A5271	1-8B	*Dickeya*	*sp.*				Pineapple	Hawaii, USA	A. Alvarez	P	Obtained	P			P	Obtained	
K0492	A5272	1-9C	*Dickeya*	*sp.*				Pineapple	Hawaii, USA	A. Alvarez	A	Obtained	P			P	Obtained	
K0493	A5274	1-10C	*Dickeya*	*sp.*				Pineapple	Hawaii, USA	A. Alvarez	P	Obtained	P			P	NotObtained	
K0494	A5277	1-#18	*Dickeya*	*sp.*				Irrigation water	Hawaii, USA	A. Alvarez	P		P			P	Obtained	
K0495	A5292	7-1	*Dickeya*	*sp.*				Pineapple	Hawaii, USA	G. Taniguchi	P	Obtained	P			P	Obtained	
K0496	A5293	7-2	*Dickeya*	*sp.*				Pineapple	Hawaii, USA	G. Taniguchi	P	Obtained	P			P	Obtained	
K0497	A5303	3-2	*Dickeya*	*sp.*				Pineapple	Hawaii, USA	A. Alvarez	P	Obtained	P			P	Obtained	
K0498	A5304	3-3	*Dickeya*	*sp.*				Pineapple	Hawaii, USA	A. Alvarez	P	Obtained	P			P	Obtained	
K0499	A5306	3-5	*Dickeya*	*sp.*				Pineapple	Hawaii, USA	A. Alvarez	P	Obtained	P			P	Obtained	
K0500	A5307	3-6	*Dickeya*	*sp.*				Pineapple	Hawaii, USA	A. Alvarez	P	Obtained	A			P	Obtained	
K0501	A5308	3-7	*Dickeya*	*sp.*				Pineapple	Hawaii, USA	A. Alvarez	P	Obtained	P			P	Obtained	
K0502	A5309	3-8	*Dickeya*	*sp.*				Pineapple	Hawaii, USA	A. Alvarez	P	Obtained	P			P	Obtained	
K0503	A5310	3-9	*Dickeya*	*sp.*				Pineapple	Hawaii, USA	A. Alvarez	P		P			P	Obtained	
K0504	A5311	3-10	*Dickeya*	*sp.*				Pineapple	Hawaii, USA	A. Alvarez	P	Obtained	P			P	Obtained	
K0505	A1955	D5	*Dickeya*	*sp.*				Maize	Missouri, USA	A. Chatterjee	A	Obtained	P			P	NotObtained	
K0506	A5371	CC26	*Pectobacterium*	*carotovorum*				Aglaonema sp.	Hawaii, USA	A. Alvarez	P		A			P	Obtained	
K0507	A5278	1-#21	*Pectobacterium*	*sp.*				Irrigation water	Hawaii, USA	A. Alvarez	A		A			P	Obtained	
K0508	A5280	1-#31	*Pectobacteriu*	*sp.*				Irrigation water	Hawaii, USA	A. Alvarez	P		A			P	Obtained	

			m															
K0509	A5299	9-1	*Pectobacterium*	*carotovorum*				Pineapple	Hawaii, USA	A. Alvarez	A		A			A	NotObtained	P
K0510	A3308A	PD738	*Pectobacterium*	*atrosepticum*				Potato	Netherlands	J. Janse	A		A			A	NotObtained	A
K0511	A3308B	PD738	*Pectobacterium*	*atrosepticum*				Potato	Netherlands	J. Janse	A		A			A	NotObtained	A
K0512	A5359	Ecc	*Pectobacterium*	*carotovorum*				Papaya (Carica papaya)	Hawaii, USA	O. Kwok	A		A			A	NotObtained	A
K0513	A5365	2B	*Pectobacterium*	*carotovorum*				Aglaonema sp.	Hawaii, USA	R. Arias	A		A			A	NotObtained	A
K0514	A5366	2C	*Pectobacterium*	*carotovorum*				Aglaonema sp.	Hawaii, USA	R. Arias	A		A			A	NotObtained	A
K0515	A5367	4C	*Pectobacterium*	*carotovorum*				Aglaonema sp.	Hawaii, USA	R. Arias	A		A			A	NotObtained	A
K0516	A5368	5X	*Pectobacterium*	*carotovorum*				Aglaonema sp.	Hawaii, USA	R. Arias	A		A			SMEAR	Obtained	P
K0517	A5369	8X	*Pectobacterium*	*carotovorum*				Aglaonema sp.	Hawaii, USA	R. Arias	P		A			P	Obtained	
K0518	A5347	2-2b	*Pectobacterium*	*sp.*				Aglaonema sp.	Hawaii, USA	R. Arias	P		A			P	Obtained	
K0519	A5348	2d	*Dickeya*	*sp.*				Aglaonema sp.	Hawaii, USA	R. Arias	A		A			A	NotObtained	A
K0520	A5349	5C-A red spot	*Dickeya*	*sp.*				Aglaonema sp.	Hawaii, USA	R. Arias	A		A			A	NotObtained	A
K0521	A5350	5C-B red spot	*Dickeya*	*sp.*				Aglaonema sp.	Hawaii, USA	R. Arias	A		A			A	NotObtained	A
K0522	A5352	T-15	*Pectobacterium*	*sp.*				Aglaonema sp.	Hawaii, USA	R. Arias	A		A			WEIRD	NotObtained	P
K0523	A5353	10X	*Dickeya*	*sp.*				Aglaonema sp.	Hawaii, USA	R. Arias	P		A			P	Obtained	
K0524	A5355	JF	*Pantoea*	*agglomerans*				Papaya (Carica papaya)	Hawaii, USA	M. Nelson	P		P			A	NotObtained	P
K0525	A5356	J6	*Pantoea*	*agglomerans*				Papaya (Carica papaya)	Hawaii, USA	M. Nelson	P		A			A	NotObtained	P
K0526	A5357	J8	*Pantoea*	*agglomerans*				Papaya (Carica papaya)	Hawaii, USA	M. Nelson	P		P			A	NotObtained	A

K0527	A5358	J9	*Pantoea*	*agglomerans*				Papaya (Carica papaya)	Hawaii, USA	M. Nelson	P		A			P	Obtained	P
K0528	A5360	Eh	*Pantoea*	*agglomerans*				Papaya (Carica papaya)	Hawaii, USA	O. Kwok	P		A			P	NotObtained	P
K0529	????	Eh-1, CC27	*Pantoea*	*agglomerans*				Cabbage	Hawaii, USA	A. Alvarez	P		A			P	NotObtained	P
K0530	A4141	3548	*Pantoea*	*agglomerans*				Rice	Belgium	F. van Outrye	A		A			A	NotObtained	A
K0531	A5376	14-1-1A	*Dickeya*	*sp.*				Pineapple	Hawaii, USA	A. Alvarez	P		P			P	Obtained	
K0532	A5379	14-1-1F	*Dickeya*	*sp.*				Pineapple	Hawaii, USA	A. Alvarez	P	Obtained	P			P	Obtained	
K0533	A5381	14-3-1A	*Dickeya*	*sp.*				Pineapple	Hawaii, USA	A. Alvarez	P		P			P	Obtained	
K0534	A5386	14-4-1I	*Dickeya*	*sp.*				Pineapple	Hawaii, USA	A. Alvarez	P		P			P	Obtained	
K0535	A5385	14-4-1F	*Dickeya*	*sp.*				Pineapple	Hawaii, USA	A. Alvarez	P	Obtained	P			P	Obtained	
K0536	A5387	14-4-1L	*Dickeya*	*sp.*				Pineapple	Hawaii, USA	A. Alvarez	P		P			P	Obtained	
K0537	A5390	14-8-2B	*Dickeya*	*sp.*				Pineapple	Hawaii, USA	A. Alvarez	P		P			P	Obtained	
K0538	A5392	14-8-2E	*Dickeya*	*sp.*				Pineapple	Hawaii, USA	A. Alvarez	P	Obtained	P			P	Obtained	
K0539	A5397	16-5-2A	*Dickeya*	*sp.*				Pineapple	Hawaii, USA	A. Alvarez	P	Obtained	P			P	Obtained	
K0540	A5399	16-5-1D	*Dickeya*	*sp.*				Pineapple	Hawaii, USA	A. Alvarez	P		P			P	Obtained	
K0541	A5405	16-6-2D	*Dickeya*	*sp.*				Pineapple	Hawaii, USA	A. Alvarez	P		P			P	Obtained	
K0542	A5408	16-6-2I	*Dickeya*	*sp.*				Pineapple	Hawaii, USA	A. Alvarez	P	Obtained	P			P	Obtained	
K0543	A5415	CFBP2048	*Dickeya*	*chrysanthemi*				Chrysanthemu m morifolium	USA	M. Fischer-Le Saux	P		P			P	NotObtained	P
K0544	A5416	CFPB1269	*Dickeya*	*dadantii*				Pelargonium capitatum	Comoros	M. Fischer-Le Saux	P		P			P	Obtained	
K0545	A5417	CFPB1278	*Dickeya*	*sp.*				Pineapple	Malaysia	M. Fischer-Le Saux	P	Obtained	P			P	Obtained	
K0546	A5418	CFPB1200	*Dickeya*	*dianthicola*				Carnation (Dianthus caryophyllus)	UK	M. Fischer-Le Saux	P		P			P	Obtained	
K0547	A5419	CFPB2051	*Dickeya*	*dieffenbachia e*				Dieffenbachia sp.	USA	M. Fischer-Le Saux	P		P			P	Obtained	
K0548	A5420	CFPB4178	*Dickeya*	*paradisiaca*				Musa paradisiaca	Colombia	M. Fischer-Le Saux	P		A			A	NotObtained	A
K0549	A5421	CFPB1272	*Dickeya*	*sp.*				Pineapple	Malaysia	M. Fischer-Le Saux	P	Obtained	P			P	Obtained	

K0550	A5422	CFPB2052	*Dickeya*	*zeae*				Maize	USA	M. Fischer-Le Saux	P	Obtained	P			P	Obtained	
K0551	A5423	CFPB6466	*Dickeya*	*zeae*				Pineapple	Martinique	M. Fischer-Le Saux	P		P			P	Obtained	
K0552	A5505	16-12-3W	*Dickeya*	*sp.*				Irrigation water	Hawaii, USA	A. Alvarez	P		P			P	Obtained	
K0553	A5432	16-12-3X	*Dickeya*	*sp.*				Irrigation water	Hawaii, USA	A. Alvarez	P	Obtained	P			P	Obtained	
K0554	A5433	16-12-3Y	*Dickeya*	*sp.*				Irrigation water	Hawaii, USA	A. Alvarez	P	Obtained	P			P	Obtained	
K0555	A5506	16-12-3Z	*Dickeya*	*sp.*				Irrigation water	Hawaii, USA	A. Alvarez	P		P			P	Obtained	
K0556	A5507	16-12-3AA	*Dickeya*	*sp.*				Irrigation water	Hawaii, USA	A. Alvarez	P	Obtained	P			P	Obtained	
K0557	A5442	16-12-4N	*Dickeya*	*sp.*				Irrigation water	Hawaii, USA	A. Alvarez	P		P			P	Obtained	
K0558	A5508	16-12-4O	*Dickeya*	*sp.*				Irrigation water	Hawaii, USA	A. Alvarez	P	Obtained	P			P	Obtained	
K0559	A5509	16-12-4P	*Dickeya*	*sp.*				Irrigation water	Hawaii, USA	A. Alvarez	P		P			P	Obtained	
K0560	A5510	16-12-4Q	*Dickeya*	*sp.*				Irrigation water	Hawaii, USA	A. Alvarez	P		P			P	Obtained	
K0561	A5444	16-12-5L	*Dickeya*	*sp.*				Irrigation water	Hawaii, USA	A. Alvarez	P	Obtained	P			P	Obtained	
K0562	A5511	16-12-5N	*Dickeya*	*sp.*				Irrigation water	Hawaii, USA	A. Alvarez	P	Obtained	P			P	Obtained	
K0563	A5512	16-12-5O	*Dickeya*	*sp.*				Irrigation water	Hawaii, USA	A. Alvarez	P		P			P	Obtained	
K0564	A5513	Walkin 1	*Pantoea*	*agglomerans*				Ornamental	Hawaii, USA	A. Alvarez	P		A			P	NotObtained	P
K0565	A5514	Walkin 2	*Pantoea*	*agglomerans*				Ornamental	Hawaii, USA	A. Alvarez	P		A			P	NotObtained	
K0566	A1088	QR-10	*Pectobacterium*	*atrosepticum*				Potato	Ohio, USA	M. P. Starr	A		A			A	NotObtained	A
K0567	A1089	QR-11	*Pectobacterium*	*carotovorum*				Pepper	California, USA	M. P. Starr	A		A			A	NotObtained	A
K0568	A3148	E21A	*Dickeya*	*sp.*				Lettuce	Hawaii, USA	J. Cho	P		P			P	NotObtained	
K0569	A1848	IPM 61	*Pectobacterium*	*atrosepticum*				Potato	Colorado, USA	G. Frank	P		A			P	Obtained	
K0570	A1853	M785	*Pectobacterium*	*atrosepticum*				Potato	Colorado, USA	G. Frank	P		A			P	Obtained	
K0571	A1847	IPM 60	*Pectobacterium*	*carotovorum*				Potato	Colorado, USA	G. Frank	P		A			P	NotObtained	
K0572	A1850	IPM 1260	*Pectobacterium*	*atrosepticum*				Potato	Colorado, USA	G. Frank	P		A			P	Obtained	
K0573	A1855	M2954	*Pectobacterium*	*atrosepticum*				Potato	Colorado, USA	G. Frank	P		A			P	Obtained	

K0574	A1852A	M784	*Pectobacterium*	*carotovorum*				Potato	Colorado, USA	G. Frank	A		A			WEIRD	NotObtained	P
K0575	A2312A	E11	*Dickeya*	*sp.*				Ornamental	Florida, USA	A.Chase	P		P			P	NotObtained	
K0576	A2312B	E11	*Dickeya*	*sp.*				Ornamental	Florida, USA	A.Chase	P		P			P	Obtained	
K0577	A1314	DR-93 purified	*Xanthomonas*	*maltophilia*				Cabbage seed	California, USA	W. Wiebe	A					A	NotObtained	
K0578	A5082	G-5	*Xanthomonas*	*campestris*		*campestris*		Cabbage	Georgia, USA	R. Gitaitis	P					P	Obtained	
K0579	A5083	G-6	*Xanthomonas*	*campestris*		*campestris*		Cabbage	Georgia, USA	R. Gitaitis	P					P	Obtained	
K0580	A5098	G-4	*Xanthomonas*	*campestris*		*campestris*		Cabbage	Georgia, USA	R. Gitaitis	P					P	Obtained	
K0581	A5374	E-6	*Xanthomonas*	*campestris*		*campestris*		Cabbage	Georgia, USA	R. Gitaitis	P					P	NotObtained	
K0582	A88	A88-3	*Xanthomonas*	*axonopodis*		*allii*		Onion	Hawaii, USA	A. Alvarez	P					P	NotObtained	
K0583	A94	A94-1B	*Xanthomonas*	*axonopodis*		*allii*		Onion	Hawaii, USA	A. Alvarez						P	Obtained	
K0584	A118	A118-2A	*Xanthomonas*	*axonopodis*		*allii*		Onion	Hawaii, USA	A. Alvarez	P					P	Obtained	
K0585	A206	A206-2A	*Xanthomonas*	*axonopodis*		*allii*		Onion	Hawaii, USA	A. Alvarez	P					P	Obtained	
K0586	A206	A206-5	*Xanthomonas*	*axonopodis*		*allii*		Onion	Hawaii, USA	A. Alvarez	P					P	Obtained	
K0587	A225	A225-5	*Xanthomonas*	*axonopodis*		*allii*		Onion	Hawaii, USA	A. Alvarez	P					P	Obtained	
K0588	A227	A227-1	*Xanthomonas*	*axonopodis*		*allii*		Allium cepa	Hawaii, USA	A. Alvarez						P	Obtained	
K0589	A229	A229	*Xanthomonas*	*axonopodis*		*allii*		Allium cepa	Hawaii, USA	A. Alvarez	A					P	Obtained	
K0590	A255	A255-4	*Xanthomonas*	*axonopodis*		*allii*		Onion	Hawaii, USA	A. Alvarez	P					P	Obtained	
K0591	A256	A256-3	*Xanthomonas*	*axonopodis*		*allii*		Onion	Hawaii, USA	A. Alvarez	P					P	Obtained	
K0592	A1763	HMB9	*Xanthomonas*	*axonopodis*		*manihotis*		Cassava	Africa	H. Maraite						P	Obtained	
K0593	A274	A274-7	*Xanthomonas*	*axonopodis*		*allii*		Onion	Hawaii, USA	A. Alvarez	P					P	Obtained	
K0594	A554	A554-3	*Xanthomonas*	*axonopodis*		*allii*		Onion	Hawaii, USA	A. Alvarez	P					P	Obtained	
K0595	A579	A579-7	*Xanthomonas*	*axonopodis*		*allii*		Onion	Hawaii, USA	A. Alvarez	P					P	Obtained	
K0596	A1108	QR-30, ATCC 11725	*Xanthomonas*	*axonopodis*		*begoniae*		Begonia	New York, USA	M. P. Starr						P	NotObtained	
K0597	A1141	QR-71	*Xanthomonas*	*axonopodis*		*vitians*		Lettuce	Hawaii, USA	J. Cho	P					P	Obtained	

K0598	A1281	DR-29a purified	*Xanthomonas*	*maltophilia*				Cabbage seed	California, USA	W. Wiebe	P					P	Obtained	
K0599	A1283	DR-34 purified	*Xanthomonas*	*maltophilia*				Cabbage seed	California, USA	W. Wiebe						P	Obtained	
K0600	A1284	DR-35 purified	*Xanthomonas*	*maltophilia*				Cabbage seed	California, USA	W. Wiebe	P					P	Obtained	
K0601	A1286	DR-42 purified	*Xanthomonas*	*maltophilia*				Cabbage seed	California, USA	W. Wiebe	P					P	Obtained	
K0602	A1287	DR-44 purified	*Xanthomonas*	*maltophilia*				Cabbage seed	California, USA	W. Wiebe	P					P	NotObtained	
K0603	A1288	DR-47 purified	*Xanthomonas*	*maltophilia*				Cabbage seed	California, USA	W. Wiebe	P					P	Obtained	
K0604	A1289	DR-46a purified	*Xanthomonas*	*maltophilia*				Cabbage seed	California, USA	W. Wiebe	P					P	Obtained	
K0605	A1291	DR-51 purified	*Xanthomonas*	*maltophilia*				Cabbage seed	California, USA	W. Wiebe	P					P	Obtained	
K0606	A1292	DR-52 purified	*Xanthomonas*	*maltophilia*				Cabbage seed	California, USA	W. Wiebe						P	Obtained	
K0607	A1293	DR-54 purified	*Xanthomonas*	*maltophilia*				Cabbage seed	California, USA	W. Wiebe	P					P	Obtained	
K0608	A1295	DR-57 purified	*Xanthomonas*	*maltophilia*				Cabbage seed	California, USA	W. Wiebe	P					P	Obtained	
K0609	A1305	DR-64d purified	*Xanthomonas*	*maltophilia*				Cabbage seed	California, USA	W. Wiebe	P					P	Obtained	
K0610	A1315	W-2	*Xanthomonas*	*maltophilia*				Cabbage seed	Hawaii, USA	A. Alvarez	P					A	NotObtained	
K0611	A1694	B-60	*Xanthomonas*	*euvesicatoria*				Tomato	California, USA	J. Watterson						P	NotObtained	
K0612	A1695	B-61	*Xanthomonas*	*euvesicatoria*				Tomato	California, USA	J. Watterson	P					A	NotObtained	
K0613	A1696	B-71	*Xanthomonas*	*euvesicatoria*				Tomato	California, USA	J. Watterson	P					P	Obtained	
K0614	A1697	B-79	*Xanthomonas*	*euvesicatoria*				Tomato	California, USA	J. Watterson						P	Obtained	
K0615	A1698	B-80	*Xanthomonas*	*euvesicatoria*				Tomato	California, USA	J. Watterson	P					P	Obtained	

K0616	A1699	B-82	*Xanthomonas*	*euvesicatoria*				Tomato	California, USA	J. Watterson						P	Obtained	
K0617	A1701	B-94	*Xanthomonas*	*euvesicatoria*				Tomato	California, USA	J. Watterson						P	Obtained	
K0618	A1702	B-111	*Xanthomonas*	*euvesicatoria*				Tomato	California, USA	J. Watterson						P	Obtained	
K0619	A1703	B-118	*Xanthomonas*	*euvesicatoria*				Tomato	California, USA	J. Watterson	P					A	NotObtained	
K0620	A1704	B-122	*Xanthomonas*	*euvesicatoria*				Tomato	California, USA	J. Watterson	P					A	NotObtained	
K0621	A1705	XV-1	*Xanthomonas*	*euvesicatoria*				Tomato	California, USA	J. Watterson	P					A	NotObtained	
K0622	A1706	B-62	*Xanthomonas*	*euvesicatoria*				Tomato	California, USA	J. Watterson						P	Obtained	
K0623	A1708	B-93	*Xanthomonas*	*euvesicatoria*				Tomato	California, USA	J. Watterson						P	Obtained	
K0624	A1709	B-108	*Xanthomonas*	*euvesicatoria*				Tomato	California, USA	J. Watterson						P	Obtained	
K0625	A1711	B-63	*Xanthomonas*	*euvesicatoria*				Tomato	California, USA	J. Watterson						P	Obtained	
K0626	A1713	B-78	*Xanthomonas*	*euvesicatoria*				Tomato	California, USA	J. Watterson						P	Obtained	
K0627	A1714	B-81	*Xanthomonas*	*euvesicatoria*				Tomato	California, USA	J. Watterson						P	Obtained	
K0628	A1715	B-92	*Xanthomonas*	*euvesicatoria*				Tomato	California, USA	J. Watterson						P	Obtained	
K0629	A1716	B-95	*Xanthomonas*	*euvesicatoria*				Tomato	California, USA	J. Watterson						P	Obtained	
K0630	A1718	B-106	*Xanthomonas*	*euvesicatoria*				Tomato	California, USA	J. Watterson						P	Obtained	
K0631	A1719	B-70	*Xanthomonas*	*hortorum*		*carotae*		Carrot	California, USA	J. Watterson						P	Obtained	
K0632	A1720	B-75	*Xanthomonas*	*hortorum*		*carotae*		Carrot	California, USA	J. Watterson						P	Obtained	
K0633	A1721	B-103	*Xanthomonas*	*hortorum*		*carotae*		Carrot	California, USA	J. Watterson						P	Obtained	

K0634	A1722	86-1	*Xanthomonas*	*campestris*		*raphani*		crucifer	Georgia, USA	R. Gitaitis						P	Obtained	
K0635	A1723	86-2	*Xanthomonas*	*campestris*		*raphani*		crucifer	Georgia, USA	R. Gitaitis						P	Obtained	
K0636	A1724	86-3	*Xanthomonas*	*campestris*		*raphani*		crucifer	Georgia, USA	R. Gitaitis	A					P	Obtained	
K0637	A1725	X.85-AY	*Xanthomonas*	*campestris*		*raphani*		crucifer	Georgia, USA	R. Gitaitis	P					P	Obtained	
K0638	A1728	X.85-F	*Xanthomonas*	*campestris*		*raphani*		crucifer	Georgia, USA	R. Gitaitis						P	Obtained	
K0639	A1730	XLS-2	*Xanthomonas*	*campestris*		*armoraciae*		crucifer	Louisiana, USA	L. Black						P	Obtained	
K0640	A1732	XLS-4	*Xanthomonas*	*campestris*		*armoraciae*		crucifer	Louisiana, USA	L. Black						P	Obtained	
K0641	A1757	XCV-1	*Xanthomonas*	*euvesicatoria*				Tomato	North Carolina, USA	M. Cubeta						P	Obtained	
K0642	A1767	HMB 286	*Xanthomonas*	*axonopodis*		*manihotis*		Cassava	France	H. Maraite						P	Obtained	
K0643	A1769	HMB 38	*Xanthomonas*	*axonopodis*		*manihotis*		Cassava	France	H. Maraite	P					P	NotObtained	
K0644	A1772	HMB 296	*Xanthomonas*	*axonopodis*		*manihotis*		Cassava	France	H. Maraite	P					P	NotObtained	
K0645	A1773	XCV2	*Xanthomonas*	*euvesicatoria*				Tomato	North Carolina, USA	M. Cubeta						P	Obtained	
K0646	A1788	Kawai III KPL	*Xanthomonas*	*euvesicatoria*				Pepper	Hawaii, USA	B. S. Kim						P	Obtained	
K0647	A1804	XCO	*Xanthomonas*	*oryzae*		*oryzae*		Rice	Hawaii, USA	Mike G.						P	Obtained	
K0648	A1806	X3	*Xanthomonas*	*axonopodis*		*poinsettiicola*		Poinsettia	Florida, USA	A. Chase	P					P	NotObtained	
K0649	A1811	X32	*Xanthomonas*	*species*		*urticae*		Nettle	Florida, USA	A. Chase	P					P	NotObtained	
K0650	A1813	X42	*Xanthomonas*	*axonopodis*		*vitians*		Lettuce	Florida, USA	A. Chase						P	NotObtained	
K0651	A1814	X45	*Xanthomonas*	*axonopodis*		*begoniae*		Begonia	Florida, USA	A. Chase						P	NotObtained	
K0652	A1816	X6	*Xanthomonas*	*euvesicatoria*				Pepper	Florida, USA	A. Chase	P					P	NotObtained	

K0653	A1818	ARCO 2	*Xanthomonas*	*hortorum*		*carotae*		Carrot	California, USA	ARCO Seed Comp.						P	Obtained	
K0654	A1819	ARCO 3	*Xanthomonas*	*hortorum*		*carotae*		Carrot	California, USA	ARCO Seed Comp.						P	Obtained	
K0655	A1874	417	*Xanthomonas*	*campestris*		*armoraciae*		Cabbage	Florida, USA	A. Chase						P	Obtained	
K0656	A3192	G44-Ser1	*Xanthomonas*	*albilineans*				Sugarcane	Guadeloupe	Baudin	P					P	NotObtained	
K0657	A3193	HV5-Ser2	*Xanthomonas*	*albilineans*				Sugarcane	Burkina Faso	Baudin	P					P	Obtained	
K0658	A3195	G7-Ser3	*Xanthomonas*	*albilineans*				Sugarcane	Guadeloupe	Baudin	P					P	Obtained	
K0659	A3302	GPE2	*Xanthomonas*	*albilineans*				Sugarcane	France	P. Rott	P					P	Obtained	
K0660	A3750	35F2	*Xanthomonas*	*fragariae*				Strawberry	California, USA	D.C. Opgenorth	P					P	NotObtained	
K0661	A3752	30F2	*Xanthomonas*	*fragariae*				Strawberry	California, USA	D.C. Opgenorth	A					P	NotObtained	
K0662	A3813	G14	*Xanthomonas*	*oryzae*		*oryzae*		Rice	India	S. Gnanamanickam	P					P	Obtained	
K0663	A1887	A 135-1	*Xanthomonas*	*euvesicatoria*				Tomato	Hawaii, USA	A. Alvarez						P	NotObtained	
K0664	A1891	G 715	*Xanthomonas*	*axonopodis*		*panax*		Panax	Hawaii, USA	A. Alvarez	P					P	Obtained	
K0665	A1892	A674-2B	*Xanthomonas*	*axonopodis*		*vitians*		Lettuce	Hawaii, USA	A. Alvarez	P					P	Obtained	
K0666	A1917	62-8	*Xanthomonas*	*euvesicatoria*				tomato/pepper	Florida, USA	J. B. Jones						P	Obtained	
K0667	A1918	65-2a	*Xanthomonas*	*euvesicatoria*				tomato/pepper	Florida, USA	J. B. Jones						P	Obtained	
K0668	A1920	68-1	*Xanthomonas*	*euvesicatoria*				tomato/pepper	Florida, USA	J. B. Jones						P	Obtained	
K0669	A1922	71-21	*Xanthomonas*	*euvesicatoria*				tomato/pepper	Florida, USA	J. B. Jones						P	Obtained	
K0670	A1973	X162	*Xanthomonas*	*axonopodis*		*syngonii*		Syngonium	Florida, USA	A. Chase						P	Obtained	
K0671	A1974	X163	*Xanthomonas*	*axonopodis*		*syngonii*		Syngonium	Florida, USA	A. Chase						P	Obtained	
K0672	A1984	X181	*Xanthomonas*	*axonopodis*		*syngonii*		Syngonium	Florida, USA	A. Chase						P	Obtained	

K0673	A3206	PD 427	*Ralstonia*	*solanacearum*			Race 3, Biovar 2	Potato	Netherlands	J. D. Janse				P	NotObtained	P	Obtained	
K0674	A3784	MT1	*Ralstonia*	*solanacearum*			Race 2	Abaca	Philippines	M. Natural				P	Obtained	P	Obtained	
K0675	A4620	PS-20	*Ralstonia*	*solanacearum*			unknown Race	Geranium	Guatemala	D. Thomas				P	Obtained	P	Obtained	
K0676	A4621	PS-22	*Ralstonia*	*solanacearum*			unknown Race	Geranium	Guatemala	D. Thomas	P			P	Obtained	P	Obtained	
K0677	A4622	PS-31	*Ralstonia*	*solanacearum*			unknown Race	Geranium	Guatemala	D. Thomas	A			P	Obtained	P	Obtained	
K0678	A4643	Sample6	*Ralstonia*	*solanacearum*			Blood Disease	Banana	Indonesia	J. Bartlett				P	Obtained	P	Obtained	
K0679	A4646	4B-1	*Ralstonia*	*solanacearum*			Race 4	Ginger	Hawaii, USA	R. Lipp				P	Obtained	P	Obtained	
K0680	A4647	4B-3	*Ralstonia*	*solanacearum*			Race 4	Ginger	Hawaii, USA	R. Lipp				P	Obtained	P	Obtained	
K0681	A4648	4B-4	*Ralstonia*	*solanacearum*			Race 4	Ginger	Hawaii, USA	R. Lipp	P			A	N/A	P	Obtained	
K0682	A4649	4B-5	*Ralstonia*	*solanacearum*			Race 4	Ginger	Hawaii, USA	R. Lipp	P			A	N/A	A	NotObtained	
K0683	A4650	4B-6	*Ralstonia*	*solanacearum*			Race 4	Ginger	Hawaii, USA	R. Lipp				P	Obtained	P	Obtained	
K0684	A4651	4B-8	*Ralstonia*	*solanacearum*			Race 4	Ginger	Hawaii, USA	R. Lipp	P			A	N/A	P	Obtained	
K0685	A4652	4B-9	*Ralstonia*	*solanacearum*			Race 4	Ginger	Hawaii, USA	R. Lipp	P			A	N/A	P	Obtained	
K0686	A4653	4B-10	*Ralstonia*	*solanacearum*			Race 4	Ginger	Hawaii, USA	R. Lipp				P	NotObtained	P	Obtained	
K0687	A4654	C 443-3	*Ralstonia*	*solanacearum*			Race 4	Ginger	Hawaii, USA	R. Lipp	P			A	N/A	A	NotObtained	
K0688	A4734	AA-4	*Ralstonia*	*solanacearum*			Race 4	Ginger	Hawaii, USA	B. Bushe				P	Obtained	P	Obtained	
K0689	A4735	AA-6	*Ralstonia*	*solanacearum*			Race 4	Ginger	Hawaii, USA	B. Bushe				P	NotObtained	P	Obtained	
K0690	A4736		*Ralstonia*	*solanacearum*			Race 1	Tomato	Hawaii, USA	J. Uchida				P	NotObtained	P	Obtained	
K0691	A5181	DP-6	*Ralstonia*	*solanacearum*			Race 4	Ginger	Hawaii, USA	P. Hepperly				P	Obtained	P	Obtained	
K0692	A5182	DP-9	*Ralstonia*	*solanacearum*			Race 4	Ginger	Hawaii, USA	P. Hepperly				P	Obtained	P	Obtained	
K0693	A5183	DP-11	*Ralstonia*	*solanacearum*			Race 4	Ginger	Hawaii, USA	P. Hepperly				P	Obtained	P	Obtained	
K0694	A5184	DP-13	*Ralstonia*	*solanacearum*			Race 4	Ginger	Hawaii, USA	P. Hepperly				P	Obtained	P	Obtained	
K0695	A5185	DP-22	*Ralstonia*	*solanacearum*			Race 4	Ginger	Hawaii, USA	P. Hepperly	P			A	N/A	P	Obtained	
K0696	A5189	DSK-5	*Ralstonia*	*solanacearum*			Race 4	Ginger	Hawaii, USA	P. Hepperly				P	Obtained	P	Obtained	
K0697	A5190	PO-1	*Ralstonia*	*solanacearum*			Race 4	Ginger	Hawaii, USA	P. Hepperly				P	Obtained	P	Obtained	
K0698	A5191	BI-9	*Ralstonia*	*solanacearum*			Race 4	Ginger	Hawaii, USA	P. Hepperly				P	Obtained	P	Obtained	

K0699	A5193	DSK-1	*Ralstonia*	*solanacearum*			Race 4	Ginger	Hawaii, USA	P. Hepperly				P	Obtained	P	Obtained	
K0700	A5194	PO-25	*Ralstonia*	*solanacearum*			Race 4	Ginger	Hawaii, USA	P. Hepperly				P	Obtained	P	Obtained	
K0701	A5195	DSK-2	*Ralstonia*	*solanacearum*			Race 4	Ginger	Hawaii, USA	P. Hepperly				P	NotObtained	P	Obtained	
K0702	A5196	DSK-4	*Ralstonia*	*solanacearum*			Race 4	Ginger	Hawaii, USA	P. Hepperly				P	Obtained	P	Obtained	
K0703	A5197	PO-6	*Ralstonia*	*solanacearum*			Race 4	Ginger	Hawaii, USA	P. Hepperly				P	Obtained	P	Obtained	
K0704	A5198	BI-17	*Ralstonia*	*solanacearum*			Race 4	Ginger	Hawaii, USA	P. Hepperly				P	NotObtained	P	Obtained	
K0705	A5199	BI-13	*Ralstonia*	*solanacearum*			Race 4	Ginger	Hawaii, USA	P. Hepperly				P	Obtained	P	Obtained	
K0708	A5285	UW 40 (K248)	*Ralstonia*	*solanacearum*			Race 2	Banana (Chato)	Honduras	I.W. Buddenhagen				P	Obtained	P	Obtained	
K0709	A5294	91/04	*Ralstonia*	*solanacearum*			Race 2	Banana (Cavendish)	Jamaica	M. Young	P			P	NotObtained	P	Obtained	
K0710	A5295-b	91(0)/04	*Ralstonia*	*solanacearum*			Race 2	Banana (Cavendish)	Jamaica	M. Young	P			A	N/A	A	NotObtained	
K0711	A5295-c	91(0)/04	*Ralstonia*	*solanacearum*			Race 2	Banana (Cavendish)	Jamaica	M. Young				P	NotObtained	P	Obtained	
K0712	A5295-e	91(0)/04	*Ralstonia*	*solanacearum*			Race 2	Banana (Cavendish)	Jamaica	M. Young	P			A	N/A	A	NotObtained	
K0713	A5295-f	91(0)/04	*Ralstonia*	*solanacearum*			Race 2	Banana (Cavendish)	Jamaica	M. Young	P			A	N/A	A	NotObtained	
K0714	A5364	BDB-Like B strain	*Ralstonia*	*solanacearum*			Blood Disease	Banana	Indonesia	I.W. Buddenhagen				P	Obtained	P	Obtained	
K0715	A5363	BDBiovar 1	*Ralstonia*	*solanacearum*			Blood Disease	Banana	Indonesia	I.W. Buddenhagen				SMEAR	Obtained	P	Obtained	
K0716	A5470	PO-2	*Ralstonia*	*solanacearum*			Race 4	Ginger	Hawaii, USA	P. Hepperly				P	Obtained	P	Obtained	
K0717	A5471	PO-3	*Ralstonia*	*solanacearum*			Race 4	Ginger	Hawaii, USA	P. Hepperly				P	Obtained	P	Obtained	
K0718	A5472	PO-4	*Ralstonia*	*solanacearum*			Race 4	Ginger	Hawaii, USA	P. Hepperly				P	Obtained	P	Obtained	
K0719	A5473	PO-5	*Ralstonia*	*solanacearum*			Race 4	Ginger	Hawaii, USA	P. Hepperly				P	Obtained	P	Obtained	
K0720	A5474	PO-8	*Ralstonia*	*solanacearum*			Race 4	Ginger	Hawaii, USA	P. Hepperly				P	Obtained	P	Obtained	
K0721	A5475	PO-9	*Ralstonia*	*solanacearum*			Race 4	Ginger	Hawaii, USA	P. Hepperly				P	Obtained	P	Obtained	
K0722	A5476	PO-10	*Ralstonia*	*solanacearum*			Race 4	Ginger	Hawaii, USA	P. Hepperly				P	Obtained	P	Obtained	
K0723	A5477	PO-11	*Ralstonia*	*solanacearum*			Race 4	Ginger	Hawaii, USA	P. Hepperly				P	Obtained	P	Obtained	
K0724	A5478	PO-12	*Ralstonia*	*solanacearum*			Race 4	Ginger	Hawaii, USA	P. Hepperly				P	Obtained	P	Obtained	

K0725	A5479	PO-13	*Ralstonia*	*solanacearum*			Race 4	Ginger	Hawaii, USA	P. Hepperly				P	Obtained	P	Obtained	
K0726	A5480	PO-14	*Ralstonia*	*solanacearum*			Race 4	Ginger	Hawaii, USA	P. Hepperly				P	Obtained	P	Obtained	
K0727	A5481	PO-15	*Ralstonia*	*solanacearum*			Race 4	Ginger	Hawaii, USA	P. Hepperly				P	Obtained	P	Obtained	
K0728	A5482	PO-16	*Ralstonia*	*solanacearum*			Race 4	Ginger	Hawaii, USA	P. Hepperly				P	Obtained	P	Obtained	
K0729	A5483	PO-18	*Ralstonia*	*solanacearum*			Race 4	Ginger	Hawaii, USA	P. Hepperly				P	NotObtained	P	Obtained	
K0730	A5484	PO-19	*Ralstonia*	*solanacearum*			Race 4	Ginger	Hawaii, USA	P. Hepperly	P			A	N/A	P	Obtained	
K0731	A5485	PO-20	*Ralstonia*	*solanacearum*			Race 4	Ginger	Hawaii, USA	P. Hepperly				P	Obtained	P	Obtained	
K0732	A5486	PO-21	*Ralstonia*	*solanacearum*			Race 4	Ginger	Hawaii, USA	P. Hepperly				P	Obtained	P	Obtained	
K0734	A3787	340	*Ralstonia*	*solanacearum*			Race 2	Heliconia	Hawaii, USA	B. Bushe				P	Obtained	P	Obtained	
K0735	A4087	399CP	*Ralstonia*	*solanacearum*			Race 4	Ginger	Hawaii, USA	B. Bushe				P	Obtained	P	Obtained	
K0736	A4613	PS-11	*Ralstonia*	*solanacearum*			unknow n Race	Geranium	Guatemala	D. Thomas				P	Obtained	P	Obtained	
K0737	A4623A	PS-13 CAN-Owen 9	*Ralstonia*	*solanacearum*			Blood Disease	Banana	Sulawesi, Indonesia	I.W. Buddenhagen				P	Obtained	P	Obtained	
K0739	A5155	QR88	*Ralstonia*	*solanacearum*			Race 1	Tomato	North Carolina, USA	A. Kelman	P			A	N/A	P	Obtained	
K0740	A5156	QR87	*Ralstonia*	*solanacearum*			Race 1	Tomato	North Carolina, USA	A. Kelman				P	Obtained	P	Obtained	
K0741	A5281	27(2)	*Ralstonia*	*solanacearum*			Race 2	Heliconia	Jamaica	Marina Young				P	Obtained	P	Obtained	
K0742	A5282	25(3)	*Ralstonia*	*solanacearum*			Race 2	Banana (Cavendish)	Jamaica	Marina Young				P	Obtained	P	Obtained	
K0743	A5283	19(3)	*Ralstonia*	*solanacearum*			Race 2	Banana (Cavendish)	Jamaica	Marina Young				P	Obtained	P	Obtained	
K0744	A5284	24(1b)	*Ralstonia*	*solanacearum*			Race 2	Banana (Cavendish)	Jamaica	Marina Young				P	Obtained	P	Obtained	
K0745	A5287	UW257	*Ralstonia*	*solanacearum*	-	-	Race 3 Biovar 2	Potato	Chicoa, Costa Rica	Caitilyn Allen				P	Obtained	P	Obtained	
K0746	A4680	BCA	*Ralstonia*	*solanacearum*			Race 4	Ginger (Kahili)	Hawaii, USA	D. Gardner	A			A	N/A	A	NotObtained	
K0747	A5487	2	*Ralstonia*	*solanacearum*			Blood Disease	Banana-goroho	Sulawesi, Indonesia	I.W. Buddenhagen				P	Obtained	P	Obtained	
K0748	A5488	6	*Ralstonia*	*solanacearum*			Blood	Banana raja	Sulawesi,	I.W.				P	Obtained	P	Obtained	

							Disease	pseudostem	Indonesia	Buddenhagen								
K0749	A5489	8/2	*Ralstonia*	*solanacearum*			Blood Disease	Banana kapok	Indonesia	I.W. Buddenhagen	P			A	N/A	A	NotObtained	
K0750	A5490	B	*Ralstonia*	*solanacearum*			Blood Disease	Banana kapok	Java, Indonesia	I.W. Buddenhagen				P	Obtained	P	Obtained	
K0751	A5491	EBiovar 2	*Ralstonia*	*solanacearum*			Race 1	Eggplant	India	I.W. Buddenhagen	P			P	Obtained	A	NotObtained	
K0752	A5492	IBIA-2	*Ralstonia*	*solanacearum*			Race 1	Eggplant	India	I.W. Buddenhagen	P			A	N/A	P	Obtained	
K0753	A5493	IBIA-4B	*Ralstonia*	*solanacearum*			Race 4	Ginger	India	I.W. Buddenhagen	P			A	N/A	A	NotObtained	
K0754	A5494	IBIA-7	*Ralstonia*	*solanacearum*			Race 1	Tomato	India	I.W. Buddenhagen				A	N/A	P	Obtained	
K0755	A5495	IBIA-8	*Ralstonia*	*solanacearum*			Race 1	Chili	India	I.W. Buddenhagen				A	N/A	A	NotObtained	
K0756	A5496	IBIA-9	*Ralstonia*	*solanacearum*			Race 1	Eggplant	India	I.W. Buddenhagen				A	N/A	A	NotObtained	
K0757	A5497	C1	*Ralstonia*	*solanacearum*			Race 1	Chilli	India	I.W. Buddenhagen				P	Obtained	P	Obtained	
K0758	A5498	C2	*Ralstonia*	*solanacearum*			Race 1	Chilli	India	I.W. Buddenhagen				P	Obtained	P	Obtained	
K0759	A5499	10	*Ralstonia*	*solanacearum*			Blood Disease	Banana	Slayer Island, Indonesia	I.W. Buddenhagen				P	Obtained	P	Obtained	
K0760	A5500	11	*Ralstonia*	*solanacearum*			Blood Disease	Banana kapok	East Java, Indonesia	I.W. Buddenhagen				P	Obtained	P	Obtained	
K0761	A4614	PS-12	*Ralstonia*	*solanacearum*			unknown Race	Geranium	Guatemala	D. Thomas				P	Obtained	P	Obtained	
K0762	A4615	PS-13	*Ralstonia*	*solanacearum*			unknown Race	Geranium	Guatemala	D. Thomas				P	Obtained	P	Obtained	
K0763	A4616	PS-14	*Ralstonia*	*solanacearum*			unknown Race	Geranium	Guatemala	D. Thomas				P	Obtained	P	Obtained	
K0764	A4617	PS-15	*Ralstonia*	*solanacearum*			unknown Race	Geranium	Guatemala	D. Thomas				P	Obtained	P	Obtained	
K0765	A4618	PS-17	*Ralstonia*	*solanacearum*			unknown Race	Geranium	Guatemala	D. Thomas				P	Obtained	P	Obtained	

K0766	A5527	PO-22	*Ralstonia*	*solanacearum*			Race 4	Ginger	Hawaii, USA	P. Hepperly				P	NotObtained	P	Obtained	
K0767	A3343	BN-1	*Ralstonia*	*solanacearum*			Race 1	Tomato	Hawaii, USA	A. Alvarez				P	Obtained	P	Obtained	
K0768	A3588	TM2 90.5	*Ralstonia*	*solanacearum*			Race 1	Tomato	China	L. Y. He				P	Obtained	P	Obtained	
K0769	A5528	UW19	*Ralstonia*	*solanacearum*			Race 3, Biovar 2	Potato	Columbia	C. Allen				P	NotObtained	P	Obtained	
K0770	A5529	UW23	*Ralstonia*	*solanacearum*			Race 3, Biovar 2	Potato	Israel	C. Allen				P	Obtained	P	Obtained	
K0771	A5530	UW37	*Ralstonia*	*solanacearum*			Race 3, Biovar 2	Potato	Colombia	C. Allen				P	Obtained	P	Obtained	
K0772	A5531	UW72	*Ralstonia*	*solanacearum*			Race 3, Biovar 2	Potato	Greece	C. Allen				P	Obtained	P	Obtained	
K0773	A5532	UW73	*Ralstonia*	*solanacearum*			Race 3, Biovar 2	Potato	Ceylon	C. Allen				P	Obtained	P	Obtained	
K0774	A5533	UW81	*Ralstonia*	*solanacearum*			Race 3, Biovar 2	Potato	Colombia	C. Allen				P	Obtained	P	Obtained	
K0775	A5534	UW120	*Ralstonia*	*solanacearum*			Race 3, Biovar 2	Potato	Costa Rica	C. Allen	A			A	N/A	A	NotObtained	
K0776	A5535	UW145	*Ralstonia*	*solanacearum*			Race 3, Biovar 2	Potato	Australia	C. Allen				P	Obtained	P	Obtained	
K0777	A5536	UW150	*Ralstonia*	*solanacearum*			Race 3, Biovar 2	Potato	Australia	C. Allen				P	Obtained	P	Obtained	
K0778	A5537	UW220a	*Ralstonia*	*solanacearum*			Race 3, Biovar 2	Potato	India	C. Allen				P	Obtained	P	Obtained	
K0779	A5538	UW221	*Ralstonia*	*solanacearum*			Race 3, Biovar 2	Solanum nigrum	Kenya	C. Allen				P	Obtained	P	NotObtained	
K0780	A5539	UW224	*Ralstonia*	*solanacearum*			Race 3, Biovar 2	Potato	Kenya	C. Allen				P	Obtained	P	Obtained	
K0781	A5540	UW260	*Ralstonia*	*solanacearum*			Race 3, Biovar 2	Potato	Cajamarca, Peru	C. Allen				P	N/A	P	Obtained	
K0782	A5541	UW276	*Ralstonia*	*solanacearum*			Race 3, Biovar 2	Potato	Mexico	C. Allen				P	N/A	P	Obtained	
K0783	A5542	UW348	*Ralstonia*	*solanacearum*			Race 3, Biovar 2	Potato	Brazil	C. Allen				P	N/A	P	NotObtained	
K0784	A5543	UW365	*Ralstonia*	*solanacearum*			Race 3, Biovar 2	Potato	China	C. Allen				P	N/A	P	Obtained	

K0785	A5544	UW408	*Ralstonia*	*solanacearum*			Race 3, Biovar 2	Potato	Australia	C. Allen				P	N/A	P	Obtained	
K0786	A5545	UW433	*Ralstonia*	*solanacearum*			Abberant Biovar 2	Tomato	Australia	C. Allen				P	N/A	P	Obtained	
K0787	A5546	UW437	*Ralstonia*	*solanacearum*			Race 3, Biovar 2	Tomato	Australia	C. Allen				P	N/A	P	Obtained	
K0788	A5547	UW441	*Ralstonia*	*solanacearum*			Race 3, Biovar 2	Potato	Australia	C. Allen				P	N/A	P	Obtained	
K0789	A5548	UW448	*Ralstonia*	*solanacearum*			Race 3, Biovar 2	Potato	Burundi	C. Allen				P	N/A	P	Obtained	
K0790	A5549	UW453	*Ralstonia*	*solanacearum*			Race 3, Biovar 2	Potato	Nigeria	C. Allen				P	N/A	P	Obtained	
K0791	A5550	UW473	*Ralstonia*	*solanacearum*			Race 3, Biovar 2	Potato	Peru	C. Allen				P	N/A	P	Obtained	
K0792	A5551	UW489	*Ralstonia*	*solanacearum*			Race 3, Biovar 2	Potato	Chile	C. Allen				A	N/A	A	NotObtained	
K0793	A5552	UW491	*Ralstonia*	*solanacearum*			Race 3, Biovar 2	Potato	Colombia	C. Allen				P	N/A	P	Obtained	
K0794	A5553	UW500	*Ralstonia*	*solanacearum*			Race 3, Biovar 2	Potato	Java, Indonesia	C. Allen				P	N/A	P	Obtained	
K0795	A5554	UW523	*Ralstonia*	*solanacearum*			Race 3, Biovar 2	Tomato	France	C. Allen				P	N/A	P	Obtained	
K0796	A5555	UW556	*Ralstonia*	*solanacearum*			Race 3, Biovar 2	Potato	Guatemala	C. Allen				P	N/A	P	Obtained	
K0797	A5556	UW560	*Ralstonia*	*solanacearum*			Race 3, Biovar 2	Potato	Guatemala	C. Allen				P	N/A	P	Obtained	
K0798	A5557	UW596	*Ralstonia*	*solanacearum*			Race 3, Biovar 2	Potato	Guatemala	C. Allen				P	N/A	P	Obtained	
K0799	A5558	UW598	*Ralstonia*	*solanacearum*			Race 3, Biovar 2	Potato	Guatemala	C. Allen				P	N/A	P	NotObtained	
K0800	A5559	UW600	*Ralstonia*	*solanacearum*			Race 3, Biovar 2	Potato	Guatemala	C. Allen				P	N/A	P	Obtained	
K0801	A5560	UW604	*Ralstonia*	*solanacearum*			Race 3, Biovar 2	Potato	Guatemala	C. Allen				P	N/A	A	NotObtained	
K0802	A5561	UW610	*Ralstonia*	*solanacearum*			Race 3,	Tomato	Guatemala	C. Allen				P	N/A	A	NotObtained	

							Biovar 2											
K0803	A5562	UW612	*Ralstonia*	*solanacearum*			Race 3, Biovar 2	Tomato	Guatemala	C. Allen				P	N/A	P	Obtained	
K0804	A5286	UW80	*Ralstonia*	*solanacearum*			Race 3, Biovar 2	Solanum phureja	Colombia	C. Allen				P	N/A	P	Obtained	
K0805	A5288	UW344	*Ralstonia*	*solanacearum*			Race 3, Biovar 2	Potato	Brazil	C. Allen				P	N/A	P	Obtained	
K0806	A5291	UW552	*Ralstonia*	*solanacearum*			Race 3, Biovar 2	Geranium	Guatemala	C. Allen				P	N/A	P	Obtained	
K0807	A5563	BDB-2	*Ralstonia*	*solanacearum*			Blood Disease	Banana	Sulawesi, Indonesia	I.W. Buddenhagen				P	N/A	P	Obtained	
K0808	A5564	IBUD Indonesia (2)	*Ralstonia*	*solanacearum*			Blood Disease	Banana	Sulawesi, Indonesia	I.W. Buddenhagen				P	N/A	P	Obtained	
K0809	A5565	IBUD Indonesia (10B)	*Ralstonia*	*solanacearum*			Blood Disease	Banana	Sulawesi, Indonesia	I.W. Buddenhagen				P	N/A	P	Obtained	
K0810	A342	A342	*Xanthomonas*	*campestris*		*campestris*		Broccoli	Hawaii, USA	A. Alvarez						P	Obtained	
K0811	A1809	X29	*Xanthomonas*	*axonopodis*		*dieffenbachiae*		aroid	Florida, USA	A. Chase						P	Obtained	
K0812	A1962	X1	*Xanthomonas*	*axonopodis*		*dieffenbachiae*		aroid	Florida, USA	A. Chase						P	Obtained	
K0813	A1964	X13	*Xanthomonas*	*axonopodis*		*dieffenbachiae*		aroid	Florida, USA	A. Chase						P	Obtained	
K0814	A1965	X14	*Xanthomonas*	*axonopodis*		*dieffenbachiae*		aroid	Florida, USA	A. Chase						P	Obtained	
K0815	A1976	X169	*Xanthomonas*	*axonopodis*		*dieffenbachiae*		aroid	Florida, USA	A. Chase						P	Obtained	
K0816	A1978	X171	*Xanthomonas*	*axonopodis*		*dieffenbachiae*		aroid	Florida, USA	A. Chase						P	Obtained	
K0817	A1986	X183	*Xanthomonas*	*axonopodis*		*dieffenbachiae*		aroid	Florida, USA	A. Chase						P	Obtained	
K0818	A1989	X186	*Xanthomonas*	*axonopodis*		*dieffenbachiae*		aroid	Florida, USA	A. Chase						P	Obtained	
K0819	A2046	1591	*Xanthomonas*	*axonopodis*		*dieffenbachiae*		aroid	Australia	A. C. Hayward						P	Obtained	

K0820	A2655	J7	*Xanthomonas*	*axonopodis*		*dieffenbachiae*		Dieffenbachia	Jamaica	A. Alvarez						P	Obtained	
K0821	A2677	C-669-3a	*Xanthomonas*	*axonopodis*		*dieffenbachiae*		aroid	Hawaii, USA	R. Lipp	P					A	NotObtained	
K0822	A2679	C-750-3a	*Xanthomonas*	*axonopodis*		*dieffenbachiae*		aroid	Hawaii, USA	R. Lipp						P	Obtained	
K0823	A2681	C-895-1a	*Xanthomonas*	*axonopodis*		*dieffenbachiae*		aroid	Hawaii, USA	R. Lipp						P	Obtained	
K0824	A2707	C-959-1A	*Xanthomonas*	*axonopodis*		*dieffenbachiae*		aroid	Hawaii, USA	R. Lipp						P	Obtained	
K0825	A3115	G3-27	*Xanthomonas*	*campestris*		*campestris*		Cabbage	Hawaii, USA	A. Alvarez						P	Obtained	
K0826	A4692	THC-3	*Xanthomonas*	*campestris*		*campestris*		Cabbage	Themi, Nepal	D. Shakya						P	Obtained	
K0827	A4693	GRC-3	*Xanthomonas*	*campestris*		*campestris*		Cabbage	Themi, Nepal	D. Shakya						P	Obtained	
K0828	A4722	D-70	*Xanthomonas*	*axonopodis*		*dieffenbachiae*		Anthurium	Hawaii, USA	M. Aragaki						P	Obtained	
K0829	A4998	M2-8	*Xanthomonas*	*campestris*		*campestris*		Cabbage	Hawaii, USA	K. Trotter						P	Obtained	
K0830	A4999	PDDCC 4805b	*Xanthomonas*	*campestris*		*aberrans*		Cabbage	New Zealand	J. Young						P	Obtained	
K0831	A5000	PDDCC 4808b	*Xanthomonas*	*campestris*		*campestris*		Cabbage	New Zealand	J. Watterson						P	Obtained	
K0832	A5001	PDDCC 83-21	*Xanthomonas*	*campestris*		*armoraciae*		Cabbage	California, USA	R. Campbell						P	Obtained	
K0833	A5002	PDDCC 13-81	*Xanthomonas*	*campestris*		*armoraciae*		Cabbage	New Zealand	R. Gitaitis						P	Obtained	
K0834	A5003	PDDCC 574-65	*Xanthomonas*	*campestris*		*incanae*		Methiola incana	New Zealand	J. Young						P	Obtained	
K0835	A5004	QR27b	*Xanthomonas*	*campestris*		*aberrans*		Cabbage	India	M. P. Starr						P	Obtained	
K0836	A5005	PDDCC 1404-65	*Xanthomonas*	*campestris*		*raphani*		Radish	New Zealand	M. P. Starr						P	Obtained	
K0837	A5006	PDDCC 13a	*Xanthomonas*	*campestris*		*campestris*		Cabbage	New Zealand	J. Young						P	Obtained	
K0838	A5007	PDDCC 1405-76	*Xanthomonas*	*campestris*		*aberrans*		crucifer	New Zealand	J. Young						P	Obtained	

K0839	A5008	PDDCC 1404a	*Xanthomonas*	*campestris*		*raphani*		Radish	New Zealand	J. Young						P	Obtained	
K0840	A5009	808a	*Xanthomonas*	*campestris*		*armoraciae*		crucifer	California, USA	J. Watterson						P	Obtained	
K0841	A5102	CAM 075	*Xanthomonas*	*campestris*		*campestris*		crucifer seed	Idaho, USA	A. Poblawsky						P	Obtained	
K0842	A5103	CAM 076	*Xanthomonas*	*campestris*		*campestris*		crucifer seed	Idaho, USA	A. Poblawsky						P	Obtained	
K0843	A5127	A1061	*Xanthomonas*	*campestris*		*campestris*		Swine cress	Hawaii, USA	J. Cho						P	Obtained	
K0844	A5586	D-31	*Xanthomonas*	*axonopodis*		*dieffenbachiae*		Anthurium	Hawaii, USA	R. Lipp						P	Obtained	
K0845	A5587	D-36-3	*Xanthomonas*	*axonopodis*		*dieffenbachiae*		Syngonium	Hawaii, USA	R. Lipp						P	Obtained	
K0846	A5588	D-61-1	*Xanthomonas*	*axonopodis*		*dieffenbachiae*		Anthurium	Hawaii, USA	R. Lipp						P	Obtained	
K0847	A5589	D-74-3	*Xanthomonas*	*axonopodis*		*dieffenbachiae*		Dieffenbachia	Hawaii, USA	R. Lipp						P	Obtained	
K0848	A5590	D-89	*Xanthomonas*	*axonopodis*		*dieffenbachiae*		Anthurium	Hawaii, USA	R. Lipp						P	Obtained	
K0849	A5591	D-97-3	*Xanthomonas*	*axonopodis*		*dieffenbachiae*		Anthurium	Hawaii, USA	R. Lipp						P	Obtained	
K0850	A5592	D-109	*Xanthomonas*	*axonopodis*		*dieffenbachiae*		Anthurium	Hawaii, USA	R. Lipp	P					A	NotObtained	
K0851	A5593	D-110	*Xanthomonas*	*axonopodis*		*dieffenbachiae*		Anthurium	Hawaii, USA	R. Lipp						P	Obtained	
K0852	A5594	D-112	*Xanthomonas*	*axonopodis*		*dieffenbachiae*		Anthurium	Hawaii, USA	R. Lipp						P	Obtained	
K0853	A5595	D-114	*Xanthomonas*	*axonopodis*		*dieffenbachiae*		Anthurium	Hawaii, USA	R. Lipp						P	Obtained	
K0854	A5596	D-115	*Xanthomonas*	*axonopodis*		*dieffenbachiae*		Anthurium	Hawaii, USA	R. Lipp						P	Obtained	
K0855	A5597	D-116	*Xanthomonas*	*axonopodis*		*dieffenbachiae*		Anthurium	Hawaii, USA	R. Lipp						P	Obtained	
K0856	A5598	D-117	*Xanthomonas*	*axonopodis*		*dieffenbachiae*		Anthurium	Hawaii, USA	R. Lipp						P	Obtained	
K0857	A5599	D-118	*Xanthomonas*	*axonopodis*		*dieffenbachiae*		Anthurium	Hawaii, USA	R. Lipp						P	Obtained	

K0858	A5600	D-188	*Xanthomonas*	*axonopodis*		*dieffenbachiae*		Colocasia	Hawaii, USA	R. Lipp						P	Obtained	
K0860	A5602	Field XCD	*Xanthomonas*	*axonopodis*		*dieffenbachiae*		Anthurium	Hawaii, USA	A. Alvarez						P	Obtained	
K0861	A5603	LUX	*Xanthomonas*	*axonopodis*		*dieffenbachiae*		Anthurium	Hawaii, USA	A. Alvarez						P	Obtained	
K0862	A5604	CC99	*Xanthomonas*	*campestris*		*campestris*		Cabbage	Hawaii, USA	A. Alvarez						P	Obtained	
K0863	A1812	X38	*Xanthomonas*	*hortorum*		*pelargonii*		Geranium	Florida, USA	A. Chase						P	Obtained	
K0864	A1924	72-7	*Xanthomonas*	*euvesicatoria*				Pepper	Florida, USA	J. B. Jones						P	NotObtained	
K0865		7493	*Xanthomonas*	*axonopodis*		*citri*			New Zealand	J. Young						P	Obtained	
K0866		3103	*Xanthomonas*	*vasicola*					New Zealand	J. Young						P	Obtained	
K0867		8037	*Xanthomonas*	*vesicatoria*					New Zealand	J. Young						P	Obtained	
K0868		16690	*Xanthomonas*	*perforans*					New Zealand	J. Young						P	Obtained	
K0869		10022	*Xanthomonas*	*axonopodis*		*citri*			New Zealand	J. Young						P	NotObtained	
K0870		453	*Xanthomonas*	*hortorum*		*hederae*			New Zealand	J. Young						P	Obtained	
K0871		2299	*Xanthomonas*	*cucurbitae*					New Zealand	J. Young						P	Obtained	
K0872		659	*Xanthomonas*	*fragariae*					New Zealand	J. Young						P	Obtained	
K0873		8683	*Xanthomonas*	*melonis*					New Zealand	J. Young						P	Obtained	
K0874		16774	*Xanthomonas*	*cynarae*					New Zealand	J. Young						P	Obtained	
K0875		16370	*Xanthomonas*	*strains from Dysoxylum*					New Zealand	J. Young						P	Obtained	
K0876		570	*Xanthomonas*	*pisi*					New Zealand	J. Young						A	Obtained	

K0877		3490	*Xanthomonas*	*vasicola*					New Zealand	J. Young						P	Obtained	
K0878		5726	*Xanthomonas*	*arboricola*		*corylina*			New Zealand	J. Young						P	Obtained	
K0879		4779	*Xanthomonas*	*axonopodis*		*vesicatoria*			New Zealand	J. Young						P	Obtained	
K0880		8686	*Xanthomonas*	*melonis*					New Zealand	J. Young						P	Obtained	
K0881		16776	*Xanthomonas*	*cynarae*					New Zealand	J. Young						P	Obtained	
K0882		16468	*Xanthomonas*	*strains from Dysoxylum*					New Zealand	J. Young						P	Obtained	
K0883		35	*Xanthomonas*	*arboricola*		*arboricola*			New Zealand	J. Young						P	Obtained	
K0884		574	*Xanthomonas*	*campestris*		*incanae*			New Zealand	J. Young						P	Obtained	
K0885		3125	*Xanthomonas*	*oryzae*		*oryzae*			New Zealand	J. Young						P	Obtained	
K0886		696	*Xanthomonas*	*vesicatoria*					New Zealand	J. Young						A	NotObtained	
K0887		8684	*Xanthomonas*	*melonis*					New Zealand	J. Young						P	Obtained	
K0888		12004	*Xanthomonas*	*vasicola*					New Zealand	J. Young						P	Obtained	
K0889		5715	*Xanthomonas*	*fragariae*					New Zealand	J. Young						P	Obtained	
K0890		6465	*Xanthomonas*	*strains from Dysoxylum*					New Zealand	J. Young						P	Obtained	
K0891		50	*Xanthomonas*	*axonopodis*		*axonopodis*			New Zealand	J. Young						P	Obtained	
K0892		579	*Xanthomonas*	*hortorum*		*taraxaci*			New Zealand	J. Young						P	Obtained	
K0893		451	*Xanthomonas*	*vasicola*					New Zealand	J. Young						P	Obtained	
K0894		8923	*Xanthomonas*	*arboricola*		*populi*			New Zealand	J. Young						P	Obtained	

K0895		12013	*Xanthomonas*	*oryzae*		*oryzicola*			New Zealand	J. Young						P	Obtained	
K0896		16372	*Xanthomonas*	*strains from Dysoxylum*					New Zealand	J. Young						P	Obtained	
K0897		203	*Xanthomonas*	*cucurbitae*					New Zealand	J. Young						P	Obtained	
K0898		4319	*Xanthomonas*	*hortorum*		*pelargonii*			New Zealand	J. Young						P	Obtained	
K0899		5834	*Xanthomonas*	*axonopodis*		*phaseoli*			New Zealand	J. Young						P	Obtained	
K0900		16466	*Xanthomonas*	*strains from Dysoxylum*					New Zealand	J. Young						P	Obtained	
K0901		204	*Xanthomonas*	*cassavae*					New Zealand	J. Young						P	Obtained	
K0902		1404	*Xanthomonas*	*campestris*		*raphani*			New Zealand	J. Young						P	Obtained	
K0903		5741	*Xanthomonas*	*axonopodis*		*manihotis*			New Zealand	J. Young						P	Obtained	
K0904		5797	*Xanthomonas*	*fragariae*					New Zealand	J. Young						P	Obtained	
K0905		8679	*Xanthomonas*	*albilineans*					New Zealand	J. Young						P	Obtained	
K0906		9513	*Xanthomonas*	*codiaei*					New Zealand	J. Young						P	Obtained	
K0907		12545	*Xanthomonas*	*bromi*					New Zealand	J. Young						P	Obtained	
K0908		16473	*Xanthomonas*	*strains from Dysoxylum*					New Zealand	J. Young						P	Obtained	
K0909		5757	*Xanthomonas*	*axonopodis*		*vasculorum*			New Zealand	J. Young						P	NotObtained	
K0910		5743	*Xanthomonas*	*oryzae*		*oryzicola*			New Zealand	J. Young						P	Obtained	
K0911		16689	*Xanthomonas*	*gardneri*					New Zealand	J. Young						P	Obtained	
K0912		1590	*Xanthomonas*	*albilineans*					New Zealand	J. Young						A	NotObtained	

K0913		10041	*Xanthomonas*	*albilineans*					New Zealand	J. Young						A	NotObtained	
K0914		9894	*Xanthomonas*	*campestris*					New Zealand	J. Young						A	NotObtained	
K0915		4697	*Xanthomonas*	*axonopodis*					New Zealand	J. Young						A	NotObtained	
K0916		12326	*Xanthomonas*	*oryzae*		*oryzae*			New Zealand	J. Young						A	NotObtained	
K0917		2415	*Xanthomonas*	*species*					New Zealand	J. Young						A	NotObtained	
K0918		51	*Xanthomonas*	*arboricola*		*pruni*			New Zealand	J. Young						A	NotObtained	
K0919		8681	*Xanthomonas*	*axonopodis*		*axonopodis*			New Zealand	J. Young						A	NotObtained	
K0920		24	*Xanthomonas*	*axonopodis*		*citri*			New Zealand	J. Young						A	NotObtained	
K0921		21	*Xanthomonas*	*axonopodis*		*citri*			New Zealand	J. Young						A	NotObtained	
K0922		5732	*Xanthomonas*	*axonopodis*		*glycines*			New Zealand	J. Young						A	NotObtained	
K0923		5739	*Xanthomonas*	*axonopodis*		*malvacearum*			New Zealand	J. Young						A	NotObtained	
K0924		172	*Xanthomonas*	*axonopodis*		*vesicatoria*			New Zealand	J. Young						A	NotObtained	
K0925		109	*Xanthomonas*	*axonopodis*		*vesicatoria*			New Zealand	J. Young						A	NotObtained	
K0926		333	*Xanthomonas*	*axonopodis*		*vignicola*			New Zealand	J. Young						A	NotObtained	
K0927		8667	*Xanthomonas*	*cassavae*					New Zealand	J. Young						A	NotObtained	
K0928		8666	*Xanthomonas*	*cassavae*					New Zealand	J. Young						A	NotObtained	
K0929		13	*Xanthomonas*	*campestris*		*campestris*			New Zealand	J. Young						A	NotObtained	
K0930		9512	*Xanthomonas*	*codiaei*					New Zealand	J. Young						A	NotObtained	

K0931		2179	*Xanthomonas*	*cucurbitae*					New Zealand	J. Young						A	NotObtained	
K0932		6646	*Xanthomonas*	*fragariae*					New Zealand	J. Young						A	NotObtained	
K0933		4321	*Xanthomonas*	*hortorum*		*pelargonii*			New Zealand	J. Young						A	NotObtained	
K0934		188	*Xanthomonas*	*hyacinthi*					New Zealand	J. Young						A	NotObtained	
K0935		187	*Xanthomonas*	*hyacinthi*					New Zealand	J. Young						A	NotObtained	
K0936		8682	*Xanthomonas*	*melonis*					New Zealand	J. Young						A	NotObtained	
K0937		189	*Xanthomonas*	*hyacinthi*					New Zealand	J. Young						A	NotObtained	
K0938		190	*Xanthomonas*	*hyacinthi*					New Zealand	J. Young						A	NotObtained	
K0939		8689	*Xanthomonas*	*melonis*					New Zealand	J. Young						A	NotObtained	
K0940		9893	*Xanthomonas*	*populi*					New Zealand	J. Young						A	NotObtained	
K0941		5816	*Xanthomonas*	*populi*					New Zealand	J. Young						A	NotObtained	
K0942		6774	*Xanthomonas*	*theicola*					New Zealand	J. Young						A	NotObtained	
K0943		5733	*Xanthomonas*	*translucens*		*graminis*			New Zealand	J. Young						A	NotObtained	
K0944		5752	*Xanthomonas*	*translucens*		*translucens*			New Zealand	J. Young						A	NotObtained	
K0945		5755	*Xanthomonas*	*translucens*		*undulosa*			New Zealand	J. Young						A	NotObtained	
K0946		7291	*Xanthomonas*	*theicola*					New Zealand	J. Young						A	NotObtained	
K0947		63	*Xanthomonas*	*vesicatoria*					New Zealand	J. Young						A	NotObtained	
K0948		7294	*Xanthomonas*	*theicola*					New Zealand	J. Young						A	NotObtained	

K0950	A5630	OL-1	*Clavibacter*						Canada	Kristine Blum						P	Obtained	
K0951	A5631	OL-2	*Clavibacter*						Canada	Kristine Blum						P	Obtained	
K0952	A5632	OL-3	*Clavibacter*						Presidio County, Texas, USA	Kristine Blum						P	Obtained	
K0953	A5633	OL-4	*Clavibacter*						Presidio County, Texas, USA	Kristine Blum						P	Obtained	
K0954	A5634	OL-5	*Clavibacter*						Presidio County, Texas, USA	Kristine Blum						P	Obtained	
K0955	A5635	OL-6	*Clavibacter*						Marfa, Texas, USA	Kristine Blum						P	Obtained	
K0956	A5636	OL-7	*Clavibacter*						Presidio County, Texas, USA	Kristine Blum						P	Obtained	
K0957	A5637	OL-8	*Clavibacter*						Fort Davis, Texas, USA	Kristine Blum						P	Obtained	
K0958	A5638	OL-9	*Clavibacter*						Presidio County, Texas, USA	Kristine Blum						P	Obtained	
K0959	A5639	OL-10	*Clavibacter*						Marfa, Texas, USA	Kristine Blum						P	Obtained	

We used 840 strains from the PBC, 84 strains from the ICMP, 10 uncharacterized *Clavibacter* strains from a recent tomato canker outbreak in this study. The 263 strains for which ITS amplification and sequencing was attempted are identified by a ‘P’ or ‘A’ in the *ITS amplicon* column. The K number was the principal identifier for each strain in this study. The A number is the PBC accession number and the other numbers refer to original identification numbers for each strain. Strains of *Pectobacterium* and *Pantoea* that require a 51°C annealing temperature for amplification of the RIF marker are marked in the column RIF *Pectobacterium* amplification at 51°C.
