# Supplementary material for: Classification of Plant Associated Bacteria Using RIF, a Computationally Derived DNA Marker
Source: PLoS One. 2011 Apr 21;6(4):e18496. doi: 10.1371/journal.pone.0018496 (PMC3080875; doi:10.1371/journal.pone.0018496)
Supplement: Table S3 — X. albilineans is more distantly related to other Xanthomonas species than is Stenotrophomonas maltophilia . (PDF) [file pone.0018496.s008.pdf]

Supplemental Table S3. *X. albilineans* is more distantly related to other *Xanthomonas* species than is *Stenotrophomonas maltophilia*.

	S. maltophilia	*X. campestris*	*X. axonopodis*	*X. vesicatoria*	*X. melonis*	*X. cucurbitae*	*X.* sp. from *Dysoxylum*	*X. albilineans*	*X. codiaei*	*X. oryzae*	*X. vasicola*	*X. cassavae*	*X. fragariae*	*X. bromi*
X. campestris	71.679													
X. axonopodis	65.058	39.02												
X. vesicatoria	71.722	38	34.895											
X. melonis	77.444	34.944	36.158	46.5										
X. cucurbitae	69.667	45.722	31.158	38.5	40									
X. sp. from *Dysoxylum*	76.556	41.833	43.579	28.5	49	47								
X. albilineans	73.63	76.037	73.702	76.5	76.667	70.667	84							
X. codiaei	68.778	42.611	35.632	38.5	43	31	48	74.667						
X. oryzae	72.204	46.769	33.833	44.833	40.333	40.667	51.333	85.389	47.5					
X. vasicola	77.056	43	32.553	43	40.5	44.5	52.5	87.5	49.5	31.333				
X. cassavae	70.111	41.5	36.053	42	40	33	40.5	76.667	35	48	48.5			
X. fragariae	75.667	43.889	38.789	43.5	45	38	44.5	78.667	45	45.333	40.5	40		
X. bromi	71.111	36.111	36.211	26.5	41	43	31.5	77.667	37	46	39.5	37	41	
X. pisi	72.889	44.667	35.263	33.5	47	42	36	79.333	38	47	45.5	45	42	28*

RIF sequences were trimmed to 555 nucleotides among 128 different RIF sequences from characterized strains of *Xanthomonas*, *Ralstonia*, *Clavibacter*, *Dickeya* and *Pectobacterium* present in each major clade including sequenced strains from NCBI. Only RIF sequences from monophyletic groups were used, e.g. *X. cassavae* and *X. codiaei* and *X. cucurbitae* in clade M but not *X. hortorum* in clade L in Figure 3. The polyphyletic clade A included the species *X. perforans, X. citri, X. fuscans, X. alfalfae, X. euvesicatoria;* nevertheless, we

retained the name *X. axonopodis* because all of these strains were previously considered *X. axonopodis* pathovars. Species with divergent RIF sequences were split into different groups, e.g. the three clades containing *Dickeya dadantii* (C, E and F) in Figure 7. The average nucleotide distance between *X*. *albilineans* and other species of *Xanthomonas* (yellow) is greater than the distance between *S*. *maltophilia* and species of *Xanthomonas* excluding *X*. *albilineans* (orange).

***-** Species that are separated by unexpectedly small distances.
