# Supplementary material for: Classification of Plant Associated Bacteria Using RIF, a Computationally Derived DNA Marker
Source: PLoS One. 2011 Apr 21;6(4):e18496. doi: 10.1371/journal.pone.0018496 (PMC3080875; doi:10.1371/journal.pone.0018496)
Supplement: Table S4 — Average between species distances of the RIF marker from twenty-three different Ralstonia RIF sequences. (PDF) [file pone.0018496.s009.pdf]

Supplemental Table S4. Average between species distances of the RIF marker from twenty-three different *Ralstonia* RIF sequences.

	R. solanacearum	*R. eutropha*	*R. metallidurans*
R. eutropha	56.4		
R. metallidurans	55.4	55	
R. pickettii	44.2	65	60

Please see Supplemental Table S3 for details regarding the groupings.
