# Supplementary material for: Classification of Plant Associated Bacteria Using RIF, a Computationally Derived DNA Marker
Source: PLoS One. 2011 Apr 21;6(4):e18496. doi: 10.1371/journal.pone.0018496 (PMC3080875; doi:10.1371/journal.pone.0018496)
Supplement: Table S5 — Average between group distances of the RIF marker from ten different Pectobacterium RIF sequences. (PDF) [file pone.0018496.s010.pdf]

Supplemental Table S5. Average between group distances of the RIF marker from ten different *Pectobacterium* RIF sequences.

	P. atrosepticum	*P. carotovorum*	*P. atrosepticum*-oddA (*P. wasabiae*)	*P. atrosepticum*-oddB
P. carotovorum	50.8			
P. atrosepticum-oddA (*P. wasabiae*)	50.5	45.3		
P. atrosepticum-oddB	54.5	47	51	
P. carotovorum-oddA	122	110.3	118	111

P. atrosepticum-oddA refer to strains K0574 and Pw_Wpp163, *P. atrosepticum*-oddB refer to strains K0572 and K0573 and *P. carotovorum*-oddA refers to strain K0509 (See Figure 7). Please see Supplemental Table S3 for details regarding the groupings.
