# Supplementary material for: Classification of Plant Associated Bacteria Using RIF, a Computationally Derived DNA Marker
Source: PLoS One. 2011 Apr 21;6(4):e18496. doi: 10.1371/journal.pone.0018496 (PMC3080875; doi:10.1371/journal.pone.0018496)
Supplement: Table S6 — Average between group distances of the RIF marker from ten different Dickeya RIF sequences. (PDF) [file pone.0018496.s011.pdf]

Supplemental Table S6. Average between group distances of the RIF marker from ten different *Dickeya* RIF sequences.

	D. dadantii-A	*D. dadantii*-B	*D. dadantii*-C	*D. dianthicola*	*D. dieffenbachiae*	*D. zeae*-A
D. dadantii-B	93					
D. dadantii-C	61	94				
D. dianthicola	67	99	34*			
D. dieffenbachiae	62	92	7*	35*		
D. zeae-A	25.5*	95	67	74	68	
D. zeae-B	68	90	56	61	59	72

D. dadantii-A refers to Dd_586, *D. dadantii*-B refers Dd_703 and *D. dadantii-C* refers to strain K0494 and Dd_3937, *D. zeae*-A refers to strains K0550 and K0551, *D. zeae*-B refers to Dz_1591 (See Figure 7). Please see Supplemental Table S3 for details regarding the groupings.

***-** Species that are separated by unexpectedly small distances.
