# Supplementary material for: Classification of Plant Associated Bacteria Using RIF, a Computationally Derived DNA Marker
Source: PLoS One. 2011 Apr 21;6(4):e18496. doi: 10.1371/journal.pone.0018496 (PMC3080875; doi:10.1371/journal.pone.0018496)
Supplement: Table S7 — Average between subspecies distances of the RIF sequence from eleven different Clavibacter michiganensis RIF sequences. (PDF) [file pone.0018496.s012.pdf]

Supplemental Table S7. Average between subspecies distances of the RIF sequence from eleven different *Clavibacter michiganensis* RIF sequences.

	C. m. subsp. *insidiosus*	*C. m.* subsp. *michiganensis*
C. m. subsp. *michiganensis*	31.6	
C. m. subsp. *sepedonicus*	30	31.3

Please see Supplemental Table S3 for details regarding the groupings.
