# Supplementary material for: Classification of Plant Associated Bacteria Using RIF, a Computationally Derived DNA Marker
Source: PLoS One. 2011 Apr 21;6(4):e18496. doi: 10.1371/journal.pone.0018496 (PMC3080875; doi:10.1371/journal.pone.0018496)
Supplement: Table S8 — In silico comparison of Xanthomonas with the RIF marker resolves one pair of closely related strains that is unresolved with four other housekeeping genes and the ITS. (PDF) [file pone.0018496.s013.pdf]

Supplemental Table S8. ***In silico*** **comparison of** ***Xanthomonas*** **with the RIF marker resolves one pair of closely related strains that is unresolved with four other housekeeping genes and the ITS.**

Strain1	Strain2	RIF	*rpoD*	*dnaK*	*fyuA*	*gyrB*	ITS
Xcc_33913	Xcc_8004	5	0	0	0	0	0
Xcc_33913	Xcc_B100	12	41	2	1	1	1
Xcc_33913	Xe_85-10	66	60	40	88	106	18
Xcc_33913	Xccit_306	71	64	36	89	101	15
Xcc_33913	Xoo_PXO99	79	66	40	89	98	6
Xcc_33913	Xoo_10331	79	66	40	90	99	5
Xcc_33913	Xoo_311018	80	66	39	89	103	5
Xcc_33913	Sm_279	118	181	97		243	82
Xcc_33913	Sm_R551	122	181	101		237	85
Xcc_8004	Xcc_B100	17	41	2	1	1	1
Xcc_8004	Xe_85-10	69	60	40	88	106	18
Xcc_8004	Xccit_306	74	64	36	89	101	15
Xcc_8004	Xoo_PXO99	82	66	40	89	98	6
Xcc_8004	Xoo_10331	82	66	40	90	99	5
Xcc_8004	Xoo_311018	83	66	39	89	103	5
Xcc_8004	Sm_279	123	181	97		243	82
Xcc_8004	Sm_R551	127	181	101		237	85
Xcc_B100	Xe_85-10	66	47	42	87	107	19
Xcc_B100	Xccit_306	72	52	38	88	102	14
Xcc_B100	Xoo_PXO99	79	52	42	88	99	5
Xcc_B100	Xoo_10331	79	52	41	89	104	4
Xcc_B100	Xoo_311018	80	52	41	88	104	4
Xcc_B100	Sm_279	117	188	99		244	83
Xcc_B100	Sm_R551	123	183	103		238	86
Xe_85-10	Xccit_306	35	21	19	28	26	7
Xe_85-10	Xoo_PXO99	55	39	35	49	58	21
Xe_85-10	Xoo_10331	55	39	35	50	57	20
Xe_85-10	Xoo_311018	56	39	34	49	63	20
Xe_85-10	Sm_279	96	187	100		232	88
Xe_85-10	Sm_R551	103	182	107		234	90
Xccit_306	Xoo_PXO99	65	37	34	58	56	15
Xccit_306	Xoo_10331	65	37	34	59	55	14
Xccit_306	Xoo_311018	66	37	33	58	60	14
Xccit_306	Sm_279	107	186	99		227	88
Xccit_306	Sm_R551	113	183	103		238	89
Xoo_PXO99	Xoo_10331	0	0	2	1	1	0
Xoo_PXO99	Xoo_311018	1	0	1	0	10	0
Xoo_PXO99	Sm_279	115	184	100		235	85

Xoo_PXO99	Sm_R551	119	176	102		239	86
Xoo_10331	Xoo_311018	1	0	1	1	11	0
Xoo_10331	Sm_279	115	184	100		236	84
Xoo_10331	Sm_R551	119	176	101		242	85
Xoo_311018	Sm_279	116	184	99		238	84
Xoo_311018	Sm_R551	120	176	101		242	85
Sm_279	Sm_R551	34	61	25		63	13

The four markers based on the housekeeping genes *rpoD*, *dnaK*, *fyuA*, *and gyrB* [25] were extracted from each accession (Table 1) based on gene name. Nucleotide differences between strain1 and strain2 are indicated under the representative marker. RIF provided greater resolution than five different markers for strains Xcc_33913 and Xcc_8004. In contrast, Xoo_PXO99 and Xoo_10331 were not resolved by RIF but the strains were resolved by three other markers.
