# Supplementary material for: Classification of Plant Associated Bacteria Using RIF, a Computationally Derived DNA Marker
Source: PLoS One. 2011 Apr 21;6(4):e18496. doi: 10.1371/journal.pone.0018496 (PMC3080875; doi:10.1371/journal.pone.0018496)
Supplement: Text S1 — Computational derivation of a universal DNA marker from fifteen completely sequenced genomes was unsuccessful. (DOC) [file pone.0018496.s014.doc]

**Supplemental Text S1. Computational identification of a DNA marker with universal primers.**

An attempt to identify a universal marker was performed exactly as described in the section on “Computational identification of suitable marker regions” (see Methods), except that a search for conserved, unique oligos was performed using six completely sequenced genomes representing the three Gram^-^ genera *Xanthomonas* (Xccit_306, Xe_85-10, Xcc_8004), *Ralstonia* (Rs_GMI1000), *Pectobacterium* (Pa_SCRI1043) and the Gram^+^ *Clavibacter* (Cmm_32).  Only one 20-mer was conserved, but was not unique, among all six genomes (gcgtctaccaattccgccac). The *R*. *solanacearum* Rs_GMI1000 genome contained one copy on the chromosome and one copy on the megaplasmid. Therefore, no pair of single-copy 20^+-^mers separated by 550-1000 nt was conserved between all strains, thus no universal primer pair could be developed.
